# Identification of Salt Tolerance-related microRNAs and Their Targets in Maize (*Zea mays* L.) Using High-throughput Sequencing and Degradome Analysis

**DOI:** 10.3389/fpls.2017.00864

**Published:** 2017-05-26

**Authors:** Rong Fu, Mi Zhang, Yinchuan Zhao, Xuechuan He, Chenyun Ding, Shuangkuai Wang, Yan Feng, Xianliang Song, Ping Li, Baohua Wang

**Affiliations:** ^1^Scientific Observing and Experimental Station of Maize in Plains Area of Southern Region, Ministry of Agriculture and School of Life Sciences, Nantong UniversityNantong, China; ^2^State Key Laboratory of Crop Biology, College of Agronomy, Shandong Agricultural UniversityTai’an, China

**Keywords:** maize, salt tolerance, miRNA, high-throughput sequencing, degradome, qRT-PCR

## Abstract

To identify the known and novel microRNAs (miRNAs) and their targets that are involved in the response and adaptation of maize (*Zea mays*) to salt stress, miRNAs and their targets were identified by a combined analysis of the deep sequencing of small RNAs (sRNA) and degradome libraries. The identities were confirmed by a quantitative expression analysis with over 100 million raw reads of sRNA and degradome sequences. A total of 1040 previously known miRNAs were identified from four maize libraries, with 762 and 726 miRNAs derived from leaves and roots, respectively, and 448 miRNAs that were common between the leaves and roots. A total of 37 potential new miRNAs were selected based on the same criteria in response to salt stress. In addition to known miR167 and miR164 species, novel putative miR167 and miR164 species were also identified. Deep sequencing of miRNAs and the degradome [with quantitative reverse transcription polymerase chain reaction (qRT-PCR) analyses of their targets] showed that more than one species of novel miRNA may play key roles in the response to salinity in maize. Furthermore, the interaction between miRNAs and their targets may play various roles in different parts of maize in response to salinity.

## Introduction

In plant–environment coevolution, plants mount a series of regulatory mechanisms in the cellular, physiological, biochemical, and molecular processes to protect themselves from different kinds of unfavorable conditions (Covarrubias and Reyes, 2010). High salinity is one of the most wide-ranging and severe abiotic stresses that restrains plant growth and development, and negatively affects plant yield and product quality (Krasensky and Jonak, 2012). Many studies suggest that differing levels of expression for specific genes is a significant strategy for plants to combat salinity stress at the post-transcriptional stage (Covarrubias and Reyes, 2010; Huang et al., 2012).

Maize is one of the most important crops in the world for its role in food, feed, and biofuel. At the same time, it is a vital material for the research of genetics and genomics in higher plants (Schnable et al., 2009). Currently, an increasing number of studies are being conducted on maize that include the mechanisms of crop development and the environmental adaption of crops to salt to improve quality and yield (Farooq et al., 2015; Zurek et al., 2015). In a study by Cui et al. (2016), the grain yield decreased with an increase in salinity, and with a salinity under 1.5 g/kg soil, the yields of ‘Suyu 10’ and ‘Suyu 30’ (two hybrid lines with salt tolerance) were 54.2 and 64.3%, respectively, of those without salt stress.

Small RNA (sRNA) is a special kind of molecule in organisms that induces gene silencing and plays an important role in the regulation of cell growth, gene transcription, and translation. sRNAs also contribute to responses to biotic and abiotic stresses such as aridity, salinity, malnutrition, abscisic acid (ABA), and low temperature (Phillips et al., 2007; Reyes and Chua, 2007; Liu et al., 2008; Shukla et al., 2008; Xu Z. et al., 2011; Qin et al., 2015; Yadav et al., 2016). sRNAs from HiSeq deep sequencing cover nearly every kind of RNA, including microRNA (miRNA), small interfering RNA (siRNA), Piwi-interacting RNA (piRNA), ribosomal RNA (rRNA), transfer RNA (tRNA), small nuclear RNA (snRNA), and small nucleolar RNA (snoRNA). miRNA, siRNA, and piRNA (repeat-associated sRNA and degraded tags of an exon or intron) are hot topics in the research field of sRNA (Hafner et al., 2008). miRNAs are a family of 21–24 nucleotide (nt) sRNAs that regulate the post-transcriptional gene expression by miRNA-mediated cleavage, mRNA destabilization, or translational repression when partially or completely complementary to target mRNAs (Bartel, 2004; Mallory and Bouche, 2008). It has been reported that miRNAs are potential targets for abiotic stress tolerance in plants (Shriram et al., 2016). Differential expression patterns of miRNAs were observed under different abiotic stresses, including salinity, drought, high temperature, cold, cadmium, and arsenic, in many plants (Xie et al., 2014; Ferdous et al., 2015; Giusti et al., 2016; Shriram et al., 2016; Wang et al., 2016). The effects of miRNAs on improving plant tolerance to abiotic stresses were recently confirmed in rice and *Arabidopsis* via altering the miRNA expression in transgenic plants (Shriram et al., 2016).

By comparing newly generated sequences with those in databases and to find the overlap in genome location between new data and databases, miRNA can be annotated into different categories. Those sequences that cannot be annotated will be used to predict novel miRNAs by the self-developed software Mireap. Based on the result of miRNA, differential expression analysis of miRNA can be performed to obtain the difference between different periods, tissues, and individuals using a flexible method that depends on the specificity of the samples (Allen et al., 2005; Schwab et al., 2005). Target prediction for the above different miRNAs will be analyzed to confirm target sites, and then the Gene Ontology (GO) enrichment and Kyoto Encyclopedia of Genes and Genomes (KEGG) pathway will be annotated (Kanehisa et al., 2008). Based on the above analysis, a clear biological information map of the miRNAs that regulate many key biological processes will then be obtained.

Currently, a high-throughput method with next-generation sequencing technology is able to profile and quantify mRNA cleaved fragments (Addo-Quaye et al., 2008). Using an Illumina sequencing system, degradome sequencing is a powerful and efficient approach for detecting and validating miRNA–mRNA pairs. Degradome sequencing provides a comprehensive means of analyzing patterns of RNA degradation. Degradome sequencing has been used to identify miRNA cleavage sites because miRNAs function via base-pairing with complementary sequences within mRNA molecules, and usually result in gene silencing via translational repression or target degradation (Addo-Quaye et al., 2009). Degradome sequencing has revealed many known and novel plant miRNA (siRNA) targets (Zhou et al., 2010; Yang and Wang, 2013).

In this study, to identify known and novel miRNAs and their targets involved in the response and adaptation of maize to salt stress, miRNAs and their targets were identified by a combined analysis of the deep sequencing of sRNA and degradome libraries. The identity of target genes will be further confirmed by quantitative reverse transcription polymerase chain reaction (qRT-PCR) analyses.

## Materials and Methods

### Plant Material and Treatment

The uniform seeds of maize inbred line LH196 were selected for use. After 2 h of sunlight insolation, 1 h of water flushing, 8 min of immersion disinfection with 70% of alcohol, and 6 min of soaking disinfection with 10% H_2_O_2_, the seeds were washed with diluted water three times at 1 min each time. The seeds were transferred to a layer of filter paper in a petri dish and then covered with another wet filter paper sprayed with diluted water. Germination was accelerated under dark with a temperature of 28°C for 2 days in a plant growth chamber. The germinated seeds were then planted in plastic pots containing vermiculite and cultured under conditions of 14 h light/10 h dark with an air temperature of 30°C light/25°C dark in the plant growth chamber. At the stage of third true leaves, healthy and uniformly growing plants were selected and divided into two groups. These groups were transferred to a hydroponic device and then they were individually treated with different nutrient solutions including 0 mM NaCl (CK) and 250 mM NaCl (salt). The other components of the solution were the same as previously described (Xu Z. et al., 2011). The leaves and roots were harvested after 12 h of treatment, immediately frozen in liquid nitrogen and stored at -80°C for high-throughput sequencing and degradome analysis. Other seedlings were treated 10 days in nutrient solutions including 0 mM NaCl as CK or 250 mM NaCl as salt treatment before harvested for further phenotype measurement, with the nutrient solutions being replaced by new ones every day.

### Phenotype Measurement

After 10 days of treatment, we harvested the shoots and roots separately. The control group and the treatment group each contained nine randomly taken seedlings and the fresh weight and dry weight were recorded. Anthocyanin was extracted as follows: four seedlings of CK and salt treatment were harvested, respectively; the stems were weighed after the leaves and roots were removed; the stems were then cut into short pieces at length of 2–3 mm and they were put into an EP tube; 10 mL of extraction solution (HCl: methyl alcohol = 1.99, v/v) were added, and the lid was closed and kept at 4°C for 2 days; the tube was shaken twice each day. The relative content of the anthocyanin of the stems was calculated [relative contents of anthocyanin = (OD_530_-0.25 × OD_657_) × the volume of extract/the weight of stem] (Zhao, 2013) on an Excel spreadsheet.

### RNA Isolation and Purification

The total RNA was extracted from the four types of samples, namely, the leaf of CK (LC), the leaf under salt treatment (LS), the root of CK (RC), the root under salt treatment (RS) via Trizol reagent (Invitrogen, Carlsbad, CA, United States) according to the manufacturer’s protocol. The RNA concentration and purity were measured using a BioPhotometer (Eppendorf, HH, GER), and the RNA quality and integrity were assessed by agarose gel electrophoresis. The RNAs were treated with RNase-free DNase I (TaKaRa, Dalian, China) to remove the genomic DNA.

### Small RNA Library Construction, Sequencing, and Data Analysis

Four strand-specific RNA libraries with a size of 18–30 nt were prepared as previously described (Sunkar and Zhu, 2004; Hafner et al., 2008), and submitted to the BGI (Beijing Genomics Institute, Shenzhen, China) for sequencing. The sRNA digitalization analysis based on HiSeq high-throughput sequencing with the Illumina HiSeq 2000 sequencing system utilizes the sequencing by synthesis (SBS), which can decrease the loss of nucleotides caused by the secondary structure. It is also noted for its small sample requirement, high throughput, and high accuracy with a simply operated automatic platform. Through such analysis, one can obtain millions of sRNA sequence tags in one process, can comprehensively identify sRNAs of certain species in certain conditions, and predict novel miRNAs and construct a sRNA differential expression profile between samples, which is a powerful tool for sRNA function research. The experimental process of sRNA sequencing is shown in **Supplementary Figure S1**. The 49 nt sequence tags from HiSeq sequencing will undergo data cleaning analysis first, which includes getting rid of the low-quality tags and 5′ adaptor contaminants from the 50 nt tags to obtain credible clean tags. The length distribution of the clean tags and both the common and specific sequences between samples will be further summarized. A standard analysis will be performed to categorize the clean tags as potentially known miRNAs vs. novel miRNAs, which would be those which cannot be placed into any previously known category. After obtaining this miRNA result, target prediction for miRNAs, GO enrichment, and a KEGG pathway for target genes will be analyzed. The procedure is shown in **Supplementary Figure S2**. The undesirable reads were excluded from the raw data to generate clean reads. The sRNA tags were mapped to the genome by the Short Oligonucleotide Analysis Package (SOAP) (Li et al., 2008)^1^ to analyze their expression and distribution on the maize genome^2^. The sequences that were perfectly matched were identified for the next analysis. Non-coding RNAs (ncRNAs) that were annotated as rRNAs, tRNAs, snRNAs, and snoRNAs were identified by alignment and a BLAST search against the Rfam (10.1) and GenBank databases (Benson et al., 2013).

### Degradome Library Construction, Sequencing, and Data Analysis

Approximately 150 mg of total RNA were used for the purification of polyadenylated RNA molecules with oligo(dT) cellulose (Ambion). In addition, degradome libraries were built as previously described (German et al., 2008). By using the Illumina HiSeq 2000 sequencing system, degradome sequencing utilizes a SE50 sequencing strategy and produces 49 nt raw reads. The 3′ adaptor was trimmed before the bioinformatics analysis to obtain real degradome fragments with a length of 20–21 nt (Folkes et al., 2012). All of the data herein have been deposited in the National Center for Biotechnology Information, United States (NCBI). After pre-processing, the clean tags are generated and stored. Clean tags are classified by the alignment to the database and to remove the ncRNAs. Finally, the miRNA–mRNA pairs are identified and mapped to the reference genes. Clean tags are mapped to the genome by SOAP2.20 and then the genome distribution of the tags is analyzed (see **Supplementary Figure S3**) (German et al., 2008, 2009). Degradome sequencing provided the resource for us to predict the target genes, and the GO analysis of the entire target genes are shown in **Supplementary Figure S4**.

### qRT-PCR Analysis

The expression of miRNAs and their target genes are quantified by an iCycler iQTM real-time PCR detection system (Bio-Rad), following the manufacturer’s instructions. To accurately estimate the regulation by miRNA of its target gene, forward primer and reverse primers located in both sides of the predicted cleavage site were designed with Primer 3 software^3^. The cDNAs were reverse-transcribed from the prepared RNAs according to PrimeScript^TM^ RT Master Mix (TaKaRa) and they were then diluted into same concentration. qRT-PCR with three statistical and three biological repeats were run with the Power SYBR Green PCR Master Mix (Applied Biosystems). Maize a-tubulin 5 (Tub5) was selected to be an internal standard. A 2 μL volume of cDNA was used in the 25 μL PCR mixture. The mixture was incubated at 95°C for 3 min, followed by 50 cycles of 95°C for 15 s, and 60°C for 30 s.

## Results

### The Phenotypic Response of LH196 to Salt-Treatment

**FIGURE 1:**
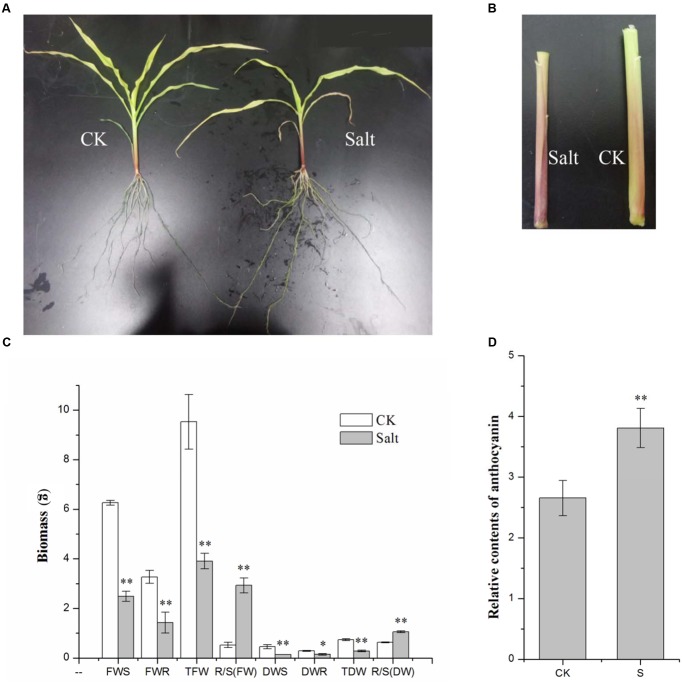
**Phenotypic changes of maize seedlings under salt stress.** **(A)** Phenotypic changes. **(B)** Anthocyanin accumulated in the roots. **(C)** Changes of biomass (FWS, fresh weight of the shoot; FWR, fresh weight of the root; TFW, total fresh weight; DWS, dry weight of the shoot; DWR, dry weight of the root; TDW, total dry weight). **(D)** Relative contents of anthocyanin (CK, control; S, salt). (^∗^*p* ≤ 0.05, ^∗∗^*p* ≤ 0.01).

Maize seedlings grown under a 250 mM NaCl solution treatment provided a phenotype of salt stress: the leaves turned yellow and wilted, the aerial roots grew to a large quantity, and the branches of the roots increased and became thinner (**Figure 1A**). All of the parts of the maize inbred line LH196 seedling biomass were significantly reduced, but both the root/shoot ratio of the fresh weight and dry weight increased significantly (**Figure 1C**). Under the salt treatment, the relative content of anthocyanin in the stem had a significant increase of approximately 1.5 times (**Figures 1B,D**).

### sRNAs and Degradome Sequences Generated across Libraries of Different Salt Treatments

**FIGURE 2:**
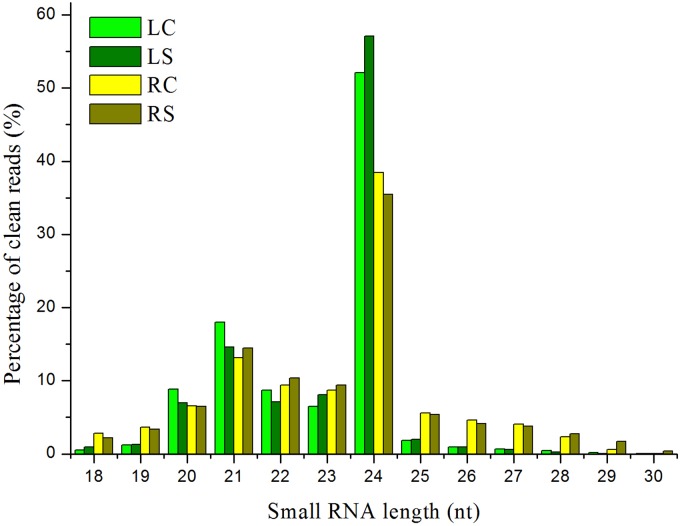
**Small RNA (sRNA) length distribution for four sequenced libraries.** LC, leaf control; LS, leaf salt; RC, root control; RS, root salt.

**FIGURE 3:**
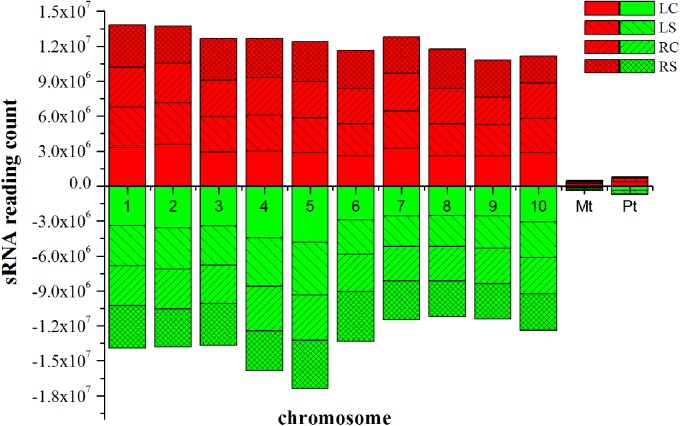
**Distribution of sRNAs on every chromosome. red indicates sense, green indicates antisense.** LC, leaf control; LS, leaf salt; RC, root control; RS, root salt.

Approximately 100.7 million raw reads of sRNA and degradome sequencing data were generated from the four libraries. The data involving the raw reads, clean reads, and unique clean reads and the matching sequences of the maize genome across the entire sequencing of the samples are listed in Supplementary Table S1. The similarity of the four maize sRNA libraries, leaf control, leaf salt, root control, and root salt (LC, LS, RC, and RS), is appreciable (see Supplementary Table S2). The unique matching sequences were used in further steps. All four sRNA libraries show a similar trend, which indicates that the 24 nt group has the highest abundance of size distribution according to the unique reads (**Figure 2**). The distribution of sRNAs on every chromosome is quite uniform (**Figure 3**), with the sRNA reading counts being close to each other. The abundance of extremely conserved miRNAs has been determined, such as miR156, miR159, miR164, miR166, miR167, miR168, and miR398, which was very similar to previous reports (Zhao et al., 2013). For instance, the abundance of zma-miR156 and zma-miR167 was 40560.15 reads per million (RPM = specific miRNA reads^∗^10^6^/total reads) and 3714.984 RPM, respectively.

Our sRNA sequencing data covered the known miRNAs very well. Combining two leaf libraries (LC, LS) with the other two root classes (RC, RS), a total of 81 unique mature miRNA sequences belonging to 25 known miRNA families in miRBase (Release 21: June 2014) (Kozomara and Griffiths-Jones, 2014) were sequenced, whereas in all of the libraries, there were 37 of 81 unique mature miRNAs detected.

The identification of new miRNAs supplemented the database of maize miRNA. Many potentially new miRNAs were identified from the four libraries, which have not been registered on the miRBase previously (Release 21). More than 200 novel miRNAs were found in the leaves, and approximately 150 potentially new miRNAs were detected in the roots. Seventy-six of them were identified in both the leaves and the roots (see Supplementary Table S3).

### Identification of Known miRNAs in Maize

It is well known that miRNAs are highly conserved in plants. According to the alignment of all of the unique clean reads with less than four mismatches from four libraries (LC, LS, RC, and RS) against all of the known plant miRNAs in miRBase (Release 21), a total of 1040 known plant miRNAs were identified from these four maize libraries; 762 and 726 were from the leaves and roots, respectively, and 448 miRNAs were common between the leaves and roots. Among these 1040 miRNAs, 314 and 278 miRNAs were specific to the leaves and roots, respectively. We used both the fold change (absolute value ≥ 1) and *P*-value (significant ^∗^, *P*-value ≤ 0.05; extremely significant ^∗∗^,*P*-value ≤ 0.01) to define the significance of the expression. A total of 400 (52.49%) and 455 (62.67%) miRNAs showed a significantly different expression in the leaves and roots, respectively, under different treatments (see Supplementary Table S4).

### Novel miRNA Expression in Response to Salt Treatment

**FIGURE 4:**
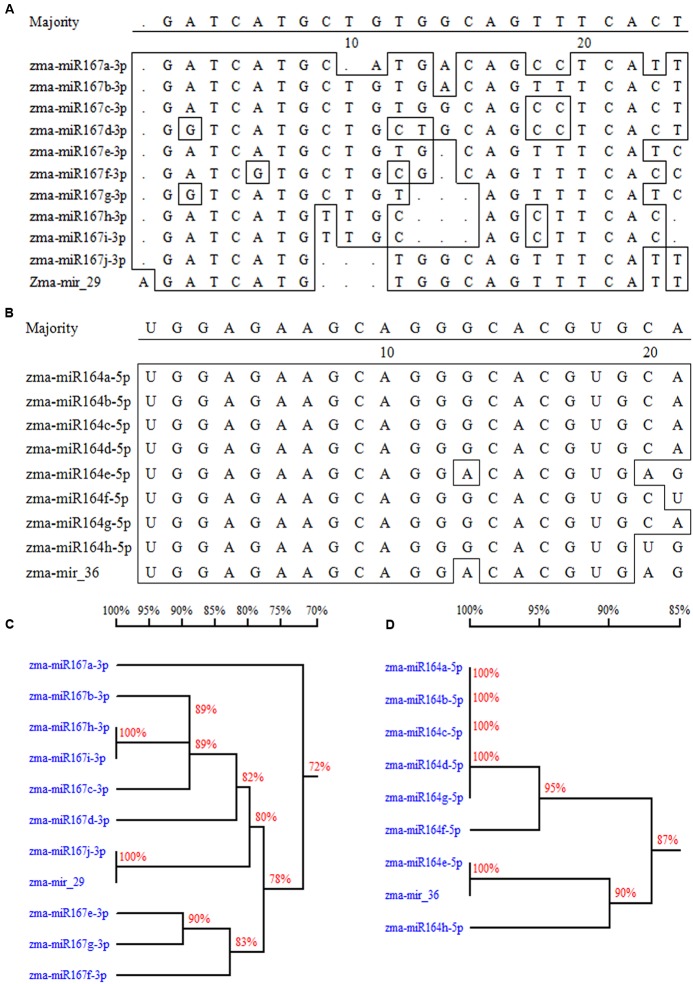
**Two novel miRNAs assigned into two miRNA families.** **(A)** The sequence comparison between mir-29 and other members in miR167 family. **(B)** The sequence comparison between mir-36 and other members in miR164 family. **(C)** Cluster analysis of miR167 family including the new member of mir-29. **(D)** Cluster analysis of miR164 family including the new member of mir-36.

A total of 37 potential new miRNAs were selected based on the criteria of an absolute fold change ≥ 2 between the salt treatment and the control (Zhao et al., 2013) and standardized sequencing reads ≥ 1 RPM (see Supplementary Table S5). Fifteen of the 37 miRNAs showed significant differences between the salt treatment and the control in both leaves and roots; 13 showed significant difference only in the leaves, whereas 9 showed significant difference only in the roots. Among these potential miRNAs, mir-29 and mir-36 were classified as new members of the miR167 family and miR164 family, respectively (**Figure 4**). Many novel miRNAs identified from two or more sRNA libraries also have a relatively high abundance, and their expression levels changed significantly under the salt treatment. For instance, mir-250 was significantly down-regulated in both the leaves and roots under the salt treatment, whereas mir-330 was up-regulated significantly in the leaves but down-regulated in the roots (see Supplementary Table S5).

### Identification of Eight Novel miRNAs and Their Targets in Response to Salinity Stress

Among all of the novel miRNAs, mir-29 and mir-36 were classified as new members of the miR167 family and miR164 family, respectively. Four unique mature miRNA sequences were detected, namely, zma-miR167a, a-5p, zma-miR167e-3p and zma-miR164a-3p. In the miR167 family, mir-29 was the only significantly changed species, and in miR164s, mir-36 was up-regulated significantly under salt treatment in the leaves, whereas others were down-regulated (**Table 1**). The results suggest that mir-29 and mir-36 may play a primary role among zma-miR167 species and zma-miR164 species in responses to high salinity, respectively. The other six novel species, namely, mir-250, mir-316, mir-17, mir-330, mir-205, and mir-189, caught our attention. They showed a highly significant difference between the control and the salt treatment in the leaves and roots (**Table 1**). These eight novel miRNAs together with their targets and functions are listed in **Table 2**.

**Table 1:** Differentially expressed miRNAs in response to salt treatment in the maize seedlings.

Family	miR-name	Pairwise	Control-std	Salt-std	Sig-label
MIR164	miR164a-5p	LC–LS	1107.63	696.04	^∗∗^
		RC–RS	569.42	691.09	-
	miR164a-3p	LC–LS	1.26	0.01	^∗∗^
		RC–RS	1.96	2.76	-
	mir_36	LC–LS	168.4	433.12	^∗∗^
		RC–RS	-	-	-
MIR167	miR167a-5p	LC–LS	4934.6	3789.07	-
		RC–RS	2016.53	2968.53	-
	miR167e-3p	LC–LS	594.04	443.55	-
		RC–RS	158.78	221.68	-
	mir_29	LC–LS	52.49	49.88	-
		RC–RS	7.83	25.17	^∗∗^
	mir_250	LC–LS	13.19	0.01	^∗∗^
		RC–RS	3.4	0.01	^∗∗^
	mir_316	LC–LS	-	-	-
		RC–RS	1.62	3.47	^∗∗^
	mir_17	LC–LS	1.76	0.01	^∗∗^
		RC–RS	1.36	0.01	^∗∗^
	mir_330	LC–LS	0.01	4.12	^∗∗^
		RC–RS	4.08	0.01	^∗∗^
	mir_205	LC–LS	1.43	0.01	^∗∗^
		RC–RS	0.01	1.26	^∗∗^
	mir_189	LC–LS	2.44	0.01	^∗∗^
		RC–RS	0.01	1.34	^∗∗^


*LC, leaf control; LS, leaf salt; RC, root control; RS, root salt. ^∗∗^Indicates significant level of *p* < 0.01.*

**Table 2:** The identified novel miRNAs with their targets and functions.

miRNA	Targets	Functions annotations
mir_17	GRMZM2G154667	Translation initiation factor IF-1
mir_189	GRMZM2G141185	Hypothetical protein SORBIDRAFT_03g041060
	GRMZM2G130062	2-Isopropylmalate synthase B [EC:2.3.3.13]
	GRMZM2G123652	TPA: hypothetical protein
mir_205	GRMZM2G375504	p5cs isoform 1 [EC:2.7.2.11 1.2.1.41]
mir_250	GRMZM2G012479	Glutathione peroxidase [EC:1.11.1.9]
	GRMZM2G458728	DNA (cytosine-5-)-methyltransferase [EC:2.1.1.37]
mir_316	GRMZM2G320298	Gibberellin receptor GID1 [EC:3.-.-.-]
	GRMZM2G306345	Pyruvate, orthophosphate dikinase [EC:2.7.9.1]
	GRMZM2G154628	Aquaporin PIP2-4
mir_330	GRMZM2G046092	Casein kinase II subunit alpha [EC:2.7.11.1]
	GRMZM5G823004	Unknown
	GRMZM2G305167	Casein kinase II subunit alpha [EC:2.7.11.1]
	GRMZM2G012324	Photosystem I reaction center 6
	GRMZM2G017290	Photosystem I reaction center subunit III
	GRMZM5G874478	Glycine-rich RNA-binding protein 8
	GRMZM2G012160	Cysteine proteinase inhibitor
	GRMZM2G466743	Casein kinase II subunit alpha [EC:2.7.11.1]
mir_36	GRMZM2G149952	Unknown
	GRMZM2G055489	Sucrose-phosphatase 1 (SPP1)
mir_29	GRMZM2G110688	Transport protein SEC24
	GRMZM2G084296	PHD-finger family protein, phospholipase D
	GRMZM2G362718	TPA: putative EDM2-like family protein


### Reliability of miRNA Expression via qRT-PCR

**FIGURE 5:**
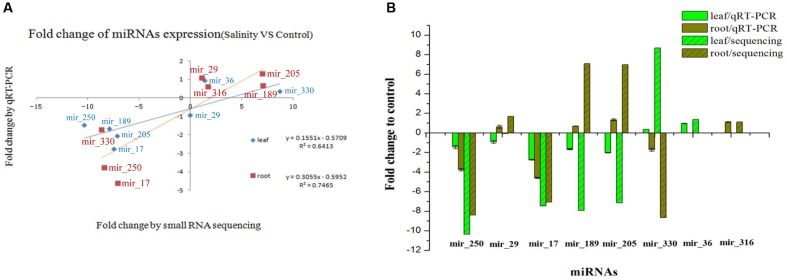
**Validation and comparison of the expression of eight novel miRNAs between qRT-PCR and sRNA sequencing.** **(A)** The miRNA expression correlation between qRT-PCR and sRNA sequencing (salinity vs. control). **(B)** The fold change of miRNA expression relative to the control sample. The bar represents the standard deviation.

Eight novel miRNAs were randomly selected for qRT-PCR to test the reliability of sRNA sequencing. Compared with the expression of the control miRNAs, the expression pattern of most miRNAs under the salt treatment by deep sequencing was similar to qRT-PCR in both the leaf and root, respectively (**Figure 5**). The correlation test showed that the expression of the eight miRNAs exhibited significant positive correlations between qRT-PCR and deep sequencing (*R*^2^ = 0.6413; and *R*^2^ = 0.7465) (**Figure 5A**).

### Negative Regulation by Novel miRNAs

**FIGURE 6:**
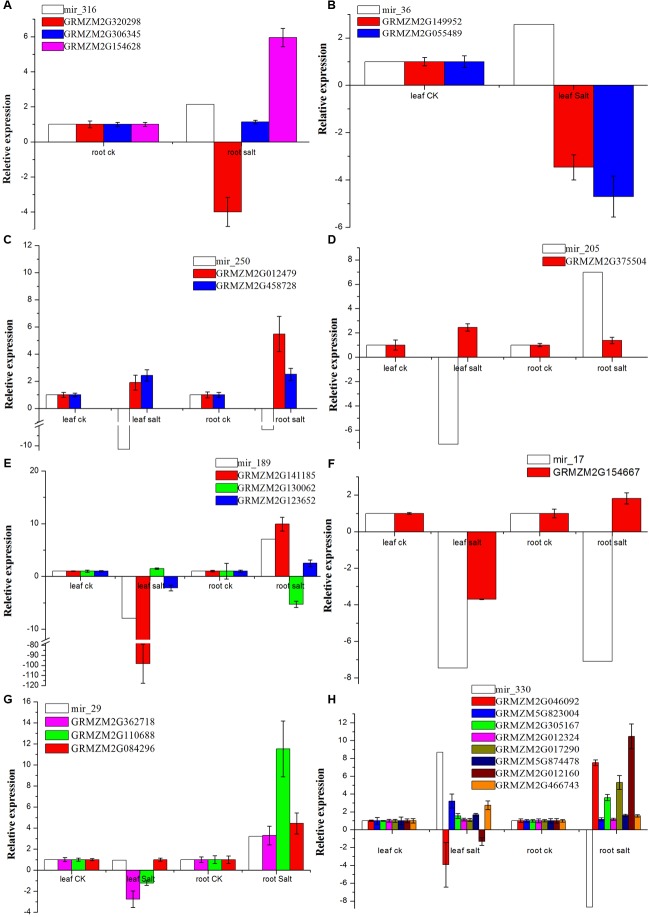
**Expression of targets vs. eight novel miRNAs by qRT-PCR.** **(A–H)** Indicates the expression of targets for mir_316, mir_36, mir_250, mir_205, mir_189, mir_17, mir_29 and mir_330, respectively. The relative expression levels for the target genes are the means of the fold changes and the standard deviation from three biological replicates.

**FIGURE 7:**
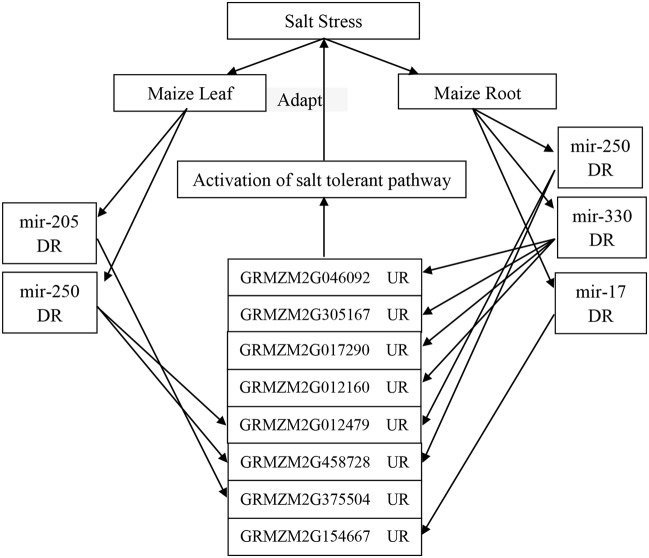
**A possible internal regulation pathway of four species of maize novel miRNAs in response to salt stress.** DR, down-regulation; UR, up-regulation. When the maize was under salt stress, the expression of four novel miRNAs (mir-250, mir-205, mir-330, and mir-17) is down-regulated in the leaves or roots of maize; then, their targets, such as casein kinase II, Gpx, P5CS, IF-1, and some other genes are up-regulated, and thereafter, some salt tolerant pathways are activated to adapt to salt stress.

We examined the response of the targets of the eight novel miRNAs (mir-29, mir-36, mir-205, mir-250, mir-316, mir-330, mir-17, and mir-189) to salt treatment in the leaves, roots or both by qRT-PCR with specific primers (see Supplementary Table S6) with *Zea mays* alpha-tubulin 5 used as the internal standard. The relative expression levels of GRMZM2G012479, GRMZM2G046092, GRMZM2G017290, and GRMZM2G012160 before salt treatment were very low, but the four genes were 5.5-fold, 7.5-fold, 5.3-fold, and 10.5-fold up-regulated after salt treatment in the roots, respectively. Interestingly, GRMZM2G046092 and GRMZM2G012160 were down-regulated in leaves under the salt treatment. However, GRMZM2G130062, the target of novel mir-189, was up-regulated in the leaves but down-regulated more than fivefold in the roots. In addition, some targets were up-regulated or down-regulated both in the leaves and roots (**Figure 6**). These results suggest that the interaction between miRNAs and their targets may play various roles in different tissues of maize in response to salinity. Thus, in this study, a possible internal regulation pathway was proposed for four species of maize novel miRNAs in response to salt stress (**Figure 7**).

## Discussion

Salt stress has an important environmental impact on plants. Under salt treatment, maize seedlings were stressed. Furthermore, the relative content of anthocyanin in the stem has a significant increase under salt stress. This suggests that the relative content of anthocyanin can be used as a judgment index of the salt stress degree of maize seedlings, and it also shows that anthocyanin may participate in the salt stress response and adjust the adaptability to salinity in the environment.

Our sRNA sequencing results covered the known miRNA families and species in the maize well, and a batch of potential miRNAs in the maize was found at the same time. The size distribution of the fragments was consistent with previous reports regarding different maize tissues (Jiao et al., 2011; Wang et al., 2011; Zhao et al., 2013). For instance, the 24 nt class represented 38.42% of the clean reads from the control roots, whereas the clean reads of both the control and the treatment leaves are very high (over 50%) (**Figure 2**). The distribution pattern in leaves and roots may also explain the slight differences between the response of leaves and roots to salinity.

Differentially expressed known miRNAs are listed in Supplementary Table S4. Compared with the previous research of salt stress in maize (Ding et al., 2009), most known salinity-responsive miRNAs have been confirmed in our study. However, there was some differential expression of mature miRNAs under the salt treatment between the previous reports and ours. For example, miR162 was not found in our research; miR156, miR164, miR167, and miR396 were not down-regulated in the roots, but in the leaves, miR164 showed a down-regulation. These differences may be due to the different maize inbred lines used. In addition, the degradome sequencing data in this research provided in-depth target information on those identified mature miRNAs because the analysis of the degradome can detect 15–30% of the predicted targets and 50–80% percent of the previously validated targets (Addo-Quaye et al., 2008; German et al., 2008).

To assign a new species to a distinct miRNA family, researchers should consider the nearly identical or newly identified paralogous miRNA loci producing identical and mature miRNAs, sequence conservation of the novel miRNAs, and accurate excision from the stem of a stem–loop precursor (Nobuta et al., 2008). In this research, two identified novel miRNAs (mir-29 and mir-36) showed a high similarity with known miR164 and miR167 family species (**Figure 4**), respectively.

Eight novel miRNAs (mir-29, mir-36, mir-205, mir-250, mir-316, mir-330, mir-17, and mir-189) were selected for qRT-PCR to test the reliability of sRNA sequencing. However, *R*^2^ was slightly lower because the fold change was not quite the same as the different tested miRNAs, which is probably due, at least partially, to the sensitivity between the deep sequencing and qRT-PCR technology. Consequently, the validation based on qRT-PCR indicated that deep sequencing is reliable in quantifying miRNA expression abundance in maize. Overall, qRT-PCR showed a similar changing tendency with deep sequencing although the fold change of miRNA expression in the salinity vs. the control was lower than the changes in sRNA sequencing (**Figure 5**), which suggests the reliability of our sRNA sequencing results.

Many novel miRNAs identified from two or more sRNA libraries also have a relatively high abundance, and their expression levels changed significantly under salt treatment. This suggests that some detected novel miRNAs may play key roles in adapting to salt stress in maize. Furthermore, the results also offer a solid foundation for further examination of the types of regulatory roles novel miRNAs play in response or adaptation to high salinity.

In this research, miRNAs and their targets that are related to salt tolerance were identified. Interestingly, there are many identified miRNAs that are likely to play crucial roles with their targets as salinity. Translation initiation factors (Buso et al., 1999) have been widely studied in human and yeast (Chaudhuri et al., 1997; Battiste et al., 2000; Rausell et al., 2003). In addition to the synthesis of proteins, translation IFs play various other important roles in plants. IF1 can effectively improve plant resistance to drought, salinity, oxidation, heavy metals, and extreme temperature, and it may participate in a variety of processes of plant stress-resistance (Yang et al., 2014; Zhao et al., 2015). The eukaryotic initiation factor 1 (eIF1), eIF5, and eIF4 are necessary for tolerance to abiotic stresses such as salt, oxidation, high temperature, osmotic and nutrient stress, and some biotic stresses such as *Fusarium graminearum* and *Phytophthora sojae* (Xu J. et al., 2011; Dong et al., 2015; Martinez-Rocha et al., 2016). eIF1A, eIF2, and eIF4A have also been shown to improve stress tolerance (Dutt et al., 2015). In this study, novel miRNA mir_17 was down-regulated in both the leaves and roots after the salt treatment, and its target translation IF-1 (GRMZM2G154667) showed the opposite method of regulation to mir_17 (**Figure 6**). The up-regulated translation initiation may help maize tolerate salinity.

Pyrroline-5-carboxylate synthetase (P5CS) is a key regulatory enzyme that plays a crucial role in proline biosynthesis, and proline acts as an osmolyte that accumulates when plants are subjected to abiotic stress (Strizhov et al., 1997). Recently, a study showed that the expression of *P5CS* transgene resulted in the overproduction of the P5CS enzyme in transgenic chickpea plants and an increase in proline accumulation. The accumulation of osmoprotectants may increase tolerance to osmotic stress and sodium toxicity (Ghanti et al., 2011). Overexpression of P5CS also increased stress tolerance of transgenic potato, rice, wheat, sweet sorghum, and sugarcane (Anoop and Gupta, 2003; Su and Wu, 2004; Hmida-Sayari et al., 2005; Vendruscolo et al., 2007; Su et al., 2011; Guerzoni et al., 2014). Thus, overproduction of proline is beneficial to make transgenic plants suitable for agricultural use in saline soils. In our study, mir_205 led to up-regulation of its target P5CS isoform 1 (GRMZM2G375504) in leaves during salt treatment (**Figure 6**), which may be one reason of salt-tolerance in maize.

In this study, some novel miRNAs tested by qRT-PCR were *trans*-regulated in leaves, roots, or both under salt treatment, e.g., mir_250 vs. glutathione peroxidase (GPX) and DNA (cytosine-5-)-methyltransferase, mir_316 vs. gibberellin receptor (GID1), mir_330 vs. casein kinase II subunit alpha (CK2α) and cysteine proteinase inhibitor, and mir_316 vs. sucrose-phosphatase 1 (SPP1) (**Figure 6**). A recent study shows that CK2α subunits affect diverse developmental and stress responsive pathways in *Arabidopsis* such as photomorphogenesis, circadian rhythms, flowering time, lateral root development, cell cycle and cell division, cell expansion, auxin signaling, seed storage, SA responses, ABA responses, and NaCl responses (Mulekar et al., 2012). In addition, GPXs (EC 1.11.1.9) are key enzymes of the antioxidant network in plants. They provided protection from salinity stress during germination and seedling growth, and this protective effect appeared to allow transgenic plants to retain high levels of metabolic activity and growth (Zhai et al., 2013). Furthermore, it was found that GPXs, as a set of antioxidant enzymes, protect against oxidative stress, salt stress, and membrane damage (Matamoros et al., 2015). In our study, these two genes (casein kinase II subunit alpha – GRMZM2G046092 and GPX-GRMZM2G012479) were up-regulated in the roots under salt treatment, and GPX-GRMZM2G012479 was also up-regulated in the leaves. Casein kinase II subunit alpha was down-regulated in the leaves under salt stress, which may be due to the up-regulation of mir_330 (**Figure 6**). Thus, in this study, a possible internal regulation pathway was proposed for four species of maize novel miRNAs in response to salt stress. When maize is under salt stress, the expression of four novel miRNAs (mir-250, mir-205, mir-330, and mir-17) is down-regulated in the leaves or roots of maize, and their targets, such as casein kinase II, *GPX*, *P5CS*, *IF-1*, and some other genes, are up-regulated, and thereafter, some salt tolerant pathways are activated to adapt to salt stress (**Figure 7**).

## Author Contributions

RF, MZ, YZ, and XH performed most of the experiments. CD, SW, and YF helped in seedling planting and sample preparation. XS helped analyze the results. PL helped design the experiments and revise the manuscript. BW designed the experiments and edited the manuscript. All authors read and approved the final manuscript.

## Conflict of Interest Statement

The authors declare that the research was conducted in the absence of any commercial or financial relationships that could be construed as a potential conflict of interest.

## Footnotes

1 http://soap.genomics.org.cn/

2 http://ensembl.gramene.org/Zea_mays/Info/Index

3 http://www.simgene.com/Primer3

## Supplementary Material

**FIGURE S1 | Small RNA sequencing experimental process.**

**FIGURE S2 | Data analysis process.**

**FIGURE S3 | The workflow of the bioinformatics analysis.** The figure above only shows the workflow of a single sample. For multiple samples, there are additional analyses, such as common and specific tags/genes between samples.

**FIGURE S4 | Functions of genes via GO analysis among degradome sequencing.**
