# Supplementary figures and images for: Identification of Salt Tolerance-related microRNAs and Their Targets in Maize (Zea mays L.) Using High-throughput Sequencing and Degradome Analysis
Source: Front Plant Sci. 2017 May 26;8:864. doi: 10.3389/fpls.2017.00864 (PMC5445174; doi:10.3389/fpls.2017.00864)

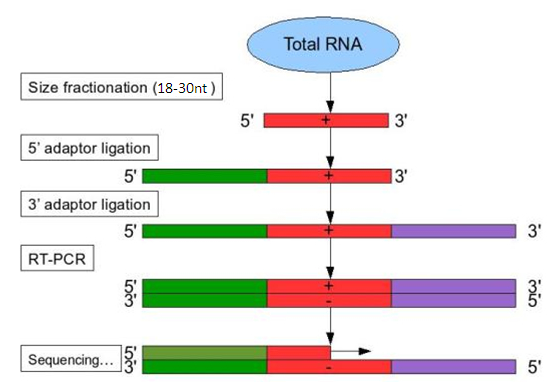

Supplement: Supplementary file 1 [file Image_1.PNG]

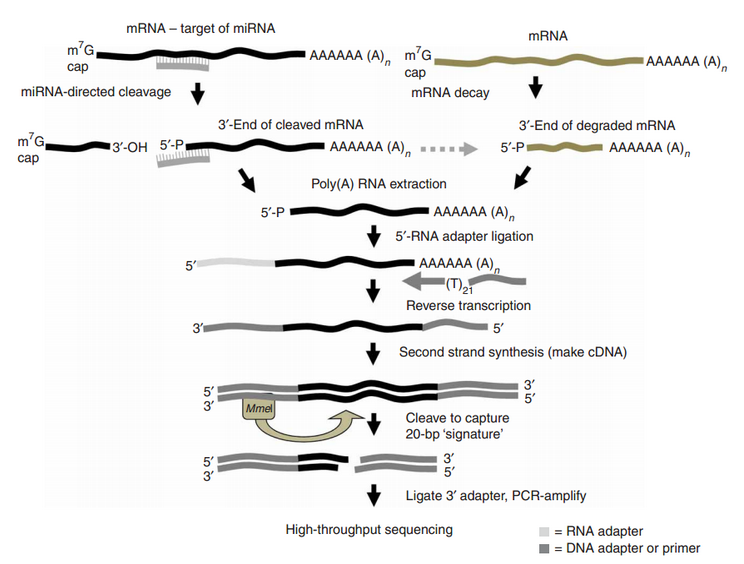

Supplement: Supplementary file 2 [file Image_2.PNG]

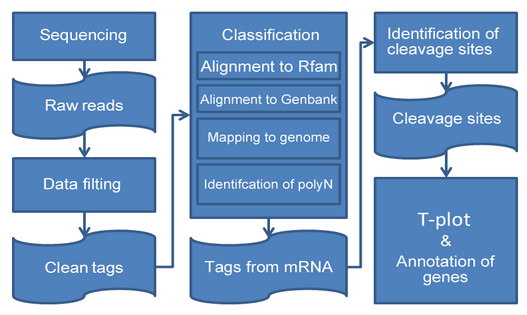

Supplement: Supplementary file 3 [file Image_3.PNG]

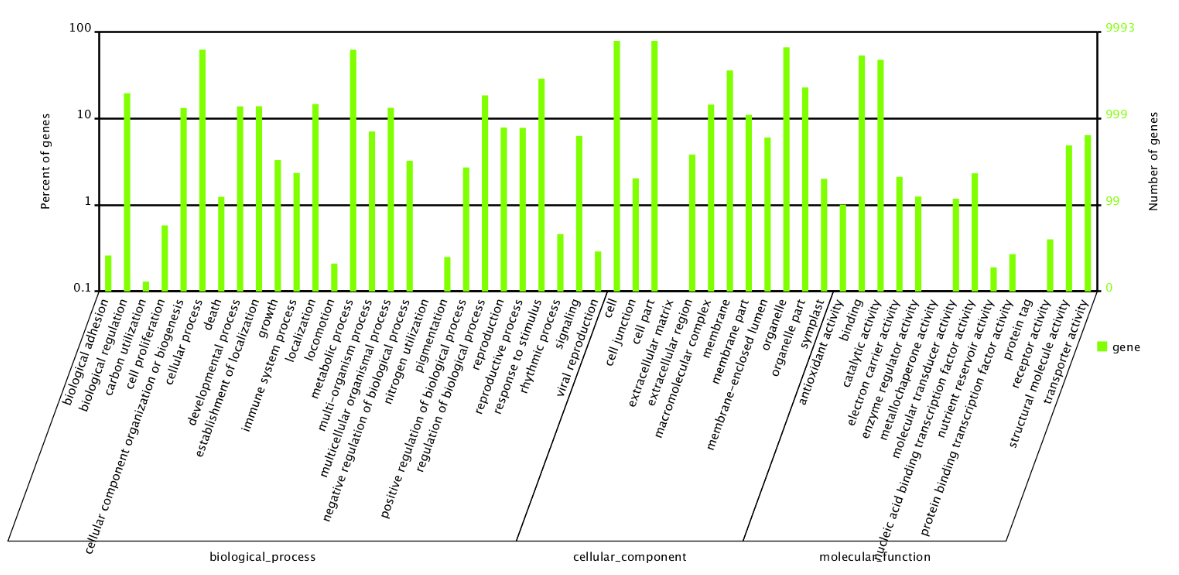

Supplement: Supplementary file 4 [file Image_4.PNG]
