# Supplementary material for: Identification of Salt Tolerance-related microRNAs and Their Targets in Maize (Zea mays L.) Using High-throughput Sequencing and Degradome Analysis
Source: Front Plant Sci. 2017 May 26;8:864. doi: 10.3389/fpls.2017.00864 (PMC5445174; doi:10.3389/fpls.2017.00864)
Supplement: Supplementary file 5 [file Table_1.DOC]

**Table S1 Summary of read counts.**

| **small RNA sequence** | | | | | | | | | | | | | |
| --- | --- | --- | --- | --- | --- | --- | --- | --- | --- | --- | --- | --- | --- |
| **Library** | **Raw reads** | **Clean reads** | | **Unique clean reads** | **Match Genome** | **miR** | | **rR** | **snR** | | **snoR** | **tR** | **other** |
| LC | 12067792 | 11906526(99.38%) | | 4093146 | 2617499 | 2313 | | 29076 | 2880 | | 1511 | 9551 | 4050128 |
| LS | 12579808 | 12368479(99.07%) | | 4556281 | 2815527 | 2266 | | 40648 | 2574 | | 1240 | 11083 | 4500736 |
| RC | 12044375 | 11752357(98.36%) | | 3907098 | 2084575 | 1834 | | 141975 | 3816 | | 1810 | 31887 | 3727610 |
| RS | 12885899 | 12675655(99.14%) | | 4162141 | 2022053 | 2089 | | 170657 | 4523 | | 2361 | 37930 | 3946670 |
| **degradome sequence** | | | | | | **unique count of tags** | | | | | | |  |
| **Library** | **Raw tags** | **Clean tags** | **Unique clean tags** | | **Match Genome** | **miR** | **rR** | | | **snR** | **snoR** | **tR** | **other** |
| LC | 12966755 | 12924324(99.67%) | 5528558 | | 3973925 | 3371 | 5025 | | | 3095 | 3380 | 1177 | 5515881 |
| LS | 12748707 | 12725088(99.81%) | 4678993 | | 3245055 | 2304 | 5607 | | | 1928 | 2062 | 1002 | 3588476 |
| RC | 12566032 | 12526023(99.68%) | 3599075 | | 2524305 | 1480 | 4669 | | | 2303 | 2492 | 1021 | 4668508 |
| RS | 12800651 | 12780073(99.84%) | 5787913 | | 3965739 | 2231 | 7909 | | | 4044 | 3506 | 2277 | 5770177 |
|  |  |  |  | |  |  |  | | |  |  |  |  |
