# Supplementary material for: Identification of Salt Tolerance-related microRNAs and Their Targets in Maize (Zea mays L.) Using High-throughput Sequencing and Degradome Analysis
Source: Front Plant Sci. 2017 May 26;8:864. doi: 10.3389/fpls.2017.00864 (PMC5445174; doi:10.3389/fpls.2017.00864)
Supplement: Supplementary file 6 [file Table_2.DOC]

**Table S2** The similarity of the four maize small RNA libraries: LC, LS, RC and RS; SP (similar percentage).

| **SP** | **LC** | **LS** | **RC** | **RS** |
| --- | --- | --- | --- | --- |
| **LC** | - | 59.73% | 32.89% | - |
| **LS** | 59.73% | - | - | 34.67% |
| **RC** | 32.89% | - | - | 42.33% |
| **RS** | - | 34.67% | 42.33% | - |
