# Supplementary material for: Identification of Salt Tolerance-related microRNAs and Their Targets in Maize (Zea mays L.) Using High-throughput Sequencing and Degradome Analysis
Source: Front Plant Sci. 2017 May 26;8:864. doi: 10.3389/fpls.2017.00864 (PMC5445174; doi:10.3389/fpls.2017.00864)
Supplement: Supplementary file 7 [file Table_3.DOC]

**Table S3** The Summary of the comparison of novel miRNAs between the control and salt libraries in maize.

| **pairwise** | **miR-name** | **LC-std** | **LS-std** | **fold-change (log_2_ LS/LC)** | **p-value** | **sig-lable** |
| --- | --- | --- | --- | --- | --- | --- |
| LC-LS | novel_mir_1 | 2.9396 | 1.4553 | -1.01430327 | 0.013554 | * |
| LC-LS | novel_mir_104 | 1.1758 | 0.7277 | -0.69222697 | 0.265073 |  |
| LC-LS | novel_mir_105 | 4.0314 | 4.2042 | 0.06055037 | 0.836767 |  |
| LC-LS | novel_mir_107 | 1.5958 | 1.1319 | -0.49553335 | 0.332811 |  |
| LC-LS | novel_mir_114 | 150.2537 | 92.8166 | -0.69494576 | 4.44E-38 |  |
| LC-LS | novel_mir_115 | 8.6507 | 2.7489 | -1.65396245 | 4.85E-10 | ** |
| LC-LS | novel_mir_132 | 8.6507 | 6.4681 | -0.41947489 | 0.050554 |  |
| LC-LS | novel_mir_137 | 1.5118 | 0.8085 | -0.90294762 | 0.111061 |  |
| LC-LS | novel_mir_148 | 4.9553 | 0.7277 | -2.76755669 | 8.35E-11 | ** |
| LC-LS | novel_mir_150 | 9.0707 | 0.01 | -9.82507014 | 3.80E-34 | ** |
| LC-LS | novel_mir_155 | 5.7951 | 5.417 | -0.09733947 | 0.693627 |  |
| LC-LS | novel_mir_157 | 6.0471 | 5.9021 | -0.03501507 | 0.882628 |  |
| LC-LS | novel_mir_158 | 7.2229 | 7.2766 | 0.01068631 | 0.962521 |  |
| LC-LS | novel_mir_161 | 1.2598 | 1.5362 | 0.28617134 | 0.574168 |  |
| LC-LS | novel_mir_164 | 2.3517 | 2.1021 | -0.16187273 | 0.681022 |  |
| LC-LS | novel_mir_17 | 1.7637 | 0.01 | -7.46246138 | 3.12E-07 | ** |
| LC-LS | novel_mir_173 | 3.1075 | 1.2936 | -1.26436281 | 0.002446 | ** |
| LC-LS | novel_mir_175 | 9.1546 | 0.01 | -9.83835306 | 1.86E-34 | ** |
| LC-LS | novel_mir_176 | 2.0157 | 1.6979 | -0.24752944 | 0.567724 |  |
| LC-LS | novel_mir_177 | 0.6719 | 1.4553 | 1.11499815 | 0.065393 |  |
| LC-LS | novel_mir_178 | 1.1758 | 0.8894 | -0.40273837 | 0.493406 |  |
| LC-LS | novel_mir_179 | 4.8713 | 0.01 | -8.92816303 | 1.12E-18 | ** |
| LC-LS | novel_mir_18 | 2.7716 | 2.6681 | -0.05490632 | 0.875746 |  |
| LC-LS | novel_mir_182 | 3.8634 | 2.7489 | -0.49101663 | 0.132085 |  |
| LC-LS | novel_mir_185 | 1.4278 | 0.01 | -7.15765011 | 5.40E-06 | ** |
| LC-LS | novel_mir_189 | 2.4356 | 0.01 | -7.92813341 | 1.05E-09 | ** |
| LC-LS | novel_mir_190 | 1.8477 | 0.01 | -7.52958672 | 1.53E-07 | ** |
| LC-LS | novel_mir_198 | 1.5118 | 0.01 | -7.24012348 | 2.65E-06 | ** |
| LC-LS | novel_mir_20 | 7.2229 | 7.9234 | 0.13354144 | 0.533236 |  |
| LC-LS | novel_mir_200 | 1.0079 | 0.01 | -6.6552087 | 0.00019 | ** |
| LC-LS | novel_mir_205 | 1.4278 | 0.01 | -7.15765011 | 5.40E-06 | ** |
| LC-LS | novel_mir_212 | 4.6193 | 3.0723 | -0.58835515 | 0.052232 |  |
| LC-LS | novel_mir_214 | 20.493 | 15.6042 | -0.3931968 | 0.004549 |  |
| LC-LS | novel_mir_215 | 1.1758 | 0.01 | -6.87749887 | 4.58E-05 | ** |
| LC-LS | novel_mir_217 | 1.2598 | 0.4043 | -1.63969661 | 0.021229 | * |
| LC-LS | novel_mir_220 | 1.3438 | 0.01 | -7.07017462 | 1.10E-05 | ** |
| LC-LS | novel_mir_221 | 2.9396 | 1.4553 | -1.01430327 | 0.013554 | * |
| LC-LS | novel_mir_235 | 26.5401 | 15.8467 | -0.74399137 | 8.85E-09 |  |
| LC-LS | novel_mir_236 | 21.1649 | 9.9446 | -1.08968842 | 1.39E-12 | ** |
| LC-LS | novel_mir_237 | 2.2677 | 1.5362 | -0.56186374 | 0.194112 |  |
| LC-LS | novel_mir_239 | 5.1232 | 2.8298 | -0.85634512 | 0.004502 |  |
| LC-LS | novel_mir_240 | 7.3069 | 0.01 | -9.51311569 | 1.19E-27 | ** |
| LC-LS | novel_mir_244 | 3.9474 | 2.7489 | -0.52204829 | 0.107464 |  |
| LC-LS | novel_mir_25 | 3.5275 | 2.183 | -0.69233395 | 0.050364 |  |
| LC-LS | novel_mir_250 | 13.186 | 0.01 | -10.36479127 | 2.63E-49 | ** |
| LC-LS | novel_mir_251 | 1.4278 | 0.566 | -1.33491995 | 0.034361 | * |
| LC-LS | novel_mir_255 | 4.6193 | 1.1319 | -2.02892774 | 1.89E-07 | ** |
| LC-LS | novel_mir_26 | 1.3438 | 1.3745 | 0.03258847 | 0.952718 |  |
| LC-LS | novel_mir_264 | 10.0785 | 6.7106 | -0.58676727 | 0.004129 |  |
| LC-LS | novel_mir_267 | 6.551 | 4.6893 | -0.48234257 | 0.05317 |  |
| LC-LS | novel_mir_273 | 13.27 | 9.8638 | -0.42795292 | 0.013555 |  |
| LC-LS | novel_mir_274 | 8.1468 | 5.3361 | -0.61044779 | 0.007581 |  |
| LC-LS | novel_mir_29 | 52.4922 | 49.8849 | -0.07349988 | 0.369174 |  |
| LC-LS | novel_mir_317 | 0.01 | 5.0127 | 8.96944408 | 7.11E-19 | ** |
| LC-LS | novel_mir_325 | 0.01 | 2.8298 | 8.14455628 | 5.74E-11 | ** |
| LC-LS | novel_mir_33 | 1.7637 | 1.2936 | -0.4472136 | 0.35321 |  |
| LC-LS | novel_mir_330 | 0.01 | 4.1234 | 8.68769061 | 1.18E-15 | ** |
| LC-LS | novel_mir_331 | 0.01 | 1.0511 | 6.71575612 | 0.000159 | ** |
| LC-LS | novel_mir_335 | 0.01 | 1.2936 | 7.01524777 | 2.10E-05 | ** |
| LC-LS | novel_mir_34 | 7.2229 | 5.4978 | -0.39372377 | 0.092231 |  |
| LC-LS | novel_mir_388 | 0.01 | 3.1532 | 8.30067287 | 3.87E-12 | ** |
| LC-LS | novel_mir_390 | 0.01 | 3.0723 | 8.26317529 | 7.59E-12 | ** |
| LC-LS | novel_mir_41 | 0.8399 | 2.7489 | 1.71056495 | 0.0004 | ** |
| LC-LS | novel_mir_448 | 0.01 | 3.8 | 8.56985561 | 1.76E-14 | ** |
| LC-LS | novel_mir_57 | 0.9239 | 2.6681 | 1.53000413 | 0.001286 | ** |
| LC-LS | novel_mir_61 | 4.7873 | 1.6979 | -1.49546072 | 1.80E-05 | ** |
| LC-LS | novel_mir_76 | 14.2779 | 12.8553 | -0.15142052 | 0.341397 |  |
| LC-LS | novel_mir_77 | 2.5196 | 1.4553 | -0.79187813 | 0.063842 |  |
| LC-LS | novel_mir_80 | 13.606 | 0.01 | -10.41002721 | 7.46E-51 | ** |
| LC-LS | novel_mir_85 | 1.2598 | 1.0511 | -0.26129478 | 0.635668 |  |
| LC-LS | novel_mir_9 | 12.8501 | 0.01 | -10.3275638 | 4.54E-48 | ** |
| LC-LS | novel_mir_90 | 0.5039 | 1.0511 | 1.06069057 | 0.136362 |  |
| LC-LS | novel_mir_91 | 21.7528 | 12.5319 | -0.79559595 | 3.44E-08 |  |
| LC-LS | novel_mir_92 | 29.8996 | 27.9743 | -0.09602415 | 0.377825 |  |
| LC-LS | novel_mir_93 | 3.1075 | 0.01 | -8.27961059 | 3.51E-12 | ** |
| LC-LS | novel_mir_95 | 2.2677 | 0.01 | -7.82508598 | 4.35E-09 | ** |
| LC-LS | novel_mir_100 | 4.2834 | 3.9617 | -0.11263677 | 0.696102 |  |
| LC-LS | novel_mir_102 | 0.9239 | 1.0511 | 0.18609132 | 0.76208 |  |
| LC-LS | novel_mir_103 | 1.1758 | 0.01 | -6.87749887 | 4.58E-05 | ** |
| LC-LS | novel_mir_106 | 1.7637 | 0.01 | -7.46246138 | 3.12E-07 | ** |
| LC-LS | novel_mir_109 | 2.0997 | 0.01 | -7.71403941 | 1.81E-08 | ** |
| LC-LS | novel_mir_11 | 1.0918 | 0.01 | -6.77056479 | 9.33E-05 | ** |
| LC-LS | novel_mir_112 | 0.8399 | 1.2128 | 0.53005219 | 0.377429 |  |
| LC-LS | novel_mir_113 | 1.3438 | 0.566 | -1.24744448 | 0.051664 |  |
| LC-LS | novel_mir_117 | 1.6798 | 1.3745 | -0.28938257 | 0.545939 |  |
| LC-LS | novel_mir_118 | 1.1758 | 0.01 | -6.87749887 | 4.58E-05 | ** |
| LC-LS | novel_mir_119 | 1.0918 | 0.8894 | -0.29580429 | 0.620685 |  |
| LC-LS | novel_mir_122 | 1.6798 | 0.4851 | -1.79193539 | 0.004379 | ** |
| LC-LS | novel_mir_123 | 1.1758 | 0.01 | -6.87749887 | 4.58E-05 | ** |
| LC-LS | novel_mir_126 | 1.0079 | 1.1319 | 0.167394 | 0.776357 |  |
| LC-LS | novel_mir_134 | 1.0918 | 0.01 | -6.77056479 | 9.33E-05 | ** |
| LC-LS | novel_mir_135 | 2.2677 | 0.8085 | -1.48791012 | 0.003535 | ** |
| LC-LS | novel_mir_138 | 2.2677 | 0.566 | -2.00235584 | 0.000335 | ** |
| LC-LS | novel_mir_139 | 1.2598 | 0.01 | -6.97705091 | 2.24E-05 | ** |
| LC-LS | novel_mir_140 | 2.0997 | 0.01 | -7.71403941 | 1.81E-08 | ** |
| LC-LS | novel_mir_144 | 1.0079 | 0.4043 | -1.3178544 | 0.081127 |  |
| LC-LS | novel_mir_145 | 1.0918 | 0.01 | -6.77056479 | 9.33E-05 | ** |
| LC-LS | novel_mir_149 | 1.0918 | 0.01 | -6.77056479 | 9.33E-05 | ** |
| LC-LS | novel_mir_151 | 0.8399 | 2.8298 | 1.75241062 | 0.000258 | ** |
| LC-LS | novel_mir_156 | 1.0079 | 0.01 | -6.6552087 | 0.00019 | ** |
| LC-LS | novel_mir_159 | 1.2598 | 0.8894 | -0.50229041 | 0.385197 |  |
| LC-LS | novel_mir_160 | 36.6186 | 24.4978 | -0.57992444 | 6.01E-08 |  |
| LC-LS | novel_mir_169 | 1.3438 | 0.01 | -7.07017462 | 1.10E-05 | ** |
| LC-LS | novel_mir_172 | 2.2677 | 0.01 | -7.82508598 | 4.35E-09 | ** |
| LC-LS | novel_mir_180 | 0.4199 | 1.8596 | 2.14687464 | 0.000787 | ** |
| LC-LS | novel_mir_183 | 2.1837 | 1.4553 | -0.58545808 | 0.186325 |  |
| LC-LS | novel_mir_184 | 6.0471 | 7.2766 | 0.26702107 | 0.243003 |  |
| LC-LS | novel_mir_186 | 1.2598 | 0.01 | -6.97705091 | 2.24E-05 | ** |
| LC-LS | novel_mir_19 | 1.9317 | 0.566 | -1.7709971 | 0.002364 | ** |
| LC-LS | novel_mir_192 | 1.1758 | 0.01 | -6.87749887 | 4.58E-05 | ** |
| LC-LS | novel_mir_193 | 1.8477 | 0.01 | -7.52958672 | 1.53E-07 | ** |
| LC-LS | novel_mir_195 | 1.4278 | 0.01 | -7.15765011 | 5.40E-06 | ** |
| LC-LS | novel_mir_196 | 2.8556 | 1.2936 | -1.14240232 | 0.00739 | ** |
| LC-LS | novel_mir_197 | 1.7637 | 0.4851 | -1.8622511 | 0.002698 | ** |
| LC-LS | novel_mir_199 | 1.6798 | 1.5362 | -0.12892342 | 0.780321 |  |
| LC-LS | novel_mir_201 | 1.0079 | 0.01 | -6.6552087 | 0.00019 | ** |
| LC-LS | novel_mir_202 | 3.0236 | 2.183 | -0.46995516 | 0.201251 |  |
| LC-LS | novel_mir_203 | 7.055 | 1.7787 | -1.98782288 | 1.86E-10 | ** |
| LC-LS | novel_mir_204 | 3.3595 | 0.01 | -8.39210273 | 4.14E-13 | ** |
| LC-LS | novel_mir_208 | 1.3438 | 0.01 | -7.07017462 | 1.10E-05 | ** |
| LC-LS | novel_mir_21 | 2.6876 | 0.4851 | -2.46996435 | 8.43E-06 | ** |
| LC-LS | novel_mir_210 | 1.2598 | 0.01 | -6.97705091 | 2.24E-05 | ** |
| LC-LS | novel_mir_22 | 1.0079 | 0.01 | -6.6552087 | 0.00019 | ** |
| LC-LS | novel_mir_225 | 1.0918 | 0.8894 | -0.29580429 | 0.620685 |  |
| LC-LS | novel_mir_226 | 1.6798 | 0.01 | -7.39214565 | 6.37E-07 | ** |
| LC-LS | novel_mir_227 | 4.3674 | 1.3745 | -1.66786776 | 9.41E-06 | ** |
| LC-LS | novel_mir_228 | 1.1758 | 0.6468 | -0.8622511 | 0.178546 |  |
| LC-LS | novel_mir_23 | 4.0314 | 4.3659 | 0.11499815 | 0.691123 |  |
| LC-LS | novel_mir_230 | 1.1758 | 7.1957 | 2.61349236 | 3.88E-14 | ** |
| LC-LS | novel_mir_231 | 1.0079 | 0.01 | -6.6552087 | 0.00019 | ** |
| LC-LS | novel_mir_232 | 3.6115 | 1.1319 | -1.67385166 | 5.53E-05 | ** |
| LC-LS | novel_mir_234 | 1.0079 | 0.8085 | -0.31803283 | 0.611515 |  |
| LC-LS | novel_mir_241 | 4.4513 | 4.7702 | 0.09982302 | 0.717763 |  |
| LC-LS | novel_mir_246 | 1.5958 | 0.01 | -7.31813605 | 1.30E-06 | ** |
| LC-LS | novel_mir_252 | 1.9317 | 0.6468 | -1.57847947 | 0.005058 | ** |
| LC-LS | novel_mir_254 | 2.8556 | 0.01 | -8.15765011 | 2.97E-11 | ** |
| LC-LS | novel_mir_258 | 15.3697 | 0.01 | -10.58587326 | 2.38E-57 | ** |
| LC-LS | novel_mir_261 | 1.0079 | 0.01 | -6.6552087 | 0.00019 | ** |
| LC-LS | novel_mir_262 | 1.5118 | 0.01 | -7.24012348 | 2.65E-06 | ** |
| LC-LS | novel_mir_263 | 1.0079 | 0.4851 | -1.05499842 | 0.142897 |  |
| LC-LS | novel_mir_27 | 1.5118 | 2.2638 | 0.58247921 | 0.183217 |  |
| LC-LS | novel_mir_270 | 2.5196 | 1.9404 | -0.37684063 | 0.341983 |  |
| LC-LS | novel_mir_271 | 1.3438 | 1.4553 | 0.11499815 | 0.822791 |  |
| LC-LS | novel_mir_276 | 1.5118 | 0.01 | -7.24012348 | 2.65E-06 | ** |
| LC-LS | novel_mir_279 | 0.01 | 1.8596 | 7.53884852 | 1.87E-07 | ** |
| LC-LS | novel_mir_28 | 1.4278 | 0.01 | -7.15765011 | 5.40E-06 | ** |
| LC-LS | novel_mir_280 | 0.01 | 1.2936 | 7.01524777 | 2.10E-05 | ** |
| LC-LS | novel_mir_285 | 0.01 | 4.2042 | 8.71568749 | 6.03E-16 | ** |
| LC-LS | novel_mir_286 | 0.01 | 1.4553 | 7.18517278 | 5.46E-06 | ** |
| LC-LS | novel_mir_289 | 0.01 | 7.3574 | 9.52305222 | 2.29E-27 | ** |
| LC-LS | novel_mir_290 | 0.01 | 1.7787 | 7.47467939 | 3.68E-07 | ** |
| LC-LS | novel_mir_298 | 0.01 | 1.0511 | 6.71575612 | 0.000159 | ** |
| LC-LS | novel_mir_32 | 12.9341 | 12.0468 | -0.1025297 | 0.536048 |  |
| LC-LS | novel_mir_332 | 0.01 | 1.4553 | 7.18517278 | 5.46E-06 | ** |
| LC-LS | novel_mir_337 | 0.01 | 1.617 | 7.33717587 | 1.42E-06 | ** |
| LC-LS | novel_mir_340 | 0.01 | 3.8 | 8.56985561 | 1.76E-14 | ** |
| LC-LS | novel_mir_341 | 0.01 | 1.3745 | 7.1027631 | 1.07E-05 | ** |
| LC-LS | novel_mir_342 | 0.01 | 2.4255 | 7.92213837 | 1.67E-09 | ** |
| LC-LS | novel_mir_343 | 0.01 | 8.3276 | 9.70175696 | 7.00E-31 | ** |
| LC-LS | novel_mir_344 | 0.01 | 1.2128 | 6.92219785 | 4.13E-05 | ** |
| LC-LS | novel_mir_348 | 0.01 | 1.1319 | 6.8226027 | 8.10E-05 | ** |
| LC-LS | novel_mir_350 | 0.01 | 1.2936 | 7.01524777 | 2.10E-05 | ** |
| LC-LS | novel_mir_363 | 0.01 | 4.6085 | 8.84815344 | 2.07E-17 | ** |
| LC-LS | novel_mir_367 | 0.01 | 1.7787 | 7.47467939 | 3.68E-07 | ** |
| LC-LS | novel_mir_37 | 1.6798 | 4.0425 | 1.2669583 | 0.000552 | ** |
| LC-LS | novel_mir_371 | 0.01 | 3.3149 | 8.37282154 | 1.00E-12 | ** |
| LC-LS | novel_mir_376 | 0.01 | 1.7787 | 7.47467939 | 3.68E-07 | ** |
| LC-LS | novel_mir_398 | 0.01 | 1.6979 | 7.40760768 | 7.22E-07 | ** |
| LC-LS | novel_mir_4 | 2.5196 | 2.0213 | -0.31791125 | 0.417618 |  |
| LC-LS | novel_mir_411 | 0.01 | 1.0511 | 6.71575612 | 0.000159 | ** |
| LC-LS | novel_mir_417 | 0.01 | 1.9404 | 7.60021027 | 9.55E-08 | ** |
| LC-LS | novel_mir_420 | 0.01 | 2.183 | 7.77016832 | 1.26E-08 | ** |
| LC-LS | novel_mir_422 | 0.01 | 1.4553 | 7.18517278 | 5.46E-06 | ** |
| LC-LS | novel_mir_424 | 0.01 | 1.1319 | 6.8226027 | 8.10E-05 | ** |
| LC-LS | novel_mir_44 | 11.3383 | 0.01 | -10.14698868 | 1.68E-42 | ** |
| LC-LS | novel_mir_441 | 0.01 | 1.4553 | 7.18517278 | 5.46E-06 | ** |
| LC-LS | novel_mir_45 | 2.4356 | 0.4043 | -2.59077911 | 1.37E-05 | ** |
| LC-LS | novel_mir_451 | 0.01 | 1.0511 | 6.71575612 | 0.000159 | ** |
| LC-LS | novel_mir_454 | 0.01 | 1.9404 | 7.60021027 | 9.55E-08 | ** |
| LC-LS | novel_mir_455 | 0.01 | 4.0425 | 8.65910396 | 2.32E-15 | ** |
| LC-LS | novel_mir_47 | 2.5196 | 2.0213 | -0.31791125 | 0.417618 |  |
| LC-LS | novel_mir_48 | 1.0079 | 0.01 | -6.6552087 | 0.00019 | ** |
| LC-LS | novel_mir_52 | 2.0157 | 0.01 | -7.65513713 | 3.69E-08 | ** |
| LC-LS | novel_mir_53 | 1.4278 | 0.01 | -7.15765011 | 5.40E-06 | ** |
| LC-LS | novel_mir_56 | 3.3595 | 1.3745 | -1.28933962 | 0.001369 | ** |
| LC-LS | novel_mir_59 | 1.5118 | 0.4851 | -1.63991321 | 0.011226 | * |
| LC-LS | novel_mir_6 | 1.0079 | 0.01 | -6.6552087 | 0.00019 | ** |
| LC-LS | novel_mir_60 | 3.0236 | 2.5872 | -0.22487571 | 0.522356 |  |
| LC-LS | novel_mir_68 | 1.5118 | 0.7277 | -1.05485158 | 0.070085 |  |
| LC-LS | novel_mir_69 | 1.0079 | 0.01 | -6.6552087 | 0.00019 | ** |
| LC-LS | novel_mir_70 | 1.0918 | 0.8085 | -0.43338892 | 0.480795 |  |
| LC-LS | novel_mir_72 | 6.635 | 0.01 | -9.37395266 | 3.56E-25 | ** |
| LC-LS | novel_mir_73 | 4.6193 | 0.9702 | -2.25132016 | 2.63E-08 | ** |
| LC-LS | novel_mir_75 | 3.1915 | 2.9915 | -0.09336558 | 0.778677 |  |
| LC-LS | novel_mir_8 | 13.69 | 7.2766 | -0.91178603 | 8.97E-07 |  |
| LC-LS | novel_mir_81 | 1.3438 | 1.5362 | 0.19304762 | 0.700845 |  |
| LC-LS | novel_mir_82 | 1.1758 | 0.01 | -6.87749887 | 4.58E-05 | ** |
| LC-LS | novel_mir_83 | 0.7559 | 1.3745 | 0.86263961 | 0.147876 |  |
| LC-LS | novel_mir_84 | 1.0918 | 0.6468 | -0.75531702 | 0.247576 |  |
| LC-LS | novel_mir_94 | 0.9239 | 1.5362 | 0.73355744 | 0.18197 |  |
| LC-LS | novel_mir_36 | 168.395 | 433.1171 | 1.36290783 | 3.74778436309336e-319 | ** |
| RC-RS | novel_mir_1 | 0.01 | 2.0512 | 7.68032436 | 4.06E-08 | ** |
| RC-RS | novel_mir_104 | 1.3614 | 1.3412 | -0.02156663 | 0.959336 |  |
| RC-RS | novel_mir_105 | 4.3396 | 3.0768 | -0.4961314 | 0.105252 |  |
| RC-RS | novel_mir_107 | 0.5105 | 1.1045 | 1.11341055 | 0.112439 |  |
| RC-RS | novel_mir_114 | 0.01 | 1.7356 | 7.43929068 | 5.60E-07 | ** |
| RC-RS | novel_mir_115 | 6.8922 | 16.015 | 1.21638733 | 1.88E-11 | ** |
| RC-RS | novel_mir_132 | 0.01 | 8.2047 | 9.68030677 | 2.42E-30 | ** |
| RC-RS | novel_mir_137 | 1.5316 | 0.01 | -7.25889575 | 1.83E-06 | ** |
| RC-RS | novel_mir_148 | 1.0211 | 0.01 | -6.67398035 | 0.000148 | ** |
| RC-RS | novel_mir_150 | 1.0211 | 0.01 | -6.67398035 | 0.000148 | ** |
| RC-RS | novel_mir_155 | 0.01 | 6.0746 | 9.2466456 | 1.20E-22 | ** |
| RC-RS | novel_mir_157 | 0.6807 | 2.3667 | 1.79778583 | 0.000699 | ** |
| RC-RS | novel_mir_158 | 12.1678 | 0.01 | -10.2488526 | 3.49E-46 | ** |
| RC-RS | novel_mir_161 | 0.936 | 1.1045 | 0.23881298 | 0.694591 |  |
| RC-RS | novel_mir_164 | 2.9781 | 0.01 | -8.21824839 | 7.27E-12 | ** |
| RC-RS | novel_mir_17 | 1.3614 | 0.01 | -7.0889472 | 7.93E-06 | ** |
| RC-RS | novel_mir_173 | 0.8509 | 1.6567 | 0.96125088 | 0.080827 |  |
| RC-RS | novel_mir_175 | 3.7439 | 1.7356 | -1.10910741 | 0.002549 | ** |
| RC-RS | novel_mir_176 | 0.8509 | 1.3412 | 0.65646289 | 0.260567 |  |
| RC-RS | novel_mir_177 | 1.7018 | 0.01 | -7.41091768 | 4.25E-07 | ** |
| RC-RS | novel_mir_178 | 0.01 | 1.42 | 7.14974712 | 7.72E-06 | ** |
| RC-RS | novel_mir_179 | 0.01 | 1.6567 | 7.37216857 | 1.08E-06 | ** |
| RC-RS | novel_mir_18 | 2.3825 | 3.629 | 0.60709584 | 0.078482 |  |
| RC-RS | novel_mir_182 | 1.2763 | 2.4456 | 0.93822098 | 0.036151 |  |
| RC-RS | novel_mir_185 | 1.3614 | 1.3412 | -0.02156663 | 0.959336 |  |
| RC-RS | novel_mir_189 | 0.01 | 1.3412 | 7.06738058 | 1.49E-05 | ** |
| RC-RS | novel_mir_190 | 1.1062 | 1.1045 | -0.00221883 | 0.989299 |  |
| RC-RS | novel_mir_198 | 3.5738 | 2.8401 | -0.33151717 | 0.311681 |  |
| RC-RS | novel_mir_20 | 5.0203 | 7.7314 | 0.6229561 | 0.008196 |  |
| RC-RS | novel_mir_200 | 0.01 | 1.0256 | 6.68032436 | 0.000205 | ** |
| RC-RS | novel_mir_205 | 0.01 | 1.2623 | 6.97991101 | 2.87E-05 | ** |
| RC-RS | novel_mir_212 | 2.5527 | 0.7889 | -1.69410966 | 0.000619 | ** |
| RC-RS | novel_mir_214 | 6.3817 | 5.2068 | -0.2935438 | 0.227678 |  |
| RC-RS | novel_mir_215 | 0.8509 | 1.1045 | 0.37633192 | 0.542903 |  |
| RC-RS | novel_mir_217 | 0.01 | 1.3412 | 7.06738058 | 1.49E-05 | ** |
| RC-RS | novel_mir_220 | 0.5105 | 2.0512 | 2.0064853 | 0.000714 | ** |
| RC-RS | novel_mir_221 | 0.01 | 1.4989 | 7.22776033 | 4.01E-06 | ** |
| RC-RS | novel_mir_235 | 11.1467 | 18.7762 | 0.75228845 | 1.06E-06 |  |
| RC-RS | novel_mir_236 | 3.7439 | 3.629 | -0.04496984 | 0.879054 |  |
| RC-RS | novel_mir_237 | 2.6378 | 1.0256 | -1.36286702 | 0.002999 | ** |
| RC-RS | novel_mir_239 | 0.01 | 1.1834 | 6.88679399 | 5.53E-05 | ** |
| RC-RS | novel_mir_240 | 0.936 | 1.1045 | 0.23881298 | 0.694591 |  |
| RC-RS | novel_mir_244 | 1.7018 | 1.6567 | -0.03874912 | 0.926319 |  |
| RC-RS | novel_mir_25 | 4.3396 | 4.2601 | -0.02667477 | 0.921166 |  |
| RC-RS | novel_mir_250 | 3.4036 | 0.01 | -8.41091768 | 1.87E-13 | ** |
| RC-RS | novel_mir_251 | 0.01 | 3.7079 | 8.53445853 | 4.22E-14 | ** |
| RC-RS | novel_mir_255 | 1.4465 | 1.9723 | 0.44731269 | 0.33077 |  |
| RC-RS | novel_mir_26 | 2.6378 | 1.5778 | -0.74142084 | 0.071578 |  |
| RC-RS | novel_mir_264 | 3.829 | 5.5224 | 0.52832773 | 0.054557 |  |
| RC-RS | novel_mir_267 | 4.8501 | 5.2857 | 0.12408005 | 0.637601 |  |
| RC-RS | novel_mir_273 | 8.4238 | 18.6184 | 1.14418601 | 5.21E-12 | ** |
| RC-RS | novel_mir_274 | 7.5729 | 8.0469 | 0.08758723 | 0.678978 |  |
| RC-RS | novel_mir_29 | 7.8282 | 25.1664 | 1.68474634 | 5.59E-27 | ** |
| RC-RS | novel_mir_317 | 0.8509 | 1.42 | 0.73882943 | 0.198171 |  |
| RC-RS | novel_mir_325 | 4.5097 | 1.1045 | -2.02963805 | 2.28E-07 | ** |
| RC-RS | novel_mir_33 | 1.6167 | 0.01 | -7.33690818 | 8.83E-07 | ** |
| RC-RS | novel_mir_330 | 4.0843 | 0.01 | -8.67394501 | 5.38E-16 | ** |
| RC-RS | novel_mir_331 | 0.7658 | 1.1045 | 0.52835385 | 0.404174 |  |
| RC-RS | novel_mir_335 | 1.1913 | 0.01 | -6.89639296 | 3.42E-05 | ** |
| RC-RS | novel_mir_34 | 0.7658 | 1.7356 | 1.18039493 | 0.034605 | * |
| RC-RS | novel_mir_388 | 0.01 | 1.42 | 7.14974712 | 7.72E-06 | ** |
| RC-RS | novel_mir_390 | 1.9571 | 0.01 | -7.61257367 | 4.73E-08 | ** |
| RC-RS | novel_mir_41 | 1.0211 | 0.01 | -6.67398035 | 0.000148 | ** |
| RC-RS | novel_mir_448 | 0.01 | 1.4989 | 7.22776033 | 4.01E-06 | ** |
| RC-RS | novel_mir_57 | 1.4465 | 1.3412 | -0.10904193 | 0.821752 |  |
| RC-RS | novel_mir_61 | 0.01 | 2.9979 | 8.22780845 | 1.55E-11 | ** |
| RC-RS | novel_mir_76 | 2.2974 | 5.6802 | 1.30593966 | 2.60E-05 | ** |
| RC-RS | novel_mir_77 | 1.1913 | 2.209 | 0.89085665 | 0.057 |  |
| RC-RS | novel_mir_80 | 2.5527 | 2.6034 | 0.02837299 | 0.943395 |  |
| RC-RS | novel_mir_85 | 0.01 | 2.6034 | 8.02425318 | 4.11E-10 | ** |
| RC-RS | novel_mir_9 | 4.8501 | 3.9446 | -0.29813548 | 0.285939 |  |
| RC-RS | novel_mir_90 | 0.01 | 1.2623 | 6.97991101 | 2.87E-05 | ** |
| RC-RS | novel_mir_91 | 5.1054 | 1.9723 | -1.37214499 | 3.01E-05 | ** |
| RC-RS | novel_mir_92 | 10.7213 | 14.5949 | 0.44498448 | 0.007288 |  |
| RC-RS | novel_mir_93 | 3.1483 | 2.9979 | -0.07062076 | 0.828903 |  |
| RC-RS | novel_mir_95 | 0.01 | 1.1045 | 6.78724961 | 0.000106 | ** |
| RC-RS | novel_mir_121 | 0.01 | 1.6567 | 7.37216857 | 1.08E-06 | ** |
| RC-RS | novel_mir_128 | 1.0211 | 1.5778 | 0.62779018 | 0.2387 |  |
| RC-RS | novel_mir_13 | 1.0211 | 0.01 | -6.67398035 | 0.000148 | ** |
| RC-RS | novel_mir_133 | 30.2918 | 23.5885 | -0.36084363 | 0.001398 |  |
| RC-RS | novel_mir_165 | 0.936 | 1.2623 | 0.43147439 | 0.457333 |  |
| RC-RS | novel_mir_349 | 1.1913 | 0.01 | -6.89639296 | 3.42E-05 | ** |
| RC-RS | novel_mir_355 | 0.01 | 1.0256 | 6.68032436 | 0.000205 | ** |
| RC-RS | novel_mir_396 | 0.01 | 1.4989 | 7.22776033 | 4.01E-06 | ** |
| RC-RS | novel_mir_421 | 0.7658 | 1.0256 | 0.4214286 | 0.515571 |  |
| RC-RS | novel_mir_431 | 0.01 | 1.42 | 7.14974712 | 7.72E-06 | ** |
| RC-RS | novel_mir_460 | 2.0421 | 0.01 | -7.67390972 | 2.27E-08 | ** |
| RC-RS | novel_mir_461 | 14.6354 | 21.4585 | 0.55208706 | 7.32E-05 |  |
| RC-RS | novel_mir_463 | 1.5316 | 0.01 | -7.25889575 | 1.83E-06 | ** |
| RC-RS | novel_mir_466 | 0.6807 | 1.0256 | 0.59137715 | 0.37463 |  |
| RC-RS | novel_mir_467 | 1.1913 | 0.4733 | -1.33170994 | 0.053147 |  |
| RC-RS | novel_mir_471 | 0.5956 | 1.1834 | 0.99052214 | 0.134183 |  |
| RC-RS | novel_mir_472 | 2.0421 | 0.01 | -7.67390972 | 2.27E-08 | ** |
| RC-RS | novel_mir_474 | 1.0211 | 0.01 | -6.67398035 | 0.000148 | ** |
| RC-RS | novel_mir_476 | 6.1264 | 0.01 | -9.25889573 | 1.27E-23 | ** |
| RC-RS | novel_mir_482 | 0.5956 | 1.1045 | 0.89097776 | 0.186302 |  |
| RC-RS | novel_mir_486 | 1.2763 | 0.01 | -6.99582367 | 1.65E-05 | ** |
| RC-RS | novel_mir_489 | 2.5527 | 2.5245 | -0.01602632 | 0.960448 |  |
| RC-RS | novel_mir_491 | 1.0211 | 0.01 | -6.67398035 | 0.000148 | ** |
| RC-RS | novel_mir_500 | 1.4465 | 0.01 | -7.1764225 | 3.81E-06 | ** |
| RC-RS | novel_mir_504 | 3.829 | 4.2601 | 0.15391964 | 0.602255 |  |
| RC-RS | novel_mir_506 | 1.5316 | 0.01 | -7.25889575 | 1.83E-06 | ** |
| RC-RS | novel_mir_509 | 1.1062 | 0.01 | -6.78946844 | 7.12E-05 | ** |
| RC-RS | novel_mir_512 | 1.3614 | 2.0512 | 0.59137715 | 0.200257 |  |
| RC-RS | novel_mir_513 | 1.4465 | 0.5522 | -1.38930353 | 0.027137 | * |
| RC-RS | novel_mir_514 | 2.2974 | 0.01 | -7.84385825 | 2.53E-09 | ** |
| RC-RS | novel_mir_518 | 0.7658 | 1.0256 | 0.4214286 | 0.515571 |  |
| RC-RS | novel_mir_526 | 1.1913 | 0.01 | -6.89639296 | 3.42E-05 | ** |
| RC-RS | novel_mir_527 | 1.2763 | 0.01 | -6.99582367 | 1.65E-05 | ** |
| RC-RS | novel_mir_529 | 1.7018 | 0.01 | -7.41091768 | 4.25E-07 | ** |
| RC-RS | novel_mir_531 | 1.1062 | 0.01 | -6.78946844 | 7.12E-05 | ** |
| RC-RS | novel_mir_533 | 4.0843 | 5.9957 | 0.55383936 | 0.036451 |  |
| RC-RS | novel_mir_534 | 2.2974 | 0.01 | -7.84385825 | 2.53E-09 | ** |
| RC-RS | novel_mir_538 | 0.7658 | 2.5245 | 1.72095811 | 0.00067 | ** |
| RC-RS | novel_mir_540 | 1.0211 | 0.01 | -6.67398035 | 0.000148 | ** |
| RC-RS | novel_mir_544 | 1.4465 | 0.01 | -7.1764225 | 3.81E-06 | ** |
| RC-RS | novel_mir_545 | 1.0211 | 0.01 | -6.67398035 | 0.000148 | ** |
| RC-RS | novel_mir_549 | 3.1483 | 0.01 | -8.2984292 | 1.68E-12 | ** |
| RC-RS | novel_mir_552 | 5.5308 | 10.9659 | 0.98746414 | 2.73E-06 |  |
| RC-RS | novel_mir_553 | 1.7018 | 0.7889 | -1.10914716 | 0.04363 | * |
| RC-RS | novel_mir_557 | 1.2763 | 0.01 | -6.99582367 | 1.65E-05 | ** |
| RC-RS | novel_mir_560 | 1.872 | 0.01 | -7.54843664 | 9.83E-08 | ** |
| RC-RS | novel_mir_561 | 1.1913 | 0.01 | -6.89639296 | 3.42E-05 | ** |
| RC-RS | novel_mir_564 | 1.7018 | 0.01 | -7.41091768 | 4.25E-07 | ** |
| RC-RS | novel_mir_565 | 1.6167 | 0.01 | -7.33690818 | 8.83E-07 | ** |
| RC-RS | novel_mir_567 | 1.0211 | 0.8678 | -0.23468967 | 0.696114 |  |
| RC-RS | novel_mir_569 | 0.01 | 1.1834 | 6.88679399 | 5.53E-05 | ** |
| RC-RS | novel_mir_573 | 0.01 | 3.3923 | 8.40611995 | 5.82E-13 | ** |
| RC-RS | novel_mir_575 | 0.01 | 5.838 | 9.1893304 | 8.55E-22 | ** |
| RC-RS | novel_mir_576 | 0.01 | 1.1045 | 6.78724961 | 0.000106 | ** |
| RC-RS | novel_mir_580 | 0.01 | 1.3412 | 7.06738058 | 1.49E-05 | ** |
| RC-RS | novel_mir_584 | 0.01 | 1.7356 | 7.43929068 | 5.60E-07 | ** |
| RC-RS | novel_mir_591 | 0.01 | 2.1301 | 7.73477735 | 2.11E-08 | ** |
| RC-RS | novel_mir_605 | 0.01 | 1.3412 | 7.06738058 | 1.49E-05 | ** |
| RC-RS | novel_mir_609 | 0.01 | 1.0256 | 6.68032436 | 0.000205 | ** |
| RC-RS | novel_mir_611 | 0.01 | 1.3412 | 7.06738058 | 1.49E-05 | ** |
| RC-RS | novel_mir_612 | 0.01 | 2.9979 | 8.22780845 | 1.55E-11 | ** |
| RC-RS | novel_mir_614 | 0.01 | 1.6567 | 7.37216857 | 1.08E-06 | ** |
| RC-RS | novel_mir_616 | 0.01 | 1.3412 | 7.06738058 | 1.49E-05 | ** |
| RC-RS | novel_mir_618 | 0.01 | 1.3412 | 7.06738058 | 1.49E-05 | ** |
| RC-RS | novel_mir_619 | 0.01 | 1.6567 | 7.37216857 | 1.08E-06 | ** |
| RC-RS | novel_mir_624 | 0.01 | 3.2345 | 8.3373989 | 2.16E-12 | ** |
| RC-RS | novel_mir_629 | 0.01 | 5.1279 | 9.00222432 | 3.14E-19 | ** |
| RC-RS | novel_mir_635 | 0.01 | 1.1834 | 6.88679399 | 5.53E-05 | ** |
| RC-RS | novel_mir_636 | 0.01 | 1.42 | 7.14974712 | 7.72E-06 | ** |
| RC-RS | novel_mir_637 | 0.01 | 1.1045 | 6.78724961 | 0.000106 | ** |
| RC-RS | novel_mir_639 | 0.01 | 2.3667 | 7.88673303 | 2.94E-09 | ** |
| RC-RS | novel_mir_645 | 0.01 | 1.42 | 7.14974712 | 7.72E-06 | ** |
| RC-RS | novel_mir_652 | 0.01 | 1.1045 | 6.78724961 | 0.000106 | ** |
| RC-RS | novel_mir_655 | 0.01 | 1.0256 | 6.68032436 | 0.000205 | ** |
| RC-RS | novel_mir_67 | 0.8509 | 1.42 | 0.73882943 | 0.198171 |  |
| RC-RS | novel_mir_87 | 1.1913 | 0.7889 | -0.59462242 | 0.322403 |  |
| RC-RS | novel_mir_316 | 1.6167 | 3.4712 | 1.1023825 | 0.00419 | ** |
