# Supplementary material for: Identification of Salt Tolerance-related microRNAs and Their Targets in Maize (Zea mays L.) Using High-throughput Sequencing and Degradome Analysis
Source: Front Plant Sci. 2017 May 26;8:864. doi: 10.3389/fpls.2017.00864 (PMC5445174; doi:10.3389/fpls.2017.00864)
Supplement: Supplementary file 8 [file Table_4.DOC]

**Table S4** A summary of the comparison of known miRNAs between the control and salt libraries in maize.

| **pairwise** | | **miR-name** | | **LC-std** | **LS-std** | | **fold-change (log_1.5_ LS/LC)** | | | **p-value** | **sig-lable** | | |
| --- | --- | --- | --- | --- | --- | --- | --- | --- | --- | --- | --- | --- | --- |
| LC-LS | | let-7 | | 14.5298 | 9.3787 | | -1.079650369 | | | 0.0002327 | ** | | |
| LC-LS | | let-7-5p | | 148.9939 | 62.4976 | | -2.142668237 | | | 2.35E-98 | ** | | |
| LC-LS | | let-7b-5p | | 36.4506 | 24.2552 | | -1.004591729 | | | 4.48E-08 | ** | | |
| LC-LS | | let-7c-5p | | 2.6876 | 0.01 | | -13.79605464 | | | 1.24E-10 | ** | | |
| LC-LS | | let-7d-5p | | 8.1468 | 5.2553 | | -1.081198144 | | | 0.0058525 | ** | | |
| LC-LS | | let-7f | | 246.5875 | 105.7527 | | -2.088005929 | | | 3.80E-155 | ** | | |
| LC-LS | | let-7f-5p | | 257.8418 | 107.774 | | -2.151380624 | | | 7.46E-170 | ** | | |
| LC-LS | | let-7g | | 2.2677 | 0.01 | | -13.37707284 | | | 4.35E-09 | ** | | |
| LC-LS | | let-7g-5p | | 13.27 | 8.3276 | | -1.149126122 | | | 0.0002018 | ** | | |
| LC-LS | | miR-1003-5p | | 0.01 | 7.5191 | | 16.33338234 | | | 5.94E-28 | ** | | |
| LC-LS | | miR1114 | | 0.01 | 7.034 | | 16.16890229 | | | 3.40E-26 | ** | | |
| LC-LS | | miR-11-3p | | 0.01 | 6.2255 | | 15.86776227 | | | 2.88E-23 | ** | | |
| LC-LS | | miR1160.2 | | 332.7587 | 64.9231 | | -4.030467914 | | | 0 | ** | | |
| LC-LS | | miR-1175-5p | | 13.354 | 0.01 | | -17.74995186 | | | 6.33E-50 | ** | | |
| LC-LS | | miR-1207-5p | | 70.8855 | 3.4766 | | -7.435932294 | | | 7.26E-201 | ** | | |
| LC-LS | | miR-1224-5p | | 23.5165 | 0.01 | | -19.14559957 | | | 2.33E-87 | ** | | |
| LC-LS | | miR-1228 | | 2.6876 | 0.01 | | -13.79605464 | | | 1.24E-10 | ** | | |
| LC-LS | | miR-1228-5p | | 0.01 | 3.234 | | 14.25249629 | | | 1.97E-12 | ** | | |
| LC-LS | | miR-1236-5p | | 0.01 | 8.9744 | | 16.7697445 | | | 3.18E-33 | ** | | |
| LC-LS | | miR-1253 | | 8.2308 | 4.9319 | | -1.263139277 | | | 0.0014883 | ** | | |
| LC-LS | | miR-1260a | | 7.1389 | 25.0637 | | 3.097336509 | | | 1.58E-29 | ** | | |
| LC-LS | | miR-1271b | | 5.5432 | 1.617 | | -3.038484309 | | | 1.71E-07 | ** | | |
| LC-LS | | miR-1290 | | 0.01 | 122.2462 | | 23.21089298 | | | 0 | ** | | |
| LC-LS | | miR-1292 | | 6.887 | 0.01 | | -16.11681413 | | | 4.20E-26 | ** | | |
| LC-LS | | miR-1299 | | 54.3399 | 34.0381 | | -1.153683183 | | | 4.09E-14 | ** | | |
| LC-LS | | miR-1306 | | 17.0495 | 0.01 | | -18.35248177 | | | 1.54E-63 | ** | | |
| LC-LS | | miR-1307-3p | | 0.01 | 4.4468 | | 15.03792756 | | | 7.98E-17 | ** | | |
| LC-LS | | miR-1333 | | 1.5118 | 0.01 | | -12.37707284 | | | 2.65E-06 | ** | | |
| LC-LS | | miR-1337 | | 11.8422 | 0.01 | | -17.453634 | | | 2.34E-44 | ** | | |
| LC-LS | | miR-1342 | | 7.4749 | 0.01 | | -16.3188418 | | | 2.87E-28 | ** | | |
| LC-LS | | miR-1371 | | 68.1139 | 0.01 | | -21.76846144 | | | 1.23E-251 | ** | | |
| LC-LS | | miR-1390-5p | | 91.0425 | 0.01 | | -22.48404743 | | | 0 | ** | | |
| LC-LS | | miR-139-3p | | 2.5196 | 0.01 | | -13.63685862 | | | 5.13E-10 | ** | | |
| LC-LS | | miR-1396 | | 1.2598 | 0.01 | | -11.92734731 | | | 2.24E-05 | ** | | |
| LC-LS | | miR-1-3p | | 0.01 | 2.2638 | | 13.37282764 | | | 6.44E-09 | ** | | |
| LC-LS | | miR-1421j-5p | | 7.8948 | 37.919 | | 3.870241746 | | | 5.00E-58 | ** | | |
| LC-LS | | miR-1422i-3p | | 0.01 | 2.1021 | | 13.19005488 | | | 2.48E-08 | ** | | |
| LC-LS | | miR1432-3p | | 1.2598 | 0.01 | | -11.92734731 | | | 2.24E-05 | ** | | |
| LC-LS | | miR1432-5p | | 156.8888 | 23.285 | | -4.705035924 | | | 2.43E-296 | ** | | |
| LC-LS | | miR1436 | | 5.4592 | 0.01 | | -15.54380964 | | | 7.63E-21 | ** | | |
| LC-LS | | miR-144-3p | | 6.971 | 0.01 | | -16.14671337 | | | 2.06E-26 | ** | | |
| LC-LS | | miR-1456-5p | | 22.8446 | 0.01 | | -19.07410729 | | | 6.95E-85 | ** | | |
| LC-LS | | miR-1497g | | 0.01 | 60.8806 | | 21.4915774 | | | 3.14E-221 | ** | | |
| LC-LS | | miR-150-3p | | 33.763 | 21.9914 | | -1.057338973 | | | 3.55E-08 | ** | | |
| LC-LS | | miR156f-3p | | 33.3431 | 20.7786 | | -1.1663821 | | | 2.28E-09 | ** | | |
| LC-LS | | miR164a-3p | | 1.2598 | 0.01 | | -11.92734731 | | | 2.24E-05 | ** | | |
| LC-LS | | miR164a-5p | | 1107.6279 | 696.0435 | | -1.145755359 | | | 2.37E-252 | ** | | |
| LC-LS | | miR-1676-3p | | 8.3988 | 0.01 | | -16.60626 | | | 1.13E-31 | ** | | |
| LC-LS | | miR168a-3p | | 46.7811 | 27.4892 | | -1.311299201 | | | 4.34E-15 | ** | | |
| LC-LS | | miR169e-3p | | 18.0573 | 7.6 | | -2.13434343 | | | 3.01E-13 | ** | | |
| LC-LS | | miR-17 | | 0.01 | 14.068 | | 17.87841359 | | | 1.13E-51 | ** | | |
| LC-LS | | miR171b-3p | | 34.6029 | 0.01 | | -20.09817264 | | | 3.38E-128 | ** | | |
| LC-LS | | miR-1759-5p | | 10.5824 | 18.6765 | | 1.40104239 | | | 1.68E-07 | ** | | |
| LC-LS | | miR-1767 | | 105.2364 | 0.01 | | -22.841371 | | | 0 | ** | | |
| LC-LS | | miR-1770 | | 7.055 | 4.2851 | | -1.229680893 | | | 0.004083 | ** | | |
| LC-LS | | miR-1783 | | 0.01 | 3.234 | | 14.25249629 | | | 1.97E-12 | ** | | |
| LC-LS | | miR-1788a-3p | | 0.01 | 5.0936 | | 15.37285185 | | | 3.62E-19 | ** | | |
| LC-LS | | miR-1794 | | 0.01 | 3.8 | | 14.65026493 | | | 1.76E-14 | ** | | |
| LC-LS | | miR-181a-2-3p | | 55.8517 | 0.01 | | -21.27894574 | | | 1.81E-206 | ** | | |
| LC-LS | | miR-1839 | | 0.01 | 3.3149 | | 14.31343296 | | | 1.00E-12 | ** | | |
| LC-LS | | miR-1839-5p | | 1.7637 | 0.01 | | -12.75716199 | | | 3.12E-07 | ** | | |
| LC-LS | | miR-184 | | 59.2112 | 0.01 | | -21.42300418 | | | 7.63E-219 | ** | | |
| LC-LS | | miR1863 | | 0.01 | 8.3276 | | 16.58526307 | | | 7.00E-31 | ** | | |
| LC-LS | | miR1870-3p | | 0.01 | 3.7191 | | 14.59719165 | | | 3.45E-14 | ** | | |
| LC-LS | | miR1873 | | 12.2622 | 0.01 | | -17.53958961 | | | 6.65E-46 | ** | | |
| LC-LS | | miR-1932 | | 0.01 | 7.4383 | | 16.3067361 | | | 1.17E-27 | ** | | |
| LC-LS | | miR-1934-3p | | 1.2598 | 0.01 | | -11.92734731 | | | 2.24E-05 | ** | | |
| LC-LS | | miR-1957a | | 61.3949 | 14.9574 | | -3.482717948 | | | 3.50E-82 | ** | | |
| LC-LS | | miR-1970 | | 11.2543 | 0.01 | | -17.32805188 | | | 3.43E-42 | ** | | |
| LC-LS | | miR-1a-3p | | 2.4356 | 0.01 | | -13.55323358 | | | 1.05E-09 | ** | | |
| LC-LS | | miR-202 | | 0.01 | 1.617 | | 12.542985 | | | 1.42E-06 | ** | | |
| LC-LS | | miR2086-3p | | 14.8658 | 0.01 | | -18.0144561 | | | 1.71E-55 | ** | | |
| LC-LS | | miR2097-5p | | 33.3431 | 5.7404 | | -4.339021846 | | | 5.72E-59 | ** | | |
| LC-LS | | miR-214 | | 1.1758 | 0.01 | | -11.75716197 | | | 4.58E-05 | ** | | |
| LC-LS | | miR-21-5p | | 2.9396 | 5.1744 | | 1.394570984 | | | 0.0063504 | ** | | |
| LC-LS | | miR-217a | | 0.01 | 1.5362 | | 12.41656043 | | | 2.78E-06 | ** | | |
| LC-LS | | miR-2213-3p | | 0.01 | 15.0382 | | 18.04289363 | | | 3.46E-55 | ** | | |
| LC-LS | | miR-223 | | 0.01 | 2.0213 | | 13.09338571 | | | 4.87E-08 | ** | | |
| LC-LS | | miR-2238d | | 0.01 | 2.3447 | | 13.45942607 | | | 3.28E-09 | ** | | |
| LC-LS | | miR-2243 | | 1.5118 | 0.01 | | -12.37707284 | | | 2.65E-06 | ** | | |
| LC-LS | | miR-2270 | | 0.01 | 37.4339 | | 20.29212067 | | | 2.64E-136 | ** | | |
| LC-LS | | miR-2285aa | | 0.01 | 2.1021 | | 13.19005488 | | | 2.48E-08 | ** | | |
| LC-LS | | miR-2300b-3p | | 0.01 | 5.0127 | | 15.33336593 | | | 7.11E-19 | ** | | |
| LC-LS | | miR-2303 | | 0.01 | 2.3447 | | 13.45942607 | | | 3.28E-09 | ** | | |
| LC-LS | | miR-2358 | | 1.5118 | 0.01 | | -12.37707284 | | | 2.65E-06 | ** | | |
| LC-LS | | miR-2392 | | 14.6978 | 1.3745 | | -5.844171901 | | | 8.00E-36 | ** | | |
| LC-LS | | miR-239a-5p | | 26.792 | 10.3489 | | -2.346004826 | | | 1.12E-21 | ** | | |
| LC-LS | | miR-23a | | 2.0997 | 0.01 | | -13.18723747 | | | 1.81E-08 | ** | | |
| LC-LS | | miR-23a-5p | | 0.01 | 10.5106 | | 17.15944064 | | | 8.68E-39 | ** | | |
| LC-LS | | miR-240-5p | | 0.01 | 1.7787 | | 12.77804882 | | | 3.68E-07 | ** | | |
| LC-LS | | miR-2406 | | 0.01 | 287.2625 | | 25.31800249 | | | 0 | ** | | |
| LC-LS | | miR-2408 | | 1.2598 | 4.0425 | | 2.875488699 | | | 2.01E-05 | ** | | |
| LC-LS | | miR-2455 | | 0.01 | 36.0594 | | 20.19985839 | | | 2.51E-131 | ** | | |
| LC-LS | | miR-2461-3p | | 0.01 | 11.3999 | | 17.35975458 | | | 5.22E-42 | ** | | |
| LC-LS | | miR-2467-3p | | 0.01 | 5.3361 | | 15.48756011 | | | 4.79E-20 | ** | | |
| LC-LS | | miR-2478 | | 15.0338 | 9.3787 | | -1.163749555 | | | 6.30E-05 | ** | | |
| LC-LS | | miR-252b | | 1.5118 | 0.01 | | -12.37707284 | | | 2.65E-06 | ** | | |
| LC-LS | | miR-2561-3p | | 264.9807 | 162.5099 | | -1.205820337 | | | 1.68E-67 | ** | | |
| LC-LS | | miR2592a-3p | | 2.9396 | 0.01 | | -14.01709687 | | | 1.46E-11 | ** | | |
| LC-LS | | miR-2-5p | | 43.9255 | 93.3825 | | 1.860108124 | | | 7.47E-50 | ** | | |
| LC-LS | | miR2610a | | 44.5134 | 0.01 | | -20.71931733 | | | 1.06E-164 | ** | | |
| LC-LS | | miR-2739 | | 21.3328 | 0.01 | | -18.90524207 | | | 2.57E-79 | ** | | |
| LC-LS | | miR-278-5p | | 4.2834 | 1.1319 | | -3.282278604 | | | 1.34E-06 | ** | | |
| LC-LS | | miR-279d-5p | | 0.01 | 28.2169 | | 19.59500612 | | | 6.40E-103 | ** | | |
| LC-LS | | miR-27b-5p | | 0.01 | 12.7744 | | 17.64051509 | | | 5.48E-47 | ** | | |
| LC-LS | | miR-27c-5p | | 56.6916 | 0.01 | | -21.31575794 | | | 1.46E-209 | ** | | |
| LC-LS | | miR2948-5p | | 0.01 | 2.4255 | | 13.542985 | | | 1.67E-09 | ** | | |
| LC-LS | | miR-297 | | 1.5118 | 0.01 | | -12.37707284 | | | 2.65E-06 | ** | | |
| LC-LS | | miR-2976 | | 3.1075 | 7.9234 | | 2.308464625 | | | 3.33E-07 | ** | | |
| LC-LS | | miR-29a | | 3.2755 | 0.01 | | -14.28394355 | | | 8.43E-13 | ** | | |
| LC-LS | | miR-3060-3p | | 11.8422 | 0.01 | | -17.453634 | | | 2.34E-44 | ** | | |
| LC-LS | | miR-3071-5p | | 6.2991 | 0.01 | | -15.89674871 | | | 6.15E-24 | ** | | |
| LC-LS | | miR-3075 | | 0.01 | 101.5485 | | 22.75339239 | | | 0 | ** | | |
| LC-LS | | miR-3077-5p | | 0.01 | 3.3149 | | 14.31343296 | | | 1.00E-12 | ** | | |
| LC-LS | | miR-3081-5p | | 2.4356 | 0.01 | | -13.55323358 | | | 1.05E-09 | ** | | |
| LC-LS | | miR-3122 | | 0.01 | 2.0213 | | 13.09338571 | | | 4.87E-08 | ** | | |
| LC-LS | | miR-3141 | | 8.3988 | 0.01 | | -16.60626 | | | 1.13E-31 | ** | | |
| LC-LS | | miR-3149 | | 3.7794 | 9.1361 | | 2.176927954 | | | 1.59E-07 | ** | | |
| LC-LS | | miR-3154 | | 73.1532 | 25.468 | | -2.602278451 | | | 1.44E-65 | ** | | |
| LC-LS | | miR-3167-3p | | 1.7637 | 0.01 | | -12.75716199 | | | 3.12E-07 | ** | | |
| LC-LS | | miR-317-3p | | 17.3854 | 11.3999 | | -1.040844651 | | | 9.72E-05 | ** | | |
| LC-LS | | miR-3193 | | 0.01 | 56.5146 | | 21.30804606 | | | 2.05E-205 | ** | | |
| LC-LS | | miR319a-5p | | 3.3595 | 1.6979 | | -1.683005387 | | | 0.0099233 | ** | | |
| LC-LS | | miR-3407 | | 0.01 | 9.0553 | | 16.79187746 | | | 1.62E-33 | ** | | |
| LC-LS | | miR-34-3p | | 0.01 | 16.1701 | | 18.22187384 | | | 2.75E-59 | ** | | |
| LC-LS | | miR-3471 | | 0.01 | 2.183 | | 13.28319048 | | | 1.26E-08 | ** | | |
| LC-LS | | miR-3473b | | 0.01 | 13.4212 | | 17.76233167 | | | 2.49E-49 | ** | | |
| LC-LS | | miR-3544-5p | | 0.01 | 2.6681 | | 13.77809504 | | | 2.21E-10 | ** | | |
| LC-LS | | miR-354-5p | | 2.0157 | 0.01 | | -13.08654336 | | | 3.69E-08 | ** | | |
| LC-LS | | miR-3569 | | 0.01 | 38.3232 | | 20.35002638 | | | 1.59E-139 | ** | | |
| LC-LS | | miR-3573-5p | | 6.887 | 0.01 | | -16.11681413 | | | 4.20E-26 | ** | | |
| LC-LS | | miR-3620-5p | | 1.2598 | 0.01 | | -11.92734731 | | | 2.24E-05 | ** | | |
| LC-LS | | miR-3660 | | 1.1758 | 0.01 | | -11.75716197 | | | 4.58E-05 | ** | | |
| LC-LS | | miR-3675-3p | | 0.01 | 27.8935 | | 19.56657606 | | | 9.49E-102 | ** | | |
| LC-LS | | miR-3741 | | 0.01 | 5.4978 | | 15.56118657 | | | 1.24E-20 | ** | | |
| LC-LS | | miR-374-5p | | 7.9788 | 0.01 | | -16.47973666 | | | 3.99E-30 | ** | | |
| LC-LS | | miR-3781 | | 0.01 | 2.1021 | | 13.19005488 | | | 2.48E-08 | ** | | |
| LC-LS | | miR-3925-5p | | 16.7135 | 0.01 | | -18.30339247 | | | 2.67E-62 | ** | | |
| LC-LS | | miR-3927 | | 34.1829 | 0.01 | | -20.06805432 | | | 1.19E-126 | ** | | |
| LC-LS | | miR-3927-3p | | 41.2379 | 19.4042 | | -1.859267746 | | | 5.02E-23 | ** | | |
| LC-LS | | miR394a | | 1.6798 | 5.4978 | | 2.924230097 | | | 4.51E-07 | ** | | |
| LC-LS | | miR-3956 | | 1.2598 | 0.01 | | -11.92734731 | | | 2.24E-05 | ** | | |
| LC-LS | | miR395b | | 0.01 | 8.651 | | 16.67922831 | | | 4.72E-32 | ** | | |
| LC-LS | | miR395b-5p | | 42.6657 | 0.01 | | -20.61475916 | | | 6.75E-158 | ** | | |
| LC-LS | | miR-3968 | | 0.01 | 5.1744 | | 15.41166786 | | | 1.85E-19 | ** | | |
| LC-LS | | miR398a-5p | | 58.1194 | 0.01 | | -21.37710362 | | | 8.03E-215 | ** | | |
| LC-LS | | miR398b-5p | | 0.01 | 4.2042 | | 14.89956618 | | | 6.03E-16 | ** | | |
| LC-LS | | miR-4000c-5p | | 9.0707 | 0.01 | | -16.79606834 | | | 3.80E-34 | ** | | |
| LC-LS | | miR-4039-5p | | 0.01 | 3.1532 | | 14.190094 | | | 3.87E-12 | ** | | |
| LC-LS | | miR-4045-5p | | 0.01 | 18.6765 | | 18.57727356 | | | 2.30E-68 | ** | | |
| LC-LS | | miR-4077d-5p | | 1.3438 | 0.01 | | -12.08654334 | | | 1.10E-05 | ** | | |
| LC-LS | | miR408-3p | | 24.0204 | 15.1999 | | -1.128616517 | | | 8.41E-07 | ** | | |
| LC-LS | | miR-41b | | 0.01 | 1.8596 | | 12.88774667 | | | 1.87E-07 | ** | | |
| LC-LS | | miR-4216-3p | | 2.0157 | 0.01 | | -13.08654336 | | | 3.69E-08 | ** | | |
| LC-LS | | miR4225 | | 3.4435 | 0.01 | | -14.40730276 | | | 2.03E-13 | ** | | |
| LC-LS | | miR-4298 | | 4.0314 | 6.4681 | | 1.165991066 | | | 0.0089232 | ** | | |
| LC-LS | | miR-4311 | | 0.01 | 11.4808 | | 17.37719503 | | | 2.66E-42 | ** | | |
| LC-LS | | miR-432 | | 0.01 | 2.5064 | | 13.62390384 | | | 8.51E-10 | ** | | |
| LC-LS | | miR-432-5p | | 1.3438 | 0.01 | | -12.08654334 | | | 1.10E-05 | ** | | |
| LC-LS | | miR4386 | | 13.69 | 8.4085 | | -1.202131896 | | | 8.55E-05 | ** | | |
| LC-LS | | miR4387d | | 0.01 | 38.3232 | | 20.35002638 | | | 1.59E-139 | ** | | |
| LC-LS | | miR4391 | | 0.01 | 1.5362 | | 12.41656043 | | | 2.78E-06 | ** | | |
| LC-LS | | miR4398 | | 0.01 | 32.3403 | | 19.93139276 | | | 7.43E-118 | ** | | |
| LC-LS | | miR-4433a-3p | | 2.9396 | 7.2766 | | 2.235433294 | | | 1.84E-06 | ** | | |
| LC-LS | | miR-4496 | | 43.6735 | 6.3064 | | -4.772732554 | | | 4.47E-85 | ** | | |
| LC-LS | | miR-4515 | | 170.9147 | 0.01 | | -24.03741999 | | | 0 | ** | | |
| LC-LS | | miR-4520b | | 0.01 | 727.4945 | | 27.60971635 | | | 0 | ** | | |
| LC-LS | | miR-458b-3p | | 0.01 | 1.9404 | | 12.99264527 | | | 9.55E-08 | ** | | |
| LC-LS | | miR-4614 | | 0.01 | 1.0511 | | 11.48066092 | | | 0.0001589 | ** | | |
| LC-LS | | miR-4632-5p | | 2.7716 | 0.01 | | -13.87195805 | | | 6.06E-11 | ** | | |
| LC-LS | | miR-4637 | | 0.01 | 4.851 | | 15.25249629 | | | 2.74E-18 | ** | | |
| LC-LS | | miR-4646-5p | | 9.1546 | 2.0213 | | -3.725389896 | | | 1.23E-14 | ** | | |
| LC-LS | | miR-4657 | | 2.6036 | 0.01 | | -13.71774086 | | | 2.52E-10 | ** | | |
| LC-LS | | miR-465a-5p | | 1.5118 | 0.01 | | -12.37707284 | | | 2.65E-06 | ** | | |
| LC-LS | | miR-4758-5p | | 0.01 | 1.9404 | | 12.99264527 | | | 9.55E-08 | ** | | |
| LC-LS | | miR-4769-5p | | 0.01 | 31.1275 | | 19.83712458 | | | 1.83E-113 | ** | | |
| LC-LS | | miR477b-3p | | 16.7975 | 0.01 | | -18.31575659 | | | 1.31E-62 | ** | | |
| LC-LS | | miR-4788 | | 1.2598 | 0.01 | | -11.92734731 | | | 2.24E-05 | ** | | |
| LC-LS | | miR-4800-5p | | 0.01 | 1.4553 | | 12.283134 | | | 5.46E-06 | ** | | |
| LC-LS | | miR-4850-5p | | 0.01 | 21.2637 | | 18.8972403 | | | 9.78E-78 | ** | | |
| LC-LS | | miR-4887 | | 5.5432 | 9.6212 | | 1.359912437 | | | 0.0002587 | ** | | |
| LC-LS | | miR-4935 | | 0.01 | 3.8808 | | 14.70215656 | | | 8.95E-15 | ** | | |
| LC-LS | | miR-4952-5p | | 1.5958 | 10.4297 | | 4.629947897 | | | 1.28E-20 | ** | | |
| LC-LS | | miR-4956-3p | | 0.01 | 2.3447 | | 13.45942607 | | | 3.28E-09 | ** | | |
| LC-LS | | miR-4969-3p | | 2.2677 | 0.01 | | -13.37707284 | | | 4.35E-09 | ** | | |
| LC-LS | | miR-4987-5p | | 67.526 | 0.01 | | -21.74708195 | | | 1.80E-249 | ** | | |
| LC-LS | | miR5023 | | 0.01 | 178.7609 | | 24.14811817 | | | 0 | ** | | |
| LC-LS | | miR-50-3p | | 0.01 | 111.7356 | | 22.98916815 | | | 0 | ** | | |
| LC-LS | | miR5073 | | 100.6171 | 61.204 | | -1.226023308 | | | 1.94E-27 | ** | | |
| LC-LS | | miR5076 | | 10.2465 | 0.01 | | -17.09667795 | | | 1.77E-38 | ** | | |
| LC-LS | | miR5077 | | 61.7309 | 275.4583 | | 3.688730812 | | | 0 | ** | | |
| LC-LS | | miR-5110 | | 23.0966 | 12.6127 | | -1.492067165 | | | 7.51E-10 | ** | | |
| LC-LS | | miR-5114 | | 1.0079 | 0.01 | | -11.37715442 | | | 0.0001902 | ** | | |
| LC-LS | | miR-5126 | | 3.7794 | 8.651 | | 2.042369705 | | | 1.23E-06 | ** | | |
| LC-LS | | miR-5130 | | 1.0079 | 0.01 | | -11.37715442 | | | 0.0001902 | ** | | |
| LC-LS | | miR-5194 | | 0.01 | 74.8677 | | 22.00162866 | | | 6.85E-272 | ** | | |
| LC-LS | | miR-5197-3p | | 83.4836 | 0.01 | | -22.2702774 | | | 2.98E-308 | ** | | |
| LC-LS | | miR5227 | | 47.7049 | 31.5318 | | -1.02114226 | | | 2.12E-10 | ** | | |
| LC-LS | | miR528-5p | | 3484.1397 | 10142.557 | | 2.635291986 | | | 0 | ** | | |
| LC-LS | | miR5286a | | 19.1492 | 0.01 | | -18.63891868 | | | 2.85E-71 | ** | | |
| LC-LS | | miR5290 | | 5.8791 | 0.01 | | -15.72656625 | | | 2.16E-22 | ** | | |
| LC-LS | | miR5303a | | 2.6036 | 0.01 | | -13.71774086 | | | 2.52E-10 | ** | | |
| LC-LS | | miR530b | | 0.01 | 13.1787 | | 17.71736195 | | | 1.88E-48 | ** | | |
| LC-LS | | miR531 | | 31.0754 | 17.868 | | -1.364864926 | | | 3.66E-11 | ** | | |
| LC-LS | | miR-5310 | | 64.9224 | 0.01 | | -21.65010748 | | | 7.00E-240 | ** | | |
| LC-LS | | miR-5315 | | 1.5958 | 0.01 | | -12.51043621 | | | 1.30E-06 | ** | | |
| LC-LS | | miR-5347-3p | | 2.5196 | 0.01 | | -13.63685862 | | | 5.13E-10 | ** | | |
| LC-LS | | miR535 | | 95.3259 | 0.01 | | -22.5974354 | | | 0 | ** | | |
| LC-LS | | miR5380a | | 0.01 | 4.5276 | | 15.08233893 | | | 4.06E-17 | ** | | |
| LC-LS | | miR-541-3p | | 10.5824 | 0.01 | | -17.17623124 | | | 1.02E-39 | ** | | |
| LC-LS | | miR-5421 | | 2.3517 | 0.01 | | -13.46677813 | | | 2.13E-09 | ** | | |
| LC-LS | | miR-5451 | | 0.01 | 99.4463 | | 22.70180048 | | | 0 | ** | | |
| LC-LS | | miR-5459 | | 0.01 | 20.3744 | | 18.79187443 | | | 1.63E-74 | ** | | |
| LC-LS | | miR-5460 | | 2.4356 | 4.4468 | | 1.484693974 | | | 0.0076878 | ** | | |
| LC-LS | | miR5502 | | 9.4906 | 16.6552 | | 1.387099992 | | | 9.73E-07 | ** | | |
| LC-LS | | miR-550a-5p | | 1.0079 | 0.01 | | -11.37715442 | | | 0.0001902 | ** | | |
| LC-LS | | miR5522 | | 1.6798 | 0.01 | | -12.63695646 | | | 6.37E-07 | ** | | |
| LC-LS | | miR5524 | | 0.01 | 8.5702 | | 16.65608487 | | | 9.26E-32 | ** | | |
| LC-LS | | miR5565g-3p | | 12.5981 | 0.01 | | -17.60624043 | | | 3.85E-47 | ** | | |
| LC-LS | | miR-5612-3p | | 0.01 | 3.234 | | 14.25249629 | | | 1.97E-12 | ** | | |
| LC-LS | | miR5659 | | 0.01 | 3.9617 | | 14.75304113 | | | 4.56E-15 | ** | | |
| LC-LS | | miR5662 | | 0.01 | 115.2931 | | 23.06646757 | | | 0 | ** | | |
| LC-LS | | miR5668 | | 1.3438 | 0.01 | | -12.08654334 | | | 1.10E-05 | ** | | |
| LC-LS | | miR5673 | | 0.01 | 17.1403 | | 18.36558179 | | | 8.42E-63 | ** | | |
| LC-LS | | miR-57 | | 50.2246 | 0.01 | | -21.0170371 | | | 9.68E-186 | ** | | |
| LC-LS | | miR5720 | | 24.1044 | 0.01 | | -19.20649759 | | | 1.59E-89 | ** | | |
| LC-LS | | miR-5735-3p | | 6.551 | 0.01 | | -15.99345484 | | | 7.25E-25 | ** | | |
| LC-LS | | miR5780a | | 0.01 | 17.0595 | | 18.35392805 | | | 1.65E-62 | ** | | |
| LC-LS | | miR5802 | | 6.3831 | 0.01 | | -15.92942014 | | | 3.01E-24 | ** | | |
| LC-LS | | miR5803 | | 0.01 | 1.0511 | | 11.48066092 | | | 0.0001589 | ** | | |
| LC-LS | | miR5813 | | 85.7513 | 134.293 | | 1.106316489 | | | 2.81E-30 | ** | | |
| LC-LS | | miR5815 | | 20.9969 | 0.01 | | -18.86609921 | | | 4.45E-78 | ** | | |
| LC-LS | | miR-585-5p | | 2.7716 | 0.01 | | -13.87195805 | | | 6.06E-11 | ** | | |
| LC-LS | | miR-5985 | | 22.3407 | 0.01 | | -19.01909749 | | | 4.99E-83 | ** | | |
| LC-LS | | miR-6002-5p | | 7.8108 | 0.01 | | -16.42725215 | | | 1.66E-29 | ** | | |
| LC-LS | | miR-604 | | 161.5921 | 91.7655 | | -1.39553031 | | | 1.88E-53 | ** | | |
| LC-LS | | miR-6041 | | 2.0157 | 0.01 | | -13.08654336 | | | 3.69E-08 | ** | | |
| LC-LS | | miR-6048 | | 5.8791 | 0.01 | | -15.72656625 | | | 2.16E-22 | ** | | |
| LC-LS | | miR-6056 | | 203.1659 | 69.6933 | | -2.63874419 | | | 1.96E-182 | ** | | |
| LC-LS | | miR-6090 | | 2.7716 | 0.01 | | -13.87195805 | | | 6.06E-11 | ** | | |
| LC-LS | | miR6150 | | 8.9867 | 15.685 | | 1.37363024 | | | 2.45E-06 | ** | | |
| LC-LS | | miR6182 | | 4.1994 | 0.01 | | -14.89674871 | | | 3.33E-16 | ** | | |
| LC-LS | | miR6184 | | 0.01 | 1.2936 | | 11.99264527 | | | 2.10E-05 | ** | | |
| LC-LS | | miR6196 | | 31.8313 | 15.7659 | | -1.732826547 | | | 2.55E-16 | ** | | |
| LC-LS | | miR6224a-3p | | 0.01 | 7.6808 | | 16.38585851 | | | 1.54E-28 | ** | | |
| LC-LS | | miR6231-3p | | 0.01 | 9.5404 | | 16.92058198 | | | 2.84E-35 | ** | | |
| LC-LS | | miR6252 | | 1.5958 | 0.01 | | -12.51043621 | | | 1.30E-06 | ** | | |
| LC-LS | | miR-626 | | 1.0079 | 0.01 | | -11.37715442 | | | 0.0001902 | ** | | |
| LC-LS | | miR6281 | | 4.6193 | 0.01 | | -15.13179118 | | | 9.46E-18 | ** | | |
| LC-LS | | miR6297a | | 0.01 | 7.9234 | | 16.46255242 | | | 2.04E-29 | ** | | |
| LC-LS | | miR-630 | | 1.7637 | 0.01 | | -12.75716199 | | | 3.12E-07 | ** | | |
| LC-LS | | miR-6302-1-5p | | 25.8682 | 0.01 | | -19.38066804 | | | 5.06E-96 | ** | | |
| LC-LS | | miR-6311-3p | | 0.01 | 2.5872 | | 13.70215657 | | | 4.34E-10 | ** | | |
| LC-LS | | miR-6-3-5p | | 0.01 | 10.4297 | | 17.14038411 | | | 1.70E-38 | ** | | |
| LC-LS | | miR6485 | | 6.2151 | 0.01 | | -15.86363871 | | | 1.25E-23 | ** | | |
| LC-LS | | miR-6511b-5p | | 3.0236 | 0.01 | | -14.08658413 | | | 7.15E-12 | ** | | |
| LC-LS | | miR-6525 | | 28.4718 | 18.1106 | | -1.115796926 | | | 1.10E-07 | ** | | |
| LC-LS | | miR-6527 | | 150.4217 | 91.8464 | | -1.216689325 | | | 1.12E-39 | ** | | |
| LC-LS | | miR-6547-5p | | 0.01 | 40.8296 | | 20.50627127 | | | 1.33E-148 | ** | | |
| LC-LS | | miR-6549-3p | | 2.2677 | 0.01 | | -13.37707284 | | | 4.35E-09 | ** | | |
| LC-LS | | miR-6574-3p | | 0.01 | 90.6336 | | 22.47294528 | | | 0 | ** | | |
| LC-LS | | miR-6590-5p | | 0.01 | 7.1149 | | 16.19710607 | | | 1.73E-26 | ** | | |
| LC-LS | | miR-6591-3p | | 0.01 | 2.0213 | | 13.09338571 | | | 4.87E-08 | ** | | |
| LC-LS | | miR-6619-5p | | 2.4356 | 0.01 | | -13.55323358 | | | 1.05E-09 | ** | | |
| LC-LS | | miR-6626-5p | | 303.447 | 1.6979 | | -12.78979294 | | | 0 | ** | | |
| LC-LS | | miR-663 | | 3.2755 | 0.01 | | -14.28394355 | | | 8.43E-13 | ** | | |
| LC-LS | | miR-6632-5p | | 0.01 | 2.5064 | | 13.62390384 | | | 8.51E-10 | ** | | |
| LC-LS | | miR-6661-5p | | 0.01 | 1.2936 | | 11.99264527 | | | 2.10E-05 | ** | | |
| LC-LS | | miR-6673-3p | | 4.5353 | 0.01 | | -15.08652977 | | | 1.93E-17 | ** | | |
| LC-LS | | miR-6704-5p | | 0.01 | 3.8808 | | 14.70215656 | | | 8.95E-15 | ** | | |
| LC-LS | | miR-6747-5p | | 2.0997 | 0.01 | | -13.18723747 | | | 1.81E-08 | ** | | |
| LC-LS | | miR-6756-5p | | 1.4278 | 0.01 | | -12.23608368 | | | 5.40E-06 | ** | | |
| LC-LS | | miR-6769b-5p | | 0.01 | 17.6254 | | 18.43441292 | | | 1.47E-64 | ** | | |
| LC-LS | | miR-6777-5p | | 0.01 | 62.821 | | 21.56895728 | | | 2.95E-228 | ** | | |
| LC-LS | | miR-6800-5p | | 0.7559 | 3.8808 | | 4.034595016 | | | 1.91E-07 | ** | | |
| LC-LS | | miR-6802-5p | | 0.01 | 22.7999 | | 19.06927669 | | | 2.67E-83 | ** | | |
| LC-LS | | miR-6849-5p | | 3.8634 | 37.353 | | 5.595711103 | | | 2.45E-84 | ** | | |
| LC-LS | | miR-6856-5p | | 4.4513 | 2.2638 | | -1.667594468 | | | 0.0031615 | ** | | |
| LC-LS | | miR-6877-5p | | 0.01 | 1.0511 | | 11.48066092 | | | 0.0001589 | ** | | |
| LC-LS | | miR-6887-5p | | 18.8972 | 228.0798 | | 6.142777162 | | | 0 | ** | | |
| LC-LS | | miR-6891-5p | | 0.01 | 87.0762 | | 22.37419102 | | | 4.13277246834775e-316 | ** | | |
| LC-LS | | miR-6922-5p | | 4.2834 | 7.5191 | | 1.387787386 | | | 0.0010265 | ** | | |
| LC-LS | | miR-6946-5p | | 0.01 | 4.6893 | | 15.16888477 | | | 1.06E-17 | ** | | |
| LC-LS | | miR-6973b-5p | | 4.2834 | 0.01 | | -14.94559497 | | | 1.64E-16 | ** | | |
| LC-LS | | miR-6978-5p | | 0.01 | 5.417 | | 15.52467084 | | | 2.44E-20 | ** | | |
| LC-LS | | miR-6980-5p | | 69.8776 | 46.0849 | | -1.02662297 | | | 1.11E-14 | ** | | |
| LC-LS | | miR-7000-5p | | 0.01 | 131.3015 | | 23.38713296 | | | 0 | ** | | |
| LC-LS | | miR-7001-5p | | 0.01 | 2.9915 | | 14.06026068 | | | 1.49E-11 | ** | | |
| LC-LS | | miR-7007-5p | | 3.1075 | 72.7656 | | 7.777302433 | | | 3.48E-209 | ** | | |
| LC-LS | | miR-7027-5p | | 0.01 | 4.5276 | | 15.08233893 | | | 4.06E-17 | ** | | |
| LC-LS | | miR-7039-5p | | 0.01 | 4.1234 | | 14.85170519 | | | 1.18E-15 | ** | | |
| LC-LS | | miR-7040-5p | | 45.4373 | 97.7485 | | 1.88934752 | | | 2.18E-53 | ** | | |
| LC-LS | | miR-7084-5p | | 2.8556 | 4.9319 | | 1.347692611 | | | 0.0097018 | ** | | |
| LC-LS | | miR-7111-5p | | 16.7135 | 2.1021 | | -5.113337464 | | | 6.63E-36 | ** | | |
| LC-LS | | miR-7117-5p | | 0.01 | 4.7702 | | 15.21107069 | | | 5.38E-18 | ** | | |
| LC-LS | | miR-7201-5p | | 0.01 | 5.417 | | 15.52467084 | | | 2.44E-20 | ** | | |
| LC-LS | | miR-7216-5p | | 0.01 | 14.3106 | | 17.92058198 | | | 1.50E-52 | ** | | |
| LC-LS | | miR-7224-3p | | 0.01 | 8.7319 | | 16.70218482 | | | 2.41E-32 | ** | | |
| LC-LS | | miR-7267-5p | | 159.3244 | 539.5166 | | 3.008227225 | | | 0 | ** | | |
| LC-LS | | miR-7275-3p | | 1.7637 | 0.01 | | -12.75716199 | | | 3.12E-07 | ** | | |
| LC-LS | | miR-7355-5p | | 0.01 | 2.6681 | | 13.77809504 | | | 2.21E-10 | ** | | |
| LC-LS | | miR-7380-3p | | 1.6798 | 0.01 | | -12.63695646 | | | 6.37E-07 | ** | | |
| LC-LS | | miR-7384-3p | | 18.1413 | 76.889 | | 3.561765699 | | | 1.42E-104 | ** | | |
| LC-LS | | miR-7423-3p | | 0.01 | 1.7787 | | 12.77804882 | | | 3.68E-07 | ** | | |
| LC-LS | | miR-7439-5p | | 0.01 | 11.8042 | | 17.44570727 | | | 1.79E-43 | ** | | |
| LC-LS | | miR-7447-3p | | 17.9733 | 0.01 | | -18.48261979 | | | 6.10E-67 | ** | | |
| LC-LS | | miR-7449-3p | | 0.01 | 4.0425 | | 14.80283599 | | | 2.32E-15 | ** | | |
| LC-LS | | miR-7457-5p | | 3.9474 | 1.617 | | -2.201137745 | | | 0.0005183 | ** | | |
| LC-LS | | miR-7458-5p | | 2.3517 | 0.01 | | -13.46677813 | | | 2.13E-09 | ** | | |
| LC-LS | | miR-748-3p | | 0.01 | 3.6383 | | 14.54301889 | | | 6.76E-14 | ** | | |
| LC-LS | | miR7497 | | 1.4278 | 3.3957 | | 2.136743977 | | | 0.0017668 | ** | | |
| LC-LS | | miR-7515 | | 566.916 | 0.01 | | -26.99463152 | | | 0 | ** | | |
| LC-LS | | miR7522 | | 0.01 | 1.9404 | | 12.99264527 | | | 9.55E-08 | ** | | |
| LC-LS | | miR7539 | | 12.0942 | 0.01 | | -17.50556599 | | | 2.77E-45 | ** | | |
| LC-LS | | miR-7671-3p | | 0.01 | 6.0638 | | 15.80285633 | | | 1.11E-22 | ** | | |
| LC-LS | | miR-770-3p | | 0.01 | 1.2128 | | 11.83357539 | | | 4.13E-05 | ** | | |
| LC-LS | | miR774a-5p.1 | | 0.01 | 10.268 | | 17.10184752 | | | 6.56E-38 | ** | | |
| LC-LS | | miR774b-5p | | 8.0628 | 0.01 | | -16.50556599 | | | 1.96E-30 | ** | | |
| LC-LS | | miR7783-3p | | 3.5275 | 5.9021 | | 1.269452792 | | | 0.0071529 | ** | | |
| LC-LS | | miR7800 | | 0.01 | 54.0891 | | 21.19985839 | | | 1.25E-196 | ** | | |
| LC-LS | | miR7811 | | 0.01 | 92.8166 | | 22.53164455 | | | 0 | ** | | |
| LC-LS | | miR7837 | | 0.01 | 3.234 | | 14.25249629 | | | 1.97E-12 | ** | | |
| LC-LS | | miR-7888-5p | | 1.1758 | 0.01 | | -11.75716197 | | | 4.58E-05 | ** | | |
| LC-LS | | miR-7894-3p | | 0.01 | 1.0511 | | 11.48066092 | | | 0.0001589 | ** | | |
| LC-LS | | miR-790 | | 0.01 | 1.9404 | | 12.99264527 | | | 9.55E-08 | ** | | |
| LC-LS | | miR-790-3p | | 0.01 | 17.7063 | | 18.44570728 | | | 7.51E-65 | ** | | |
| LC-LS | | miR-7922-5p | | 1.2598 | 0.01 | | -11.92734731 | | | 2.24E-05 | ** | | |
| LC-LS | | miR-7942-3p | | 2.0157 | 0.01 | | -13.08654336 | | | 3.69E-08 | ** | | |
| LC-LS | | miR8026 | | 0.01 | 4.6893 | | 15.16888477 | | | 1.06E-17 | ** | | |
| LC-LS | | miR-8100 | | 0.01 | 1.2936 | | 11.99264527 | | | 2.10E-05 | ** | | |
| LC-LS | | miR-8109 | | 20.7449 | 0.01 | | -18.83632019 | | | 3.77E-77 | ** | | |
| LC-LS | | miR812a | | 3.0236 | 0.01 | | -14.08658413 | | | 7.15E-12 | ** | | |
| LC-LS | | miR-8189-3p | | 1.3438 | 0.01 | | -12.08654334 | | | 1.10E-05 | ** | | |
| LC-LS | | miR-8192-5p | | 4.8713 | 0.01 | | -15.26279551 | | | 1.12E-18 | ** | | |
| LC-LS | | miR-8207-3p | | 0.01 | 1.6979 | | 12.66338897 | | | 7.22E-07 | ** | | |
| LC-LS | | miR-8250c-3p | | 1.1758 | 0.01 | | -11.75716197 | | | 4.58E-05 | ** | | |
| LC-LS | | miR-8263-5p | | 11.2543 | 0.01 | | -17.32805188 | | | 3.43E-42 | ** | | |
| LC-LS | | miR828-3p | | 17.0495 | 0.01 | | -18.35248177 | | | 1.54E-63 | ** | | |
| LC-LS | | miR-8293-3p | | 0.01 | 3.3149 | | 14.31343296 | | | 1.00E-12 | ** | | |
| LC-LS | | miR-8295-3p | | 1.6798 | 0.01 | | -12.63695646 | | | 6.37E-07 | ** | | |
| LC-LS | | miR-8309-3p | | 13.438 | 5.983 | | -1.995644966 | | | 2.48E-09 | ** | | |
| LC-LS | | miR-8321-3p | | 1.0079 | 0.01 | | -11.37715442 | | | 0.0001902 | ** | | |
| LC-LS | | miR-8330-5p | | 58.7913 | 109.7952 | | 1.540509689 | | | 3.71E-43 | ** | | |
| LC-LS | | miR848-3p | | 6.467 | 3.8 | | -1.311361308 | | | 0.0036508 | ** | | |
| LC-LS | | miR-84a | | 0.01 | 368.9217 | | 25.93504201 | | | 0 | ** | | |
| LC-LS | | miR8575 | | 39.9781 | 0.01 | | -20.45429316 | | | 5.36E-148 | ** | | |
| LC-LS | | miR8581 | | 3.2755 | 1.1319 | | -2.620627199 | | | 0.0003178 | ** | | |
| LC-LS | | miR8632 | | 0.01 | 4.9319 | | 15.29328757 | | | 1.40E-18 | ** | | |
| LC-LS | | miR8695 | | 12.4302 | 0.01 | | -17.57315 | | | 1.60E-46 | ** | | |
| LC-LS | | miR8724 | | 0.01 | 104.3782 | | 22.82117707 | | | 0 | ** | | |
| LC-LS | | miR8751b | | 0.01 | 1.1319 | | 11.66331635 | | | 8.10E-05 | ** | | |
| LC-LS | | miR8772 | | 0.01 | 2.3447 | | 13.45942607 | | | 3.28E-09 | ** | | |
| LC-LS | | miR-87a-3p | | 29.3956 | 0.01 | | -19.69593667 | | | 5.14E-109 | ** | | |
| LC-LS | | miR-8834a | | 2.6036 | 0.01 | | -13.71774086 | | | 2.52E-10 | ** | | |
| LC-LS | | miR-8872 | | 0.01 | 1.5362 | | 12.41656043 | | | 2.78E-06 | ** | | |
| LC-LS | | miR-8908n | | 7.2229 | 0.01 | | -16.23426173 | | | 2.43E-27 | ** | | |
| LC-LS | | miR-8933 | | 3.9474 | 0.01 | | -14.74412271 | | | 2.83E-15 | ** | | |
| LC-LS | | miR-8964 | | 6.971 | 0.01 | | -16.14671337 | | | 2.06E-26 | ** | | |
| LC-LS | | miR-8979 | | 0.01 | 48.1062 | | 20.9107543 | | | 5.84E-175 | ** | | |
| LC-LS | | miR-9005 | | 2.2677 | 0.01 | | -13.37707284 | | | 4.35E-09 | ** | | |
| LC-LS | | miR904a | | 0.01 | 6.3064 | | 15.89960529 | | | 1.47E-23 | ** | | |
| LC-LS | | miR-9155 | | 0.01 | 3.0723 | | 14.12599146 | | | 7.59E-12 | ** | | |
| LC-LS | | miR-9194 | | 57.0275 | 0.01 | | -21.33032805 | | | 8.44E-211 | ** | | |
| LC-LS | | miR-932-3p | | 32.3352 | 15.3616 | | -1.835633974 | | | 4.82E-18 | ** | | |
| LC-LS | | miR946a-5p | | 63.0747 | 24.3361 | | -2.348806032 | | | 6.36E-49 | ** | | |
| LC-LS | | miR9484 | | 0.01 | 120.4675 | | 23.17474427 | | | 0 | ** | | |
| LC-LS | | miR9748 | | 0.01 | 139.144 | | 23.53021102 | | | 0 | ** | | |
| LC-LS | | miR9765 | | 47.537 | 0.01 | | -20.88139806 | | | 7.69E-176 | ** | | |
| LC-LS | | miR9774 | | 882.1213 | 540.4868 | | -1.208141837 | | | 6.24E-221 | ** | | |
| LC-LS | | miR9780 | | 0.01 | 2.5872 | | 13.70215657 | | | 4.34E-10 | ** | | |
| LC-LS | | miR-9788-3p | | 0.01 | 1.4553 | | 12.283134 | | | 5.46E-06 | ** | | |
| LC-LS | | miR-9813-5p | | 0.01 | 131.7866 | | 23.39622804 | | | 0 | ** | | |
| LC-LS | | miR-9829-5p | | 2.8556 | 0.01 | | -13.94559497 | | | 2.97E-11 | ** | | |
| LC-LS | | miR-9872 | | 0.01 | 4.1234 | | 14.85170519 | | | 1.18E-15 | ** | | |
| LC-LS | | miR-9877 | | 3.2755 | 5.9021 | | 1.452252442 | | | 0.0025289 | ** | | |
| LC-LS | | miR-989 | | 9.5746 | 4.1234 | | -2.077702072 | | | 2.08E-07 | ** | | |
| LC-LS | | miR-B2-3p | | 0.01 | 1.3745 | | 12.14225372 | | | 1.07E-05 | ** | | |
| LC-LS | | miR-B2-5p | | 1.6798 | 0.01 | | -12.63695646 | | | 6.37E-07 | ** | | |
| LC-LS | | miR-D3-3p | | 0.01 | 1.3745 | | 12.14225372 | | | 1.07E-05 | ** | | |
| LC-LS | | miR-H1 | | 0.01 | 4.2042 | | 14.89956618 | | | 6.03E-16 | ** | | |
| LC-LS | | miR-H1-5p | | 0.01 | 2.9915 | | 14.06026068 | | | 1.49E-11 | ** | | |
| LC-LS | | miR-H16 | | 0.01 | 8.0042 | | 16.48757553 | | | 1.04E-29 | ** | | |
| LC-LS | | miR-H2-5p | | 0.01 | 1.8596 | | 12.88774667 | | | 1.87E-07 | ** | | |
| LC-LS | | miR-I5-3p | | 28.6398 | 18.9999 | | -1.012081269 | | | 1.06E-06 | ** | | |
| LC-LS | | miR-m107-1-3p | | 1.0079 | 0.01 | | -11.37715442 | | | 0.0001902 | ** | | |
| LC-LS | | miR-M1-13-5p | | 9.6586 | 5.3361 | | -1.463390181 | | | 9.28E-05 | ** | | |
| LC-LS | | miR-rL1-15-3p | | 0.01 | 2.4255 | | 13.542985 | | | 1.67E-09 | ** | | |
| LC-LS | | let-7d | | 6.1311 | 3.8808 | | -1.127921618 | | | 0.0131447 | * | | |
| LC-LS | | let-7e-5p | | 2.4356 | 1.2128 | | -1.719658198 | | | 0.0258974 | * | | |
| LC-LS | | miR-2075 | | 0.5039 | 1.4553 | | 2.61573557 | | | 0.0189144 | * | | |
| LC-LS | | miR-3911 | | 2.4356 | 4.3659 | | 1.439411704 | | | 0.0101066 | * | | |
| LC-LS | | miR393b-3p | | 3.6955 | 2.2638 | | -1.208663921 | | | 0.041286 | * | | |
| LC-LS | | miR399b | | 3.0236 | 4.6893 | | 1.082300625 | | | 0.037496 | * | | |
| LC-LS | | miR-5093 | | 3.8634 | 2.1021 | | -1.501018867 | | | 0.0118931 | * | | |
| LC-LS | | miR5667-3p | | 1.9317 | 3.5574 | | 1.505997648 | | | 0.0160332 | * | | |
| LC-LS | | miR6197 | | 0.5039 | 1.2936 | | 2.325246863 | | | 0.0432541 | * | | |
| LC-LS | | miR-6729-5p | | 0.9239 | 1.7787 | | 1.615513112 | | | 0.0737947 | * | | |
| LC-LS | | miR-6970-5p | | 6.3831 | 4.2042 | | -1.029853964 | | | 0.0196022 | * | | |
| LC-LS | | miR8708 | | 3.4435 | 5.6595 | | 1.225375847 | | | 0.0106739 | * | | |
| LC-LS | | miR-H17 | | 3.0236 | 1.7787 | | -1.308535315 | | | 0.0482831 | * | | |
| LC-LS | | let-7b | | 35.6947 | 25.0637 | | -0.872039608 | | | 1.91E-06 |  | | |
| LC-LS | | let-7c | | 216.016 | 190.6459 | | -0.30812688 | | | 1.16E-05 |  | | |
| LC-LS | | let-7i-5p | | 2.9396 | 1.9404 | | -1.0244516 | | | 0.116474 |  | | |
| LC-LS | | miR-101-3p | | 2.3517 | 3.1532 | | 0.723315853 | | | 0.2387695 |  | | |
| LC-LS | | miR-103a-3p | | 16.7975 | 13.7446 | | -0.49470113 | | | 0.0542679 |  | | |
| LC-LS | | miR1042-3p | | 44.1775 | 51.6636 | | 0.386069876 | | | 0.007763 |  | | |
| LC-LS | | miR1048-5p | | 3.5275 | 2.8298 | | -0.54353226 | | | 0.3366538 |  | | |
| LC-LS | | miR-107 | | 18.3093 | 15.3616 | | -0.432930572 | | | 0.0767624 |  | | |
| LC-LS | | miR1078 | | 10.3305 | 10.1063 | | -0.054114871 | | | 0.8619372 |  | | |
| LC-LS | | miR-10b-3p | | 2.0157 | 2.1021 | | 0.103511541 | | | 0.8862383 |  | | |
| LC-LS | | miR1111 | | 17.4694 | 18.0297 | | 0.077860256 | | | 0.7445102 |  | | |
| LC-LS | | miR1120 | | 2.0997 | 2.9106 | | 0.805407816 | | | 0.2117577 |  | | |
| LC-LS | | miR1122c-3p | | 27.3799 | 22.6382 | | -0.469017979 | | | 0.0194581 |  | | |
| LC-LS | | miR1130b-3p | | 15.7057 | 14.2297 | | -0.243405346 | | | 0.3473155 |  | | |
| LC-LS | | miR1169-3p | | 3.8634 | 2.9915 | | -0.630813068 | | | 0.2473779 |  | | |
| LC-LS | | miR1171 | | 28.8917 | 33.2296 | | 0.345003021 | | | 0.0554856 |  | | |
| LC-LS | | miR-1188-5p | | 1.8477 | 2.183 | | 0.411276962 | | | 0.5677509 |  | | |
| LC-LS | | miR-1193-5p | | 45.8572 | 37.4339 | | -0.500549881 | | | 0.0012925 |  | | |
| LC-LS | | miR-1231-5p | | 5.1232 | 4.0425 | | -0.584306583 | | | 0.2145363 |  | | |
| LC-LS | | miR-1249-5p | | 52.4922 | 50.5317 | | -0.093876701 | | | 0.5007716 |  | | |
| LC-LS | | miR-1306-3p | | 32.3352 | 39.8594 | | 0.515955227 | | | 0.0020464 |  | | |
| LC-LS | | miR-132-3p | | 61.8988 | 47.9445 | | -0.630033754 | | | 3.41E-06 |  | | |
| LC-LS | | miR-133-5p | | 21.5008 | 15.3616 | | -0.82921871 | | | 0.0004175 |  | | |
| LC-LS | | miR-1357 | | 25.1123 | 21.7488 | | -0.354652426 | | | 0.0869185 |  | | |
| LC-LS | | miR-1397-5p | | 46.949 | 56.2721 | | 0.446738671 | | | 0.0013962 |  | | |
| LC-LS | | miR-140 | | 27.296 | 28.4594 | | 0.102939402 | | | 0.5883034 |  | | |
| LC-LS | | miR-140-3p | | 17.5534 | 19.7276 | | 0.287992734 | | | 0.2158432 |  | | |
| LC-LS | | miR-142 | | 5.1232 | 4.2042 | | -0.4875764 | | | 0.2954178 |  | | |
| LC-LS | | miR-14-5p | | 24.5244 | 19.0808 | | -0.619007508 | | | 0.0040495 |  | | |
| LC-LS | | miR-148a-5p | | 0.9239 | 1.2936 | | 0.83010957 | | | 0.3991406 |  | | |
| LC-LS | | miR-149-3p | | 326.1237 | 278.4498 | | -0.389772045 | | | 1.39E-11 |  | | |
| LC-LS | | miR1510a-5p | | 19.0652 | 20.2935 | | 0.153986007 | | | 0.4965047 |  | | |
| LC-LS | | miR1516c | | 1.5118 | 1.7787 | | 0.400975977 | | | 0.6161018 |  | | |
| LC-LS | | miR1520d | | 1.0079 | 0.8085 | | -0.543680714 | | | 0.6115149 |  | | |
| LC-LS | | miR-1556 | | 12.5981 | 9.0553 | | -0.814363005 | | | 0.0079307 |  | | |
| LC-LS | | miR156a-5p | | 71090.09 | 53087.045 | | -0.720197653 | | | 0 |  | | |
| LC-LS | | miR-1579 | | 82.0558 | 69.289 | | -0.417084821 | | | 0.0002976 |  | | |
| LC-LS | | miR157a-5p | | 44.1775 | 65.004 | | 0.952568275 | | | 3.50E-12 |  | | |
| LC-LS | | miR-1581 | | 8.8187 | 7.1957 | | -0.501623539 | | | 0.1579724 |  | | |
| LC-LS | | miR159a.1 | | 94.486 | 98.3144 | | 0.09795869 | | | 0.3372518 |  | | |
| LC-LS | | miR159b-3p | | 59.9671 | 57.9699 | | -0.083539047 | | | 0.5214942 |  | | |
| LC-LS | | miR-1603 | | 6.0471 | 4.6085 | | -0.670036437 | | | 0.125175 |  | | |
| LC-LS | | miR160a-3p | | 10.4984 | 9.4595 | | -0.256997055 | | | 0.417918 |  | | |
| LC-LS | | miR160a-5p | | 19.8211 | 14.8765 | | -0.707740906 | | | 0.0034184 |  | | |
| LC-LS | | miR162a-3p | | 7.5589 | 7.1149 | | -0.149296442 | | | 0.6859163 |  | | |
| LC-LS | | miR165a-3p | | 16.3776 | 12.8553 | | -0.597236027 | | | 0.0231796 |  | | |
| LC-LS | | miR-16-5p | | 1.6798 | 2.183 | | 0.646234006 | | | 0.3799051 |  | | |
| LC-LS | | miR-1664-5p | | 22.4247 | 17.6254 | | -0.593940107 | | | 0.008201 |  | | |
| LC-LS | | miR-1668-5p | | 3.7794 | 2.4255 | | -1.093873606 | | | 0.058716 |  | | |
| LC-LS | | miR166a-3p | | 8921.578 | 6104.8735 | | -0.935679654 | | | 0 |  | | |
| LC-LS | | miR166n | | 62.2348 | 59.9912 | | -0.09055389 | | | 0.4793685 |  | | |
| LC-LS | | miR-1670 | | 840.7994 | 825.728 | | -0.044609714 | | | 0.1983868 |  | | |
| LC-LS | | miR167a-5p | | 4934.6048 | 3789.0674 | | -0.651480633 | | | 0 |  | | |
| LC-LS | | miR167e-3p | | 594.044 | 443.5469 | | -0.72053017 | | | 8.38E-60 |  | | |
| LC-LS | | miR168a-5p | | 86997.584 | 61818.757 | | -0.84267058 | | | 0 |  | | |
| LC-LS | | miR-1694 | | 27.7159 | 30.885 | | 0.267012791 | | | 0.1498753 |  | | |
| LC-LS | | miR169i | | 86.9271 | 60.7997 | | -0.881666448 | | | 5.92E-14 |  | | |
| LC-LS | | miR171 | | 52.4922 | 47.9445 | | -0.223497643 | | | 0.1138494 |  | | |
| LC-LS | | miR172a | | 562.2967 | 382.1003 | | -0.952847728 | | | 2.19E-93 |  | | |
| LC-LS | | miR172e-3p | | 41.9098 | 58.6976 | | 0.830846216 | | | 5.32E-09 |  | | |
| LC-LS | | miR-1777 | | 17.3854 | 12.3702 | | -0.839382746 | | | 0.0013364 |  | | |
| LC-LS | | miR-1836 | | 6.887 | 6.5489 | | -0.124149975 | | | 0.7473426 |  | | |
| LC-LS | | miR-184-3p | | 1.0079 | 0.9702 | | -0.09402042 | | | 0.9234295 |  | | |
| LC-LS | | miR-185-3p | | 4.4513 | 5.3361 | | 0.447138013 | | | 0.3278842 |  | | |
| LC-LS | | miR-185-5p | | 10.7504 | 9.6212 | | -0.273695476 | | | 0.383553 |  | | |
| LC-LS | | miR1858a | | 111.7874 | 82.1443 | | -0.759920866 | | | 1.07E-13 |  | | |
| LC-LS | | miR1875 | | 1.4278 | 1.6979 | | 0.427305305 | | | 0.6029523 |  | | |
| LC-LS | | miR-188 | | 679.3753 | 763.5539 | | 0.288088946 | | | 1.17E-14 |  | | |
| LC-LS | | miR-190-5p | | 1.9317 | 1.0511 | | -1.500901543 | | | 0.0773562 |  | | |
| LC-LS | | miR-191-5p | | 9.1546 | 7.6 | | -0.458999388 | | | 0.1860612 |  | | |
| LC-LS | | miR-199a-3p | | 2.6876 | 2.7489 | | 0.055620642 | | | 0.9301136 |  | | |
| LC-LS | | miR-19b-5p | | 1.4278 | 1.7787 | | 0.541965168 | | | 0.5035271 |  | | |
| LC-LS | | miR-1b | | 13.9419 | 14.068 | | 0.022206654 | | | 0.9350712 |  | | |
| LC-LS | | miR-204-3p | | 4.7873 | 5.0936 | | 0.152955873 | | | 0.7372754 |  | | |
| LC-LS | | miR2089-3p | | 116.0708 | 164.2886 | | 0.856853729 | | | 9.36E-24 |  | | |
| LC-LS | | miR-208a-3p | | 99.1053 | 110.1995 | | 0.261698074 | | | 0.0075814 |  | | |
| LC-LS | | miR-210-5p | | 24.0204 | 31.1275 | | 0.639236771 | | | 0.0008593 |  | | |
| LC-LS | | miR2118-5p | | 507.5368 | 440.3937 | | -0.349968758 | | | 2.94E-14 |  | | |
| LC-LS | | miR-2127 | | 2.2677 | 2.7489 | | 0.47460244 | | | 0.4602425 |  | | |
| LC-LS | | miR-2129 | | 2.3517 | 3.5574 | | 1.020781963 | | | 0.0862299 |  | | |
| LC-LS | | miR-215 | | 2.1837 | 2.0213 | | -0.190595482 | | | 0.7822777 |  | | |
| LC-LS | | miR-219-3p | | 521.1428 | 659.499 | | 0.580706848 | | | 9.40E-45 |  | | |
| LC-LS | | miR-221 | | 2.5196 | 3.1532 | | 0.553235395 | | | 0.3594909 |  | | |
| LC-LS | | miR-2211-3p | | 39.5581 | 50.6125 | | 0.60776648 | | | 5.00E-05 |  | | |
| LC-LS | | miR-221-3p | | 1.4278 | 2.0213 | | 0.857302048 | | | 0.2730973 |  | | |
| LC-LS | | miR-222b-5p | | 0.9239 | 1.2128 | | 0.671039681 | | | 0.5034342 |  | | |
| LC-LS | | miR-2230-5p | | 32.0832 | 41.153 | | 0.614021513 | | | 0.0002228 |  | | |
| LC-LS | | miR-223-3p | | 2.1837 | 2.2638 | | 0.088846447 | | | 0.8985225 |  | | |
| LC-LS | | miR-2238g-3p | | 2.8556 | 3.0723 | | 0.180396504 | | | 0.7606507 |  | | |
| LC-LS | | miR-2265 | | 30.0675 | 25.0637 | | -0.448927085 | | | 0.0188422 |  | | |
| LC-LS | | miR-228 | | 158.2326 | 217.0841 | | 0.77989136 | | | 3.10E-26 |  | | |
| LC-LS | | miR-2292 | | 20.325 | 18.7574 | | -0.197953698 | | | 0.3822429 |  | | |
| LC-LS | | miR-2374 | | 2.5196 | 2.2638 | | -0.264030959 | | | 0.6841358 |  | | |
| LC-LS | | miR-23a-3p | | 1.6798 | 2.2638 | | 0.735871153 | | | 0.312575 |  | | |
| LC-LS | | miR-24 | | 1.7637 | 1.6979 | | -0.093773002 | | | 0.9001064 |  | | |
| LC-LS | | miR-243-3p | | 1.7637 | 1.4553 | | -0.474027993 | | | 0.5520976 |  | | |
| LC-LS | | miR-24-3p | | 1.7637 | 1.6979 | | -0.093773002 | | | 0.9001064 |  | | |
| LC-LS | | miR-2442 | | 1.8477 | 1.8596 | | 0.015833152 | | | 0.9861182 |  | | |
| LC-LS | | miR-2448-5p | | 5.8791 | 6.9532 | | 0.413841519 | | | 0.2989159 |  | | |
| LC-LS | | miR-25-3p | | 3.0236 | 3.234 | | 0.165912139 | | | 0.7733869 |  | | |
| LC-LS | | miR-254-3p | | 104.6485 | 107.774 | | 0.072581644 | | | 0.4555004 |  | | |
| LC-LS | | miR-2553-5p | | 1.0079 | 1.2128 | | 0.456420967 | | | 0.6424924 |  | | |
| LC-LS | | miR-2576 | | 11.0864 | 14.2297 | | 0.615619428 | | | 0.0298197 |  | | |
| LC-LS | | miR-26b | | 7.7269 | 7.2766 | | -0.148086792 | | | 0.6850454 |  | | |
| LC-LS | | miR-270 | | 13582.383 | 10000.502 | | -0.755029971 | | | 0 |  | | |
| LC-LS | | miR-2717 | | 2.7716 | 2.4255 | | -0.328973019 | | | 0.5979141 |  | | |
| LC-LS | | miR-2733e | | 18.3093 | 24.0935 | | 0.677081607 | | | 0.0019819 |  | | |
| LC-LS | | miR-2738 | | 6.887 | 7.5191 | | 0.216568258 | | | 0.5644162 |  | | |
| LC-LS | | miR-2756 | | 2.1837 | 1.4553 | | -1.000847198 | | | 0.1863249 |  | | |
| LC-LS | | miR-2828 | | 9.6586 | 12.9361 | | 0.720587627 | | | 0.0164743 |  | | |
| LC-LS | | miR-2840 | | 6.0471 | 6.6298 | | 0.226889604 | | | 0.5715005 |  | | |
| LC-LS | | miR2916 | | 111.1995 | 80.2847 | | -0.803390507 | | | 6.12E-15 |  | | |
| LC-LS | | miR2936 | | 6.971 | 8.1659 | | 0.390189576 | | | 0.2869362 |  | | |
| LC-LS | | miR-294-5p | | 4.3674 | 5.1744 | | 0.41817537 | | | 0.3662665 |  | | |
| LC-LS | | miR-2962-5p | | 4.2834 | 2.9915 | | -0.885334255 | | | 0.0958355 |  | | |
| LC-LS | | miR-2966 | | 3.2755 | 3.4766 | | 0.146952907 | | | 0.7911405 |  | | |
| LC-LS | | miR-2988 | | 35.1908 | 27.9743 | | -0.566012829 | | | 0.001546 |  | | |
| LC-LS | | miR-29a-3p | | 3.6955 | 4.2042 | | 0.318074628 | | | 0.532473 |  | | |
| LC-LS | | miR-3125 | | 939.3168 | 1127.6245 | | 0.450632322 | | | 3.22E-47 |  | | |
| LC-LS | | miR-3156-5p | | 1.9317 | 1.4553 | | -0.69842848 | | | 0.3708947 |  | | |
| LC-LS | | miR-316-3p | | 4.1154 | 3.8808 | | -0.144758989 | | | 0.771957 |  | | |
| LC-LS | | miR-320a | | 10.7504 | 10.9957 | | 0.055643071 | | | 0.8561712 |  | | |
| LC-LS | | miR-330-3p | | 7.5589 | 5.2553 | | -0.896473157 | | | 0.0249306 |  | | |
| LC-LS | | miR-3319 | | 289.925 | 256.0541 | | -0.306397555 | | | 4.38E-07 |  | | |
| LC-LS | | miR-3351 | | 2.2677 | 2.4255 | | 0.165912139 | | | 0.8042089 |  | | |
| LC-LS | | miR-342 | | 1.5958 | 1.3745 | | -0.368182473 | | | 0.6565793 |  | | |
| LC-LS | | miR-342-3p | | 1.5958 | 1.3745 | | -0.368182473 | | | 0.6565793 |  | | |
| LC-LS | | miR-344d-1-5p | | 4.2834 | 2.9106 | | -0.952949667 | | | 0.0750721 |  | | |
| LC-LS | | miR-345-3p | | 19.4851 | 21.4254 | | 0.234118939 | | | 0.2917292 |  | | |
| LC-LS | | miR-347 | | 51.2324 | 50.1274 | | -0.053776216 | | | 0.7018213 |  | | |
| LC-LS | | miR-3488 | | 44.2614 | 49.804 | | 0.290980242 | | | 0.0466978 |  | | |
| LC-LS | | miR3522a | | 236.509 | 204.7139 | | -0.356067303 | | | 1.32E-07 |  | | |
| LC-LS | | miR-3532-3p | | 12.0942 | 17.9489 | | 0.973703595 | | | 0.000197 |  | | |
| LC-LS | | miR-35h | | 30.3195 | 31.6935 | | 0.109307913 | | | 0.5442963 |  | | |
| LC-LS | | miR3636-3p | | 11.9262 | 12.451 | | 0.106207116 | | | 0.7128644 |  | | |
| LC-LS | | miR-3742 | | 7.3069 | 8.651 | | 0.416449686 | | | 0.2431926 |  | | |
| LC-LS | | miR-375 | | 0.7559 | 1.2128 | | 1.16601382 | | | 0.268741 |  | | |
| LC-LS | | miR-3772 | | 67.9459 | 69.0465 | | 0.039629532 | | | 0.7438307 |  | | |
| LC-LS | | miR-3776 | | 9.7426 | 7.1149 | | -0.775200717 | | | 0.0257262 |  | | |
| LC-LS | | miR-378c | | 65.5943 | 56.1104 | | -0.385156757 | | | 0.0027336 |  | | |
| LC-LS | | miR-3816-5p | | 4.4513 | 4.9319 | | 0.252865457 | | | 0.5882833 |  | | |
| LC-LS | | miR-3825-3p | | 5.5432 | 5.8212 | | 0.120687258 | | | 0.7764489 |  | | |
| LC-LS | | miR-3870-5p | | 59.7991 | 39.9402 | | -0.995418013 | | | 3.62E-12 |  | | |
| LC-LS | | miR390a-5p | | 538.1083 | 379.7557 | | -0.859584913 | | | 1.68E-74 |  | | |
| LC-LS | | miR393-5p | | 22.2567 | 19.6467 | | -0.307631155 | | | 0.1601185 |  | | |
| LC-LS | | miR3946 | | 10237.999 | 8114.7407 | | -0.573227958 | | | 0 |  | | |
| LC-LS | | miR-3961 | | 16.9655 | 19.3233 | | 0.320939462 | | | 0.1736562 |  | | |
| LC-LS | | miR396b-3p | | 27.044 | 22.719 | | -0.4297869 | | | 0.0326409 |  | | |
| LC-LS | | miR396e-5p | | 147.4821 | 112.1399 | | -0.675667551 | | | 1.94E-14 |  | | |
| LC-LS | | miR397-3p | | 17.8893 | 15.2808 | | -0.38870331 | | | 0.1146001 |  | | |
| LC-LS | | miR397-5p | | 6.3831 | 5.983 | | -0.159648165 | | | 0.6914235 |  | | |
| LC-LS | | miR398a-3p | | 3.7794 | 3.7191 | | -0.039666953 | | | 0.9371319 |  | | |
| LC-LS | | miR-4006b-5p | | 1478.2649 | 1646.9285 | | 0.266466824 | | | 7.80E-26 |  | | |
| LC-LS | | miR-40-5p | | 1.2598 | 1.6979 | | 0.736041677 | | | 0.384308 |  | | |
| LC-LS | | miR-4063-3p | | 1.5118 | 1.2128 | | -0.543497471 | | | 0.5318066 |  | | |
| LC-LS | | miR-4089-5p | | 20.6609 | 17.868 | | -0.358185251 | | | 0.1169816 |  | | |
| LC-LS | | miR408b-5p | | 316.5491 | 418.6448 | | 0.689441476 | | | 2.22E-39 |  | | |
| LC-LS | | miR-4111-3p | | 2.2677 | 1.2936 | | -1.384427564 | | | 0.0734215 |  | | |
| LC-LS | | miR-4144-5p | | 104.5645 | 118.2846 | | 0.304069269 | | | 0.0013715 |  | | |
| LC-LS | | miR-4175-3p | | 8664.912 | 8913.3838 | | 0.069727769 | | | 6.68E-11 |  | | |
| LC-LS | | miR-423-5p | | 8.5667 | 8.651 | | 0.024150847 | | | 0.9451405 |  | | |
| LC-LS | | miR4342 | | 2.6036 | 1.9404 | | -0.725095591 | | | 0.280986 |  | | |
| LC-LS | | miR4402 | | 5.4592 | 4.5276 | | -0.461470692 | | | 0.3052684 |  | | |
| LC-LS | | miR444b.2 | | 160.5002 | 116.5867 | | -0.788378536 | | | 3.20E-20 |  | | |
| LC-LS | | miR-4451 | | 174.1902 | 214.7394 | | 0.516141564 | | | 7.79E-13 |  | | |
| LC-LS | | miR-4459 | | 40.1461 | 32.2594 | | -0.539419603 | | | 0.0012316 |  | | |
| LC-LS | | miR-4492 | | 211.4807 | 243.9265 | | 0.352023403 | | | 1.18E-07 |  | | |
| LC-LS | | miR-449c | | 5.2072 | 3.4766 | | -0.996355714 | | | 0.0408653 |  | | |
| LC-LS | | miR-4505 | | 17.2174 | 21.7488 | | 0.576222407 | | | 0.0115308 |  | | |
| LC-LS | | miR-4513 | | 27.212 | 26.4382 | | -0.071148219 | | | 0.7123745 |  | | |
| LC-LS | | miR-451a | | 5.7951 | 8.4085 | | 0.918033035 | | | 0.0159238 |  | | |
| LC-LS | | miR-4528 | | 3.6115 | 4.5276 | | 0.55755427 | | | 0.2668902 |  | | |
| LC-LS | | miR-4547-5p | | 21.7528 | 31.936 | | 0.947039356 | | | 1.25E-06 |  | | |
| LC-LS | | miR-4555 | | 9.8265 | 13.017 | | 0.693458879 | | | 0.0202604 |  | | |
| LC-LS | | miR-4577 | | 254.8182 | 254.0329 | | -0.007612402 | | | 0.9032253 |  | | |
| LC-LS | | miR-4578 | | 89.4467 | 80.689 | | -0.254129538 | | | 0.0193079 |  | | |
| LC-LS | | miR-4630 | | 14.7818 | 15.1191 | | 0.055645105 | | | 0.8312009 |  | | |
| LC-LS | | miR-4644 | | 4.1994 | 3.1532 | | -0.706654751 | | | 0.1800109 |  | | |
| LC-LS | | miR-466i-3p | | 4.7033 | 4.4468 | | -0.138309447 | | | 0.7670126 |  | | |
| LC-LS | | miR-4690-5p | | 16.7975 | 12.0468 | | -0.819875735 | | | 0.0020285 |  | | |
| LC-LS | | miR-4699-5p | | 5.8791 | 8.0851 | | 0.785811568 | | | 0.0403208 |  | | |
| LC-LS | | miR-4717-3p | | 13.1021 | 13.6638 | | 0.1035292 | | | 0.7068492 |  | | |
| LC-LS | | miR-4738-3p | | 2.5196 | 2.3447 | | -0.177432519 | | | 0.7818155 |  | | |
| LC-LS | | miR-4743-5p | | 5.2072 | 4.1234 | | -0.575546979 | | | 0.2172756 |  | | |
| LC-LS | | miR-4764-5p | | 143.5347 | 161.378 | | 0.288983242 | | | 0.0003726 |  | | |
| LC-LS | | miR-4825-5p | | 13.774 | 12.8553 | | -0.170240485 | | | 0.5348969 |  | | |
| LC-LS | | miR-483-5p | | 35.6107 | 26.7616 | | -0.704568959 | | | 9.26E-05 |  | | |
| LC-LS | | miR-4865-3p | | 4.4513 | 4.9319 | | 0.252865457 | | | 0.5882833 |  | | |
| LC-LS | | miR-486-5p | | 0.8399 | 1.3745 | | 1.21480852 | | | 0.2206981 |  | | |
| LC-LS | | miR-489 | | 36.7026 | 35.4935 | | -0.08261627 | | | 0.6197264 |  | | |
| LC-LS | | miR-4898 | | 8.3148 | 8.2468 | | -0.02025282 | | | 0.9522993 |  | | |
| LC-LS | | miR-4962-3p | | 8.8187 | 7.6808 | | -0.340721687 | | | 0.329402 |  | | |
| LC-LS | | miR4995 | | 11.2543 | 9.3787 | | -0.449629608 | | | 0.150439 |  | | |
| LC-LS | | miR5021 | | 29.1437 | 31.6126 | | 0.200552428 | | | 0.2706704 |  | | |
| LC-LS | | miR5049c | | 9.3226 | 10.7531 | | 0.352071047 | | | 0.2678449 |  | | |
| LC-LS | | miR5054 | | 66.2662 | 77.2933 | | 0.379631463 | | | 0.0013512 |  | | |
| LC-LS | | miR5059 | | 43.8415 | 42.5275 | | -0.075049443 | | | 0.6219739 |  | | |
| LC-LS | | miR5068 | | 49.8886 | 55.4636 | | 0.261266457 | | | 0.0586929 |  | | |
| LC-LS | | miR5072 | | 41.7418 | 39.4551 | | -0.138950821 | | | 0.3765019 |  | | |
| LC-LS | | miR5078 | | 5152.5525 | 6043.993 | | 0.393554612 | | | 2.18E-189 |  | | |
| LC-LS | | miR5084 | | 7.8948 | 8.4893 | | 0.179059136 | | | 0.6112481 |  | | |
| LC-LS | | miR-5106 | | 16.2096 | 14.2297 | | -0.321291091 | | | 0.2112866 |  | | |
| LC-LS | | miR-511-3p | | 2.4356 | 2.5064 | | 0.070670274 | | | 0.9151803 |  | | |
| LC-LS | | miR-5124a | | 78.6124 | 63.1444 | | -0.540380109 | | | 5.89E-06 |  | | |
| LC-LS | | miR5139 | | 23.0966 | 21.9914 | | -0.120932607 | | | 0.5660616 |  | | |
| LC-LS | | miR5156 | | 2.3517 | 3.1532 | | 0.723315853 | | | 0.2387695 |  | | |
| LC-LS | | miR5169a | | 5.2912 | 5.7404 | | 0.200963856 | | | 0.6406078 |  | | |
| LC-LS | | miR-5187-5p | | 32.1672 | 29.4296 | | -0.219368643 | | | 0.2242345 |  | | |
| LC-LS | | miR-5192 | | 76.3447 | 58.7784 | | -0.644899322 | | | 1.35E-07 |  | | |
| LC-LS | | miR5198 | | 13.186 | 15.4425 | | 0.389596495 | | | 0.1427776 |  | | |
| LC-LS | | miR5205b | | 11.5903 | 11.1574 | | -0.093881283 | | | 0.7512157 |  | | |
| LC-LS | | miR5211 | | 2.1837 | 2.9915 | | 0.776279487 | | | 0.2208975 |  | | |
| LC-LS | | miR5217 | | 14.8658 | 18.5957 | | 0.552124195 | | | 0.024895 |  | | |
| LC-LS | | miR5291a | | 5.3752 | 6.5489 | | 0.487098181 | | | 0.2389317 |  | | |
| LC-LS | | miR5293 | | 2.0997 | 2.9915 | | 0.873023227 | | | 0.1727975 |  | | |
| LC-LS | | miR529-5p | | 21.5848 | 17.302 | | -0.54546558 | | | 0.0166783 |  | | |
| LC-LS | | miR5298b | | 17.4694 | 15.685 | | -0.265734179 | | | 0.2803232 |  | | |
| LC-LS | | miR-5348-5p | | 6.719 | 7.9234 | | 0.406646767 | | | 0.2752022 |  | | |
| LC-LS | | miR-5359-5p | | 2.3517 | 2.7489 | | 0.384897134 | | | 0.5458743 |  | | |
| LC-LS | | miR5368 | | 11.5903 | 13.9872 | | 0.463601136 | | | 0.0995548 |  | | |
| LC-LS | | miR5483 | | 3.7794 | 5.417 | | 0.887812243 | | | 0.0611234 |  | | |
| LC-LS | | miR5498 | | 17.0495 | 19.6467 | | 0.349693493 | | | 0.1360739 |  | | |
| LC-LS | | miR5565c | | 14.0259 | 13.5829 | | -0.079153467 | | | 0.7683195 |  | | |
| LC-LS | | miR5568f-5p | | 2.4356 | 2.3447 | | -0.0938075 | | | 0.8832851 |  | | |
| LC-LS | | miR-5695 | | 15.2857 | 18.1106 | | 0.418234878 | | | 0.0892238 |  | | |
| LC-LS | | miR-5703 | | 94.738 | 117.476 | | 0.530548419 | | | 5.37E-08 |  | | |
| LC-LS | | miR-5709-3p | | 18.6452 | 19.2425 | | 0.077769036 | | | 0.7366087 |  | | |
| LC-LS | | miR5718 | | 106.8322 | 110.1995 | | 0.076536752 | | | 0.4263341 |  | | |
| LC-LS | | miR5721 | | 687.2702 | 681.4096 | | -0.021121234 | | | 0.580963 |  | | |
| LC-LS | | miR5773 | | 30.3195 | 30.5616 | | 0.019615121 | | | 0.9147591 |  | | |
| LC-LS | | miR5783 | | 2.6876 | 3.4766 | | 0.634841805 | | | 0.2728793 |  | | |
| LC-LS | | miR-5866 | | 15.9576 | 12.5319 | | -0.596001504 | | | 0.0253003 |  | | |
| LC-LS | | miR-5991 | | 4.6193 | 5.2553 | | 0.318138137 | | | 0.4843398 |  | | |
| LC-LS | | miR-601 | | 56.4396 | 42.1232 | | -0.721572271 | | | 4.84E-07 |  | | |
| LC-LS | | miR-61 | | 4.1994 | 4.2042 | | 0.002817428 | | | 0.9973652 |  | | |
| LC-LS | | miR6108f | | 49.0487 | 63.3061 | | 0.629321742 | | | 2.77E-06 |  | | |
| LC-LS | | miR6170 | | 3.3595 | 4.5276 | | 0.735944577 | | | 0.1500374 |  | | |
| LC-LS | | miR6206 | | 5.4592 | 3.8808 | | -0.841653045 | | | 0.0722872 |  | | |
| LC-LS | | miR6214 | | 6.887 | 4.9319 | | -0.823526516 | | | 0.047645 |  | | |
| LC-LS | | miR6221-5p | | 5.6272 | 4.851 | | -0.366066303 | | | 0.4041849 |  | | |
| LC-LS | | miR6246 | | 56.0197 | 61.9316 | | 0.24743716 | | | 0.0581239 |  | | |
| LC-LS | | miR6250 | | 60.2191 | 52.3104 | | -0.347241643 | | | 0.0093731 |  | | |
| LC-LS | | miR6251 | | 2.3517 | 2.2638 | | -0.093950518 | | | 0.8852495 |  | | |
| LC-LS | | miR6263 | | 1.1758 | 1.7787 | | 1.020886842 | | | 0.2296616 |  | | |
| LC-LS | | miR6300 | | 435.4755 | 375.7131 | | -0.364057764 | | | 2.60E-13 |  | | |
| LC-LS | | miR-6308-3p | | 1.4278 | 1.3745 | | -0.093829946 | | | 0.9097133 |  | | |
| LC-LS | | miR-6314 | | 4.8713 | 5.5787 | | 0.334418257 | | | 0.4492583 |  | | |
| LC-LS | | miR-6385 | | 11.3383 | 9.0553 | | -0.554514178 | | | 0.0782553 |  | | |
| LC-LS | | miR6478 | | 27.4639 | 26.9233 | | -0.049030938 | | | 0.7978479 |  | | |
| LC-LS | | miR-6510-5p | | 8.9027 | 7.4383 | | -0.44322501 | | | 0.2072524 |  | | |
| LC-LS | | miR-6534 | | 162.5999 | 232.9308 | | 0.886509903 | | | 5.38E-35 |  | | |
| LC-LS | | miR-65-5p | | 9.5746 | 10.9957 | | 0.341313041 | | | 0.276829 |  | | |
| LC-LS | | miR-6607-5p | | 1.8477 | 2.5064 | | 0.751990323 | | | 0.2776945 |  | | |
| LC-LS | | miR-661 | | 18.5612 | 23.0424 | | 0.533369232 | | | 0.0156241 |  | | |
| LC-LS | | miR-6633-5p | | 12.0942 | 12.0468 | | -0.009685014 | | | 0.9720344 |  | | |
| LC-LS | | miR-6640-5p | | 1.7637 | 2.4255 | | 0.785823022 | | | 0.2662956 |  | | |
| LC-LS | | miR-664-1-5p | | 19.9051 | 19.0808 | | -0.104308327 | | | 0.6451625 |  | | |
| LC-LS | | miR-6653-3p | | 2.4356 | 1.9404 | | -0.560588293 | | | 0.4120762 |  | | |
| LC-LS | | miR-668 | | 7.8948 | 5.7404 | | -0.785950295 | | | 0.0421182 |  | | |
| LC-LS | | miR-669c-5p | | 1.0918 | 1.3745 | | 0.567896745 | | | 0.5413707 |  | | |
| LC-LS | | miR-6738-5p | | 1.1758 | 1.6979 | | 0.906226996 | | | 0.2923561 |  | | |
| LC-LS | | miR-6763-5p | | 516.4395 | 523.1039 | | 0.031622848 | | | 0.471695 |  | | |
| LC-LS | | miR-6791-5p | | 87.935 | 100.3357 | | 0.325363779 | | | 0.0016484 |  | | |
| LC-LS | | miR-6797-5p | | 3.5275 | 2.3447 | | -1.007317117 | | | 0.089865 |  | | |
| LC-LS | | miR-6810-5p | | 9.0707 | 7.8425 | | -0.358826882 | | | 0.2984688 |  | | |
| LC-LS | | miR-6867-5p | | 16.9655 | 15.2808 | | -0.257937679 | | | 0.3013449 |  | | |
| LC-LS | | miR-6868-5p | | 172.2585 | 168.412 | | -0.055696271 | | | 0.4676927 |  | | |
| LC-LS | | miR-6883-5p | | 8.3988 | 11.7233 | | 0.822486244 | | | 0.0099279 |  | | |
| LC-LS | | miR-6899-5p | | 126.1493 | 130.4122 | | 0.081965322 | | | 0.3542556 |  | | |
| LC-LS | | miR-6906-5p | | 57.0275 | 70.9869 | | 0.540026104 | | | 1.72E-05 |  | | |
| LC-LS | | miR-6921-5p | | 3.0236 | 3.3957 | | 0.286243494 | | | 0.6135169 |  | | |
| LC-LS | | miR-6923-5p | | 13.522 | 14.068 | | 0.09762795 | | | 0.7187962 |  | | |
| LC-LS | | miR-6929-5p | | 3.1075 | 3.5574 | | 0.333472333 | | | 0.5485378 |  | | |
| LC-LS | | miR-6934-5p | | 46.6131 | 56.5955 | | 0.4785809 | | | 0.0006217 |  | | |
| LC-LS | | miR-6949-5p | | 2.5196 | 2.2638 | | -0.264030959 | | | 0.6841358 |  | | |
| LC-LS | | miR-6960-5p | | 168.479 | 162.429 | | -0.090193029 | | | 0.2465333 |  | | |
| LC-LS | | miR-6963-5p | | 10.8344 | 11.8042 | | 0.211434083 | | | 0.4795922 |  | | |
| LC-LS | | miR-6996-3p | | 286.4815 | 274.6498 | | -0.104021608 | | | 0.0818316 |  | | |
| LC-LS | | miR-6999-5p | | 45.5213 | 46.57 | | 0.056173037 | | | 0.7041684 |  | | |
| LC-LS | | miR-7 | | 13.606 | 14.2297 | | 0.110540846 | | | 0.6820266 |  | | |
| LC-LS | | miR-7002-5p | | 455.8844 | 628.6949 | | 0.792686744 | | | 7.33E-75 |  | | |
| LC-LS | | miR-7003-5p | | 52.6602 | 68.7231 | | 0.656592721 | | | 3.75E-07 |  | | |
| LC-LS | | miR-7011-5p | | 4.7873 | 4.6893 | | -0.051011202 | | | 0.9103197 |  | | |
| LC-LS | | miR-7014-5p | | 43.0856 | 44.0636 | | 0.055356694 | | | 0.7159111 |  | | |
| LC-LS | | miR-7028-3p | | 18.9812 | 12.6936 | | -0.992319883 | | | 9.53E-05 |  | | |
| LC-LS | | miR-7033-5p | | 14.8658 | 10.5106 | | -0.855015628 | | | 0.0025589 |  | | |
| LC-LS | | miR-7080-5p | | 11.8422 | 10.7531 | | -0.237937457 | | | 0.4247604 |  | | |
| LC-LS | | miR-7082-5p | | 97.7615 | 112.9484 | | 0.356134812 | | | 0.0002687 |  | | |
| LC-LS | | miR-7086-5p | | 13.354 | 10.1063 | | -0.687252652 | | | 0.0194368 |  | | |
| LC-LS | | miR-7170-5p | | 281.6943 | 293.8114 | | 0.103869906 | | | 0.0785974 |  | | |
| LC-LS | | miR-7225-5p | | 107.672 | 117.3952 | | 0.213227839 | | | 0.0240317 |  | | |
| LC-LS | | miR-7247-5p | | 151.9335 | 172.6162 | | 0.314768656 | | | 6.37E-05 |  | | |
| LC-LS | | miR-7398g-5p | | 2.8556 | 3.5574 | | 0.541965168 | | | 0.3390965 |  | | |
| LC-LS | | miR-7448-3p | | 138.6635 | 139.8717 | | 0.02139626 | | | 0.8012815 |  | | |
| LC-LS | | miR-7455-5p | | 26.6241 | 26.3573 | | -0.02483949 | | | 0.8977294 |  | | |
| LC-LS | | miR-745-5p | | 8.9027 | 10.3489 | | 0.371241883 | | | 0.2526835 |  | | |
| LC-LS | | miR-7472-5p | | 29.7316 | 26.1956 | | -0.312280872 | | | 0.0994763 |  | | |
| LC-LS | | miR7485 | | 1.9317 | 1.1319 | | -1.318246125 | | | 0.1138383 |  | | |
| LC-LS | | miR7494b | | 13.774 | 13.9872 | | 0.037882104 | | | 0.8891926 |  | | |
| LC-LS | | miR-761 | | 1.2598 | 1.2936 | | 0.065297981 | | | 0.9459285 |  | | |
| LC-LS | | miR-762 | | 29.6476 | 38.2424 | | 0.627831253 | | | 0.0002797 |  | | |
| LC-LS | | miR-7657-5p | | 2.5196 | 3.8808 | | 1.065297979 | | | 0.0624637 |  | | |
| LC-LS | | miR-7665-5p | | 1.6798 | 1.617 | | -0.093971477 | | | 0.9024178 |  | | |
| LC-LS | | miR7773-3p | | 52.7442 | 76.6464 | | 0.92177801 | | | 2.26E-13 |  | | |
| LC-LS | | miR-7847-3p | | 5.2072 | 4.3659 | | -0.434606885 | | | 0.3443541 |  | | |
| LC-LS | | miR-7865 | | 1363.286 | 1099.2459 | | -0.530929931 | | | 7.06E-77 |  | | |
| LC-LS | | miR-7907b-3p | | 44.1775 | 32.5828 | | -0.750819393 | | | 3.86E-06 |  | | |
| LC-LS | | miR-7927c-3p | | 5.6272 | 4.0425 | | -0.815726597 | | | 0.0761551 |  | | |
| LC-LS | | miR-7977 | | 75.0849 | 93.8676 | | 0.550641228 | | | 4.78E-07 |  | | |
| LC-LS | | miR8000 | | 47.285 | 40.587 | | -0.376716268 | | | 0.0127709 |  | | |
| LC-LS | | miR-8094 | | 6.887 | 8.2468 | | 0.444402401 | | | 0.2254031 |  | | |
| LC-LS | | miR-8117 | | 212.7405 | 291.5476 | | 0.77720664 | | | 1.87E-34 |  | | |
| LC-LS | | miR8134 | | 120.2702 | 130.8164 | | 0.207302519 | | | 0.0204798 |  | | |
| LC-LS | | miR8144 | | 50.1406 | 58.455 | | 0.378395486 | | | 0.0054708 |  | | |
| LC-LS | | miR8148 | | 1.8477 | 2.0213 | | 0.221472197 | | | 0.7642605 |  | | |
| LC-LS | | miR8154 | | 5.1232 | 4.851 | | -0.134646289 | | | 0.7633241 |  | | |
| LC-LS | | miR8155 | | 8.5667 | 10.5106 | | 0.504363202 | | | 0.1222289 |  | | |
| LC-LS | | miR815a | | 149.1619 | 115.2931 | | -0.635208085 | | | 3.69E-13 |  | | |
| LC-LS | | miR-81-5p | | 4.2834 | 5.5787 | | 0.651618795 | | | 0.153029 |  | | |
| LC-LS | | miR8175 | | 34.015 | 22.8807 | | -0.977908668 | | | 2.49E-07 |  | | |
| LC-LS | | miR8176 | | 1.4278 | 0.8085 | | -1.40260996 | | | 0.1536259 |  | | |
| LC-LS | | miR-8209-5p | | 32.5032 | 28.9445 | | -0.285988469 | | | 0.1137103 |  | | |
| LC-LS | | miR827 | | 105.4044 | 134.293 | | 0.597387028 | | | 7.87E-11 |  | | |
| LC-LS | | miR-8301-3p | | 9.7426 | 8.8936 | | -0.224867953 | | | 0.4932411 |  | | |
| LC-LS | | miR831-5p | | 3.4435 | 2.9106 | | -0.414657503 | | | 0.4627665 |  | | |
| LC-LS | | miR-8343-5p | | 334.6904 | 329.4665 | | -0.038798025 | | | 0.4799928 |  | | |
| LC-LS | | miR-8364f-3p | | 72.3133 | 49.9657 | | -0.911721605 | | | 1.65E-12 |  | | |
| LC-LS | | miR-8365-3p | | 5.8791 | 8.8127 | | 0.998335413 | | | 0.0077565 |  | | |
| LC-LS | | miR-8442-3p | | 36.1146 | 34.4424 | | -0.116924623 | | | 0.487723 |  | | |
| LC-LS | | miR845a | | 11.2543 | 12.6127 | | 0.281045058 | | | 0.3344624 |  | | |
| LC-LS | | miR847-5p | | 105.9923 | 88.2889 | | -0.450722037 | | | 9.52E-06 |  | | |
| LC-LS | | miR-8528a | | 26.4561 | 17.7063 | | -0.99038422 | | | 4.22E-06 |  | | |
| LC-LS | | miR854 | | 10.4145 | 11.5616 | | 0.257704536 | | | 0.3958559 |  | | |
| LC-LS | | miR8561.1 | | 2.0997 | 1.6979 | | -0.523848485 | | | 0.4753166 |  | | |
| LC-LS | | miR860 | | 1.6798 | 1.4553 | | -0.353822492 | | | 0.6605035 |  | | |
| LC-LS | | miR8660 | | 9.6586 | 8.2468 | | -0.38973382 | | | 0.2453741 |  | | |
| LC-LS | | miR8692 | | 3.3595 | 4.0425 | | 0.456441635 | | | 0.3861995 |  | | |
| LC-LS | | miR-8838 | | 1.6798 | 1.617 | | -0.093971477 | | | 0.9024178 |  | | |
| LC-LS | | miR-8896 | | 6.719 | 7.034 | | 0.112996645 | | | 0.7696177 |  | | |
| LC-LS | | miR894 | | 995.5045 | 1079.0332 | | 0.198712755 | | | 1.68E-10 |  | | |
| LC-LS | | miR-9277 | | 14.6978 | 15.7659 | | 0.173015022 | | | 0.5017989 |  | | |
| LC-LS | | miR-9334 | | 1.9317 | 1.7787 | | -0.203513644 | | | 0.781815 |  | | |
| LC-LS | | miR-9384-3p | | 15.2017 | 13.2595 | | -0.337126379 | | | 0.2047583 |  | | |
| LC-LS | | miR-9422 | | 60.975 | 48.5104 | | -0.564008427 | | | 3.26E-05 |  | | |
| LC-LS | | miR-9427 | | 5.9631 | 6.8723 | | 0.349989084 | | | 0.3795045 |  | | |
| LC-LS | | miR-944 | | 31.0754 | 23.4467 | | -0.694725011 | | | 0.0003133 |  | | |
| LC-LS | | miR-9458 | | 1.6798 | 2.3447 | | 0.822469593 | | | 0.2546103 |  | | |
| LC-LS | | miR-9465 | | 1.6798 | 1.8596 | | 0.250790196 | | | 0.7454398 |  | | |
| LC-LS | | miR9722 | | 28.6398 | 29.1871 | | 0.04668583 | | | 0.8029405 |  | | |
| LC-LS | | miR9738 | | 18.9812 | 15.3616 | | -0.521815859 | | | 0.0313694 |  | | |
| LC-LS | | miR-9795-3p | | 2.9396 | 2.3447 | | -0.557670807 | | | 0.3693428 |  | | |
| LC-LS | | miR-9820-5p | | 82.3078 | 60.7997 | | -0.746996516 | | | 3.47E-10 |  | | |
| LC-LS | | miR-9835-3p | | 14.6138 | 12.1276 | | -0.459922285 | | | 0.0939753 |  | | |
| LC-LS | | miR-9857-5p | | 1.0079 | 0.8894 | | -0.308478235 | | | 0.7660022 |  | | |
| LC-LS | | miR-9875 | | 44.2614 | 44.4679 | | 0.011479693 | | | 0.939799 |  | | |
| LC-LS | | miR-9894 | | 40.398 | 40.0211 | | -0.023117807 | | | 0.8830266 |  | | |
| LC-LS | | miR-H13 | | 160.5002 | 173.1822 | | 0.187559989 | | | 0.0156264 |  | | |
| LC-LS | | miR-H3 | | 5.7951 | 6.4681 | | 0.270971951 | | | 0.5062437 |  | | |
| LC-LS | | miR-H8 | | 7.055 | 5.8212 | | -0.474097912 | | | 0.2315337 |  | | |
| LC-LS | | miR-J1-5p | | 12.6821 | 12.3702 | | -0.061413903 | | | 0.8273093 |  | | |
| LC-LS | | miR-LLT11a | | 11.9262 | 17.0595 | | 0.882861601 | | | 0.0008965 |  | | |
| LC-LS | | miR-M95-1-3p | | 2.5196 | 1.7787 | | -0.858809769 | | | 0.2156486 |  | | |
| RC-RS | let-7 | | 10.8063 | | | 17.5139 | | 1.190892987 | 1.05E-05 | | | ** |  |
| RC-RS | let-7-5p | | 69.9434 | | | 177.5056 | | 2.296907423 | 1.72E-129 | | | ** |  |
| RC-RS | let-7c-5p | | 0.01 | | | 3.4712 | | 14.42706271 | 3.02E-13 | | | ** |  |
| RC-RS | let-7e | | 0.01 | | | 160.0706 | | 23.87575394 | 0 | | | ** |  |
| RC-RS | let-7f | | 131.6332 | | | 268.5463 | | 1.758484413 | 3.23E-128 | | | ** |  |
| RC-RS | let-7f-5p | | 138.7807 | | | 277.5399 | | 1.709320236 | 1.69E-126 | | | ** |  |
| RC-RS | let-7g | | 0.01 | | | 2.6034 | | 13.71755142 | 4.11E-10 | | | ** |  |
| RC-RS | let-7g-5p | | 10.1256 | | | 15.3838 | | 1.031526823 | 0.0002786 | | | ** |  |
| RC-RS | let-7i | | 2.5527 | | | 0.01 | | -13.66904745 | 2.82E-10 | | | ** |  |
| RC-RS | let-7k | | 1261.9596 | | | 0.01 | | -28.96818729 | 0 | | | ** |  |
| RC-RS | lin-4-3p | | 5.2755 | | | 0.01 | | -15.45939104 | 1.91E-20 | | | ** |  |
| RC-RS | miR1023c-3p | | 24.6759 | | | 0.01 | | -19.2642896 | 6.79E-93 | | | ** |  |
| RC-RS | miR1042-3p | | 10.1256 | | | 17.8295 | | 1.395403721 | 3.38E-07 | | | ** |  |
| RC-RS | miR1114 | | 0.01 | | | 6.3902 | | 15.93216192 | 8.67E-24 | | | ** |  |
| RC-RS | miR1135 | | 0.01 | | | 7.968 | | 16.47639606 | 1.74E-29 | | | ** |  |
| RC-RS | miR1169-3p | | 2.7229 | | | 0.01 | | -13.82823716 | 6.53E-11 | | | ** |  |
| RC-RS | miR-1175-5p | | 0.01 | | | 9.3881 | | 16.88089303 | 1.29E-34 | | | ** |  |
| RC-RS | miR-1188-5p | | 1.4465 | | | 0.01 | | -12.2681753 | 3.81E-06 | | | ** |  |
| RC-RS | miR1214 | | 0.01 | | | 508.6128 | | 26.72697845 | 0 | | | ** |  |
| RC-RS | miR-1224-5p | | 0.01 | | | 12.0704 | | 17.50070775 | 2.65E-44 | | | ** |  |
| RC-RS | miR-1231-5p | | 2.9781 | | | 0.01 | | -14.04918842 | 7.27E-12 | | | ** |  |
| RC-RS | miR-1253 | | 0.01 | | | 3.0768 | | 14.12960121 | 8.02E-12 | | | ** |  |
| RC-RS | miR-1260a | | 26.3777 | | | 3.8657 | | -4.736230419 | 3.73E-52 | | | ** |  |
| RC-RS | miR-1271b | | 3.829 | | | 8.4414 | | 1.949722638 | 3.81E-06 | | | ** |  |
| RC-RS | miR-1306 | | 27.1435 | | | 16.015 | | -1.301250865 | 2.60E-09 | | | ** |  |
| RC-RS | miR-1306-3p | | 0.01 | | | 61.5353 | | 21.51795803 | 6.03E-223 | | | ** |  |
| RC-RS | miR-132-3p | | 63.817 | | | 27.4542 | | -2.080328087 | 1.40E-41 | | | ** |  |
| RC-RS | miR-1351-3p | | 0.01 | | | 2031.2165 | | 30.14207657 | 0 | | | ** |  |
| RC-RS | miR-1407 | | 19.6556 | | | 0.01 | | -18.70329221 | 3.80E-74 | | | ** |  |
| RC-RS | miR-1408 | | 19.4855 | | | 9.6247 | | -1.73957727 | 1.10E-10 | | | ** |  |
| RC-RS | miR-1421j-5p | | 6.2115 | | | 1.8934 | | -2.930038213 | 5.75E-08 | | | ** |  |
| RC-RS | miR-1422m-3p | | 14.9757 | | | 0.01 | | -18.03262219 | 1.14E-56 | | | ** |  |
| RC-RS | miR-143 | | 18.8047 | | | 0.01 | | -18.59414526 | 5.72E-71 | | | ** |  |
| RC-RS | miR1432-3p | | 0.01 | | | 2.1301 | | 13.22268922 | 2.11E-08 | | | ** |  |
| RC-RS | miR1432-5p | | 39.4814 | | | 118.416 | | 2.708924022 | 9.48E-111 | | | ** |  |
| RC-RS | miR1436 | | 0.01 | | | 3.2345 | | 14.25287756 | 2.16E-12 | | | ** |  |
| RC-RS | miR-146a | | 17.2731 | | | 0.01 | | -18.38461651 | 3.00E-65 | | | ** |  |
| RC-RS | miR-1479 | | 37.4393 | | | 0.01 | | -20.2924765 | 1.47E-140 | | | ** |  |
| RC-RS | miR-149-3p | | 483.1371 | | | 175.2178 | | -2.501499324 | 0 | | | ** |  |
| RC-RS | miR-1498 | | 291.3458 | | | 917.349 | | 2.82879362 | 0 | | | ** |  |
| RC-RS | miR-150-5p | | 0.01 | | | 1.0256 | | 11.42008992 | 0.0002052 | | | ** |  |
| RC-RS | miR1526 | | 0.01 | | | 1.2623 | | 11.93223668 | 2.87E-05 | | | ** |  |
| RC-RS | miR-1556 | | 7.8282 | | | 0.01 | | -16.43274015 | 5.61E-30 | | | ** |  |
| RC-RS | miR-1560-5p | | 10.0405 | | | 0.01 | | -17.0465892 | 3.07E-38 | | | ** |  |
| RC-RS | miR-1564-5p | | 0.01 | | | 1.4989 | | 12.3559379 | 4.01E-06 | | | ** |  |
| RC-RS | miR-1576 | | 428.3396 | | | 0.01 | | -26.30333581 | 0 | | | ** |  |
| RC-RS | miR-1579 | | 135.0367 | | | 27.6909 | | -3.907716644 | 2.62E-208 | | | ** |  |
| RC-RS | miR157a-5p | | 2454.9969 | | | 0.01 | | -30.60943305 | 0 | | | ** |  |
| RC-RS | miR157d | | 0.01 | | | 13.4904 | | 17.77501532 | 1.97E-49 | | | ** |  |
| RC-RS | miR-1581 | | 0.01 | | | 2.0512 | | 13.12960121 | 4.06E-08 | | | ** |  |
| RC-RS | miR-1582 | | 9.3598 | | | 5.838 | | -1.164183156 | 0.0015366 | | | ** |  |
| RC-RS | miR159b-3p | | 8.9344 | | | 14.8316 | | 1.250048489 | 2.35E-05 | | | ** |  |
| RC-RS | miR160a-5p | | 10.6362 | | | 17.2772 | | 1.19646408 | 1.11E-05 | | | ** |  |
| RC-RS | miR-16-2-3p | | 0.01 | | | 1.6567 | | 12.60280541 | 1.08E-06 | | | ** |  |
| RC-RS | miR-1641 | | 0.01 | | | 54.9873 | | 21.24047732 | 2.68E-199 | | | ** |  |
| RC-RS | miR-1648-5p | | 19.1451 | | | 0.01 | | -18.63839025 | 3.07E-72 | | | ** |  |
| RC-RS | miR-1649-5p | | 291.6862 | | | 186.0259 | | -1.109325178 | 1.24E-64 | | | ** |  |
| RC-RS | miR165a-5p | | 2269.2469 | | | 996.556 | | -2.029516078 | 0 | | | ** |  |
| RC-RS | miR166 | | 0.01 | | | 9003.4006 | | 33.81432223 | 0 | | | ** |  |
| RC-RS | miR166d-5p | | 41.6087 | | | 0.01 | | -20.55288878 | 3.95E-156 | | | ** |  |
| RC-RS | miR-1677b-3p | | 10.2107 | | | 0.01 | | -17.08804586 | 7.11E-39 | | | ** |  |
| RC-RS | miR168a-3p | | 37.6095 | | | 69.8978 | | 1.528559384 | 8.05E-28 | | | ** |  |
| RC-RS | miR-1694 | | 18.8047 | | | 0.01 | | -18.59414526 | 5.72E-71 | | | ** |  |
| RC-RS | miR171a-5p | | 33.8655 | | | 67.2155 | | 1.690668477 | 1.99E-31 | | | ** |  |
| RC-RS | miR172e-3p | | 24.5908 | | | 0.01 | | -19.25576957 | 1.41E-92 | | | ** |  |
| RC-RS | miR-1759-5p | | 2.0421 | | | 0.01 | | -13.11863532 | 2.27E-08 | | | ** |  |
| RC-RS | miR-1770 | | 3.4887 | | | 0.8678 | | -3.431425173 | 6.55E-06 | | | ** |  |
| RC-RS | miR-1777 | | 8.9344 | | | 5.838 | | -1.049463238 | 0.0047683 | | | ** |  |
| RC-RS | miR-1788a-3p | | 0.01 | | | 3.7868 | | 14.64168286 | 2.19E-14 | | | ** |  |
| RC-RS | miR-1795 | | 133.5902 | | | 0.01 | | -23.42975208 | 0 | | | ** |  |
| RC-RS | miR-1797 | | 0.01 | | | 32.0299 | | 19.90760701 | 2.19E-116 | | | ** |  |
| RC-RS | miR-1819-3p | | 0.01 | | | 4.8913 | | 15.27290062 | 2.24E-18 | | | ** |  |
| RC-RS | miR-183-5p | | 4.2545 | | | 0.01 | | -14.92889843 | 1.24E-16 | | | ** |  |
| RC-RS | miR-1836 | | 0.01 | | | 7.4947 | | 16.325366 | 8.89E-28 | | | ** |  |
| RC-RS | miR-184 | | 0.01 | | | 2.2879 | | 13.39894469 | 5.67E-09 | | | ** |  |
| RC-RS | miR-1840 | | 0.01 | | | 145.1602 | | 23.63460617 | 0 | | | ** |  |
| RC-RS | miR-187-5p | | 0.01 | | | 1.7356 | | 12.71755142 | 5.60E-07 | | | ** |  |
| RC-RS | miR-193a | | 1378.4469 | | | 0.01 | | -29.18596041 | 0 | | | ** |  |
| RC-RS | miR-194-2-3p | | 94.1088 | | | 190.9172 | | 1.744634124 | 1.51E-90 | | | ** |  |
| RC-RS | miR-1957a | | 89.9394 | | | 38.7357 | | -2.077551037 | 9.06E-58 | | | ** |  |
| RC-RS | miR-1969 | | 1.0211 | | | 0.01 | | -11.40924477 | 0.0001479 | | | ** |  |
| RC-RS | miR-199a-3p | | 0.01 | | | 2.919 | | 13.99975279 | 2.98E-11 | | | ** |  |
| RC-RS | miR-199b-3p | | 3.4887 | | | 0.01 | | -14.43946533 | 9.02E-14 | | | ** |  |
| RC-RS | miR-19b-5p | | 0.01 | | | 1.1045 | | 11.60287985 | 0.0001065 | | | ** |  |
| RC-RS | miR-2013 | | 0.01 | | | 35.8167 | | 20.18320266 | 4.61E-130 | | | ** |  |
| RC-RS | miR-204-3p | | 5.701 | | | 0.01 | | -15.65069762 | 4.93E-22 | | | ** |  |
| RC-RS | miR-2075 | | 0.01 | | | 1.7356 | | 12.71755142 | 5.60E-07 | | | ** |  |
| RC-RS | miR2097-5p | | 0.01 | | | 10.8081 | | 17.22827907 | 9.60E-40 | | | ** |  |
| RC-RS | miR-21 | | 5.3606 | | | 0.01 | | -15.49885792 | 9.21E-21 | | | ** |  |
| RC-RS | miR-210-5p | | 23.3145 | | | 0.01 | | -19.12432309 | 8.24E-88 | | | ** |  |
| RC-RS | miR2118-5p | | 1140.1117 | | | 649.6706 | | -1.387088658 | 0 | | | ** |  |
| RC-RS | miR-2129 | | 3.2334 | | | 0.01 | | -14.2520387 | 8.10E-13 | | | ** |  |
| RC-RS | miR-2137 | | 63.3915 | | | 38.8146 | | -1.209804029 | 1.32E-17 | | | ** |  |
| RC-RS | miR-214-5p | | 0.01 | | | 11.597 | | 17.40203161 | 1.36E-42 | | | ** |  |
| RC-RS | miR-21-5p | | 0.01 | | | 4.9702 | | 15.31236633 | 1.16E-18 | | | ** |  |
| RC-RS | miR2199 | | 0.01 | | | 334.7362 | | 25.69521455 | 0 | | | ** |  |
| RC-RS | miR-21a-3p | | 51.2238 | | | 261.2094 | | 4.017899655 | 0 | | | ** |  |
| RC-RS | miR-2238g-3p | | 0.01 | | | 1.8145 | | 12.82719532 | 2.90E-07 | | | ** |  |
| RC-RS | miR-2238j-3p | | 28.8453 | | | 0.01 | | -19.64932907 | 1.83E-108 | | | ** |  |
| RC-RS | miR-22-3p | | 6.5519 | | | 0.01 | | -15.99379369 | 3.28E-25 | | | ** |  |
| RC-RS | miR-2253a | | 61.0941 | | | 172.141 | | 2.554840333 | 3.94E-147 | | | ** |  |
| RC-RS | miR-2265 | | 18.5495 | | | 11.5181 | | -1.175250634 | 6.76E-06 | | | ** |  |
| RC-RS | miR-228 | | 0.01 | | | 109.9746 | | 22.94998861 | 0 | | | ** |  |
| RC-RS | miR-2285aa | | 0.01 | | | 4.1812 | | 14.88603668 | 8.23E-16 | | | ** |  |
| RC-RS | miR-2290 | | 0.01 | | | 33.4499 | | 20.01459267 | 1.63E-121 | | | ** |  |
| RC-RS | miR-2292 | | 13.2739 | | | 8.2047 | | -1.186520194 | 0.0001239 | | | ** |  |
| RC-RS | miR-2303 | | 603.7938 | | | 200.6208 | | -2.717413079 | 0 | | | ** |  |
| RC-RS | miR-2348 | | 0.01 | | | 280.2222 | | 25.25680469 | 0 | | | ** |  |
| RC-RS | miR-2364 | | 1.2763 | | | 0.01 | | -11.95943956 | 1.65E-05 | | | ** |  |
| RC-RS | miR-2374 | | 1.3614 | | | 0.01 | | -12.11863528 | 7.93E-06 | | | ** |  |
| RC-RS | miR-2388-3p | | 933.5149 | | | 433.1926 | | -1.893564884 | 0 | | | ** |  |
| RC-RS | miR-239a-5p | | 11.6572 | | | 23.7463 | | 1.754781612 | 9.36E-13 | | | ** |  |
| RC-RS | miR-2413 | | 0.01 | | | 270.3608 | | 25.16844815 | 0 | | | ** |  |
| RC-RS | miR-2436-5p | | 0.01 | | | 1.42 | | 12.22257343 | 7.72E-06 | | | ** |  |
| RC-RS | miR-2461-3p | | 0.01 | | | 11.9126 | | 17.4682524 | 9.85E-44 | | | ** |  |
| RC-RS | miR-2462 | | 6.8922 | | | 0.01 | | -16.11867559 | 1.76E-26 | | | ** |  |
| RC-RS | miR-25 | | 29.1856 | | | 0.01 | | -19.67825475 | 9.78E-110 | | | ** |  |
| RC-RS | miR-252b | | 1.1913 | | | 0.01 | | -11.78946163 | 3.42E-05 | | | ** |  |
| RC-RS | miR-2551-3p | | 0.01 | | | 26.7442 | | 19.46280371 | 2.69E-97 | | | ** |  |
| RC-RS | miR-2553-5p | | 0.01 | | | 3.2345 | | 14.25287756 | 2.16E-12 | | | ** |  |
| RC-RS | miR2610a | | 20.8469 | | | 40.3924 | | 1.631302859 | 1.74E-18 | | | ** |  |
| RC-RS | miR-270 | | 568.4817 | | | 978.1743 | | 1.338509134 | 2.89E-292 | | | ** |  |
| RC-RS | miR-2717 | | 1.1913 | | | 0.01 | | -11.78946163 | 3.42E-05 | | | ** |  |
| RC-RS | miR-2733e | | 17.8688 | | | 10.9659 | | -1.204211431 | 6.38E-06 | | | ** |  |
| RC-RS | miR-2738 | | 2.6378 | | | 4.6546 | | 1.400640227 | 0.0093777 | | | ** |  |
| RC-RS | miR-2739 | | 0.01 | | | 11.4393 | | 17.36826386 | 5.05E-42 | | | ** |  |
| RC-RS | miR-2797d | | 69.1776 | | | 132.3009 | | 1.599155517 | 4.80E-55 | | | ** |  |
| RC-RS | miR-27b-5p | | 640.2971 | | | 58.6952 | | -5.893415451 | 0 | | | ** |  |
| RC-RS | miR2876-5p | | 0.01 | | | 125.2795 | | 23.27134256 | 0 | | | ** |  |
| RC-RS | miR2936 | | 5.1054 | | | 0.01 | | -15.37855874 | 8.27E-20 | | | ** |  |
| RC-RS | miR-294-5p | | 3.2334 | | | 0.01 | | -14.2520387 | 8.10E-13 | | | ** |  |
| RC-RS | miR2948-5p | | 0.01 | | | 19.8806 | | 18.73136409 | 1.65E-72 | | | ** |  |
| RC-RS | miR-3070-3p | | 1075.8693 | | | 0.01 | | -28.57472869 | 0 | | | ** |  |
| RC-RS | miR-3071-5p | | 5.5308 | | | 0.01 | | -15.57594606 | 2.13E-21 | | | ** |  |
| RC-RS | miR-3075 | | 27.654 | | | 0.01 | | -19.54530864 | 5.13E-104 | | | ** |  |
| RC-RS | miR-3125 | | 0.01 | | | 3.9446 | | 14.7423727 | 5.89E-15 | | | ** |  |
| RC-RS | miR-3126-5p | | 178.8577 | | | 91.514 | | -1.652666383 | 1.76E-78 | | | ** |  |
| RC-RS | miR-3154 | | 0.01 | | | 21.3796 | | 18.91064664 | 6.36E-78 | | | ** |  |
| RC-RS | miR-316-3p | | 2.8079 | | | 0.01 | | -13.9040498 | 3.14E-11 | | | ** |  |
| RC-RS | miR-3319 | | 429.1905 | | | 276.2776 | | -1.086393656 | 7.53E-91 | | | ** |  |
| RC-RS | miR-345-3p | | 28.3347 | | | 57.9852 | | 1.766121399 | 3.00E-29 | | | ** |  |
| RC-RS | miR3454f-3p | | 0.01 | | | 6.3113 | | 15.90152083 | 1.67E-23 | | | ** |  |
| RC-RS | miR-347 | | 72.9215 | | | 35.4222 | | -1.780781535 | 3.68E-37 | | | ** |  |
| RC-RS | miR3476-5p | | 49.8623 | | | 78.6547 | | 1.124146487 | 6.09E-19 | | | ** |  |
| RC-RS | miR-34a-3p | | 8.8493 | | | 0.01 | | -16.73512319 | 8.63E-34 | | | ** |  |
| RC-RS | miR-3506 | | 130.527 | | | 67.8466 | | -1.613778386 | 1.39E-55 | | | ** |  |
| RC-RS | miR3515 | | 0.01 | | | 7.968 | | 16.47639606 | 1.74E-29 | | | ** |  |
| RC-RS | miR-3526 | | 204.8951 | | | 317.8534 | | 1.082934443 | 5.00E-67 | | | ** |  |
| RC-RS | miR-3532-3p | | 4.2545 | | | 0.01 | | -14.92889843 | 1.24E-16 | | | ** |  |
| RC-RS | miR3636-3p | | 7.5729 | | | 0.01 | | -16.35096617 | 5.04E-29 | | | ** |  |
| RC-RS | miR-365a-1-5p | | 0.01 | | | 26.9809 | | 19.48453575 | 3.75E-98 | | | ** |  |
| RC-RS | miR-3689a-3p | | 240.9729 | | | 159.3606 | | -1.019853271 | 1.82E-46 | | | ** |  |
| RC-RS | miR-3741 | | 2.893 | | | 0.01 | | -13.97768662 | 1.51E-11 | | | ** |  |
| RC-RS | miR-3776 | | 3.9992 | | | 0.01 | | -14.77627644 | 1.12E-15 | | | ** |  |
| RC-RS | miR-3781 | | 0.01 | | | 1.8145 | | 12.82719532 | 2.90E-07 | | | ** |  |
| RC-RS | miR-378c | | 34.9717 | | | 0.01 | | -20.12431936 | 2.41E-131 | | | ** |  |
| RC-RS | miR-378i | | 0.01 | | | 3.1557 | | 14.19204862 | 4.16E-12 | | | ** |  |
| RC-RS | miR-379-5p | | 2.2123 | | | 0.01 | | -13.31607277 | 5.27E-09 | | | ** |  |
| RC-RS | miR-3816-5p | | 2.7229 | | | 0.01 | | -13.82823716 | 6.53E-11 | | | ** |  |
| RC-RS | miR-3828-5p | | 2922.3925 | | | 0.01 | | -31.03920499 | 0 | | | ** |  |
| RC-RS | miR-3935 | | 178.6876 | | | 392.5635 | | 1.941127027 | 1.44E-218 | | | ** |  |
| RC-RS | miR3946 | | 18.0389 | | | 2651.8551 | | 12.3080484 | 0 | | | ** |  |
| RC-RS | miR-3956 | | 1.0211 | | | 0.01 | | -11.40924477 | 0.0001479 | | | ** |  |
| RC-RS | miR395b-5p | | 0.01 | | | 53.0939 | | 21.15405754 | 1.85E-192 | | | ** |  |
| RC-RS | miR395e-5p | | 29.0155 | | | 0.01 | | -19.66383824 | 4.23E-109 | | | ** |  |
| RC-RS | miR396b-3p | | 8.0835 | | | 15.7783 | | 1.649489974 | 3.29E-08 | | | ** |  |
| RC-RS | miR397 | | 27.4838 | | | 0.01 | | -19.53008231 | 2.22E-103 | | | ** |  |
| RC-RS | miR3979-3p | | 0.01 | | | 44.1792 | | 20.70073135 | 2.90E-160 | | | ** |  |
| RC-RS | miR3979-5p | | 6.637 | | | 14.6738 | | 1.956773893 | 9.14E-10 | | | ** |  |
| RC-RS | miR398a-3p | | 0.01 | | | 1.3412 | | 12.0817669 | 1.49E-05 | | | ** |  |
| RC-RS | miR398a-5p | | 110.0205 | | | 204.6443 | | 1.530604302 | 2.58E-78 | | | ** |  |
| RC-RS | miR-4018a-5p | | 5.9563 | | | 13.2538 | | 1.972635389 | 4.75E-09 | | | ** |  |
| RC-RS | miR-4033-5p | | 0.01 | | | 18.5395 | | 18.55911551 | 1.15E-67 | | | ** |  |
| RC-RS | miR-4039-5p | | 0.01 | | | 3.0768 | | 14.12960121 | 8.02E-12 | | | ** |  |
| RC-RS | miR-40-5p | | 1.5316 | | | 0.01 | | -12.40916425 | 1.83E-06 | | | ** |  |
| RC-RS | miR-4063-3p | | 0.01 | | | 1.4989 | | 12.3559379 | 4.01E-06 | | | ** |  |
| RC-RS | miR-4080-5p | | 602.9429 | | | 0.01 | | -27.14658692 | 0 | | | ** |  |
| RC-RS | miR-4104-5p | | 0.01 | | | 13.9638 | | 17.86007798 | 3.85E-51 | | | ** |  |
| RC-RS | miR-4111-3p | | 0.01 | | | 1.42 | | 12.22257343 | 7.72E-06 | | | ** |  |
| RC-RS | miR-4127-3p | | 574.8634 | | | 369.4484 | | -1.090405452 | 8.20E-122 | | | ** |  |
| RC-RS | miR-4138-5p | | 0.01 | | | 3.9446 | | 14.7423727 | 5.89E-15 | | | ** |  |
| RC-RS | miR-4144-5p | | 1915.7008 | | | 3014.1243 | | 1.117792438 | 0 | | | ** |  |
| RC-RS | miR-4171-5p | | 12.7634 | | | 5.6802 | | -1.996707581 | 5.32E-09 | | | ** |  |
| RC-RS | miR-4185-5p | | 425.9571 | | | 0.01 | | -26.28958798 | 0 | | | ** |  |
| RC-RS | miR-4298 | | 5.6159 | | | 0.01 | | -15.61360501 | 1.03E-21 | | | ** |  |
| RC-RS | miR-4311 | | 0.01 | | | 10.3348 | | 17.11784044 | 4.92E-38 | | | ** |  |
| RC-RS | miR437 | | 0.01 | | | 1.1045 | | 11.60287985 | 0.0001065 | | | ** |  |
| RC-RS | miR4382 | | 0.01 | | | 2.5245 | | 13.64165029 | 7.92E-10 | | | ** |  |
| RC-RS | miR4387d | | 0.01 | | | 18.0661 | | 18.49532127 | 5.89E-66 | | | ** |  |
| RC-RS | miR4391 | | 0.01 | | | 46.3092 | | 20.81686109 | 5.88E-168 | | | ** |  |
| RC-RS | miR4400 | | 0.01 | | | 29.8998 | | 19.73788081 | 1.08E-108 | | | ** |  |
| RC-RS | miR4403 | | 0.01 | | | 4.8124 | | 15.23279311 | 4.33E-18 | | | ** |  |
| RC-RS | miR-4433a-3p | | 6.3817 | | | 2.5245 | | -2.287228854 | 4.62E-06 | | | ** |  |
| RC-RS | miR-4459 | | 1.872 | | | 0.01 | | -12.90413767 | 9.83E-08 | | | ** |  |
| RC-RS | miR-4496 | | 0.01 | | | 18.145 | | 18.5060689 | 3.06E-66 | | | ** |  |
| RC-RS | miR-449c | | 0.01 | | | 2.919 | | 13.99975279 | 2.98E-11 | | | ** |  |
| RC-RS | miR-4505 | | 14.4652 | | | 0.01 | | -17.9470831 | 9.19E-55 | | | ** |  |
| RC-RS | miR-4513 | | 190.0895 | | | 542.3783 | | 2.585842029 | 0 | | | ** |  |
| RC-RS | miR-4528 | | 0.01 | | | 2.9979 | | 14.06553145 | 1.55E-11 | | | ** |  |
| RC-RS | miR-4529-3p | | 0.01 | | | 29.1109 | | 19.67193404 | 7.61E-106 | | | ** |  |
| RC-RS | miR-4531 | | 3.7439 | | | 0.01 | | -14.61358307 | 1.00E-14 | | | ** |  |
| RC-RS | miR-4555 | | 7.3177 | | | 0.01 | | -16.26642126 | 4.53E-28 | | | ** |  |
| RC-RS | miR-4577 | | 700.0298 | | | 387.8301 | | -1.456489192 | 7.03E-244 | | | ** |  |
| RC-RS | miR-4578 | | 0.01 | | | 49.0704 | | 20.95969794 | 6.27E-178 | | | ** |  |
| RC-RS | miR-4621 | | 0.01 | | | 692.7453 | | 27.4890055 | 0 | | | ** |  |
| RC-RS | miR-4644 | | 3.7439 | | | 1.7356 | | -1.896031641 | 0.0025489 | | | ** |  |
| RC-RS | miR-4651 | | 0.01 | | | 84.8871 | | 22.31139534 | 2.78E-307 | | | ** |  |
| RC-RS | miR-4657 | | 1.4465 | | | 0.01 | | -12.2681753 | 3.81E-06 | | | ** |  |
| RC-RS | miR-4684-3p | | 0.01 | | | 88.2006 | | 22.40583411 | 3.00678470746066e-319 | | | ** |  |
| RC-RS | miR477a-3p | | 0.01 | | | 1.8934 | | 12.93217157 | 1.51E-07 | | | ** |  |
| RC-RS | miR479 | | 0.01 | | | 65.401 | | 21.66822141 | 6.60E-237 | | | ** |  |
| RC-RS | miR-483-5p | | 13.9546 | | | 0.01 | | -17.85845246 | 7.41E-53 | | | ** |  |
| RC-RS | miR-4856b-3p | | 0.01 | | | 34.7122 | | 20.10595051 | 4.49E-126 | | | ** |  |
| RC-RS | miR-4865-3p | | 4.8501 | | | 2.6823 | | -1.460852429 | 0.0055975 | | | ** |  |
| RC-RS | miR-4868c-3p | | 11.7423 | | | 0.01 | | -17.43274025 | 1.36E-44 | | | ** |  |
| RC-RS | miR-4882b | | 0.01 | | | 1.1045 | | 11.60287985 | 0.0001065 | | | ** |  |
| RC-RS | miR-489 | | 5.1904 | | | 13.4904 | | 2.355733057 | 1.17E-11 | | | ** |  |
| RC-RS | miR-4935 | | 0.01 | | | 1.2623 | | 11.93223668 | 2.87E-05 | | | ** |  |
| RC-RS | miR-4952-5p | | 1.4465 | | | 0.01 | | -12.2681753 | 3.81E-06 | | | ** |  |
| RC-RS | miR-4962-3p | | 6.1264 | | | 0.01 | | -15.8281868 | 1.27E-23 | | | ** |  |
| RC-RS | miR5021 | | 0.01 | | | 17.5139 | | 18.41876128 | 5.81E-64 | | | ** |  |
| RC-RS | miR5023 | | 5.4457 | | | 2.2879 | | -2.138758482 | 5.97E-05 | | | ** |  |
| RC-RS | miR5049c | | 6.7221 | | | 2.5245 | | -2.415393001 | 9.45E-07 | | | ** |  |
| RC-RS | miR5054 | | 112.6583 | | | 59.7208 | | -1.565310954 | 6.81E-46 | | | ** |  |
| RC-RS | miR5068 | | 12.6783 | | | 0.01 | | -17.62189123 | 4.33E-48 | | | ** |  |
| RC-RS | miR5070 | | 120.061 | | | 2.8401 | | -9.234236457 | 0 | | | ** |  |
| RC-RS | miR5077 | | 276.4552 | | | 125.9107 | | -1.939688131 | 7.91E-156 | | | ** |  |
| RC-RS | miR-509b | | 5.6159 | | | 0.01 | | -15.61360501 | 1.03E-21 | | | ** |  |
| RC-RS | miR-5106 | | 18.6346 | | | 34.16 | | 1.494666955 | 6.64E-14 | | | ** |  |
| RC-RS | miR-5112 | | 0.01 | | | 16.4883 | | 18.26993513 | 2.94E-60 | | | ** |  |
| RC-RS | miR-511-3p | | 1.7018 | | | 0.01 | | -12.66904745 | 4.25E-07 | | | ** |  |
| RC-RS | miR-5126 | | 15.4012 | | | 0.01 | | -18.10171923 | 2.94E-58 | | | ** |  |
| RC-RS | miR-5133 | | 0.01 | | | 78.1025 | | 22.10595208 | 8.86E-283 | | | ** |  |
| RC-RS | miR-514a-3p | | 0.01 | | | 1.1834 | | 11.77305209 | 5.53E-05 | | | ** |  |
| RC-RS | miR5169a | | 1.9571 | | | 4.2601 | | 1.918361978 | 0.0012591 | | | ** |  |
| RC-RS | miR5172-5p | | 0.01 | | | 392.5635 | | 26.08823369 | 0 | | | ** |  |
| RC-RS | miR-5189-3p | | 0.01 | | | 8.9936 | | 16.77501532 | 3.43E-33 | | | ** |  |
| RC-RS | miR-5197-3p | | 52.5852 | | | 0.01 | | -21.1303133 | 4.03E-197 | | | ** |  |
| RC-RS | miR5205b | | 8.4238 | | | 4.8913 | | -1.340689752 | 0.0006774 | | | ** |  |
| RC-RS | miR5211 | | 1.3614 | | | 0.01 | | -12.11863528 | 7.93E-06 | | | ** |  |
| RC-RS | miR5227 | | 0.01 | | | 2.8401 | | 13.93217157 | 5.74E-11 | | | ** |  |
| RC-RS | miR528-5p | | 139.4614 | | | 327.8726 | | 2.108288084 | 3.30E-208 | | | ** |  |
| RC-RS | miR5291a | | 5.7861 | | | 10.0192 | | 1.354111005 | 0.0001993 | | | ** |  |
| RC-RS | miR5293 | | 2.5527 | | | 0.01 | | -13.66904745 | 2.82E-10 | | | ** |  |
| RC-RS | miR5298b | | 11.4871 | | | 17.9872 | | 1.105978587 | 2.87E-05 | | | ** |  |
| RC-RS | miR530-3p | | 0.01 | | | 1.5778 | | 12.48245917 | 2.08E-06 | | | ** |  |
| RC-RS | miR531 | | 12.6783 | | | 21.8529 | | 1.342758603 | 4.60E-08 | | | ** |  |
| RC-RS | miR-5348-5p | | 7.1475 | | | 0.01 | | -16.20838069 | 1.96E-27 | | | ** |  |
| RC-RS | miR535 | | 81.7708 | | | 0.01 | | -22.21915171 | 4.10E-306 | | | ** |  |
| RC-RS | miR-5350b-5p | | 20.7618 | | | 9.7036 | | -1.875914334 | 1.39E-12 | | | ** |  |
| RC-RS | miR-5358b-5p | | 0.01 | | | 89.6206 | | 22.44522453 | 0 | | | ** |  |
| RC-RS | miR5380a | | 0.01 | | | 14.516 | | 17.95572919 | 3.90E-53 | | | ** |  |
| RC-RS | miR-541-3p | | 34.8866 | | | 21.6162 | | -1.180520237 | 5.64E-10 | | | ** |  |
| RC-RS | miR-5460 | | 0.01 | | | 1.1834 | | 11.77305209 | 5.53E-05 | | | ** |  |
| RC-RS | miR5483 | | 0.01 | | | 5.5224 | | 15.57219748 | 1.18E-20 | | | ** |  |
| RC-RS | miR5498 | | 3.6588 | | | 9.9403 | | 2.464976538 | 1.76E-09 | | | ** |  |
| RC-RS | miR5501 | | 118.104 | | | 234.0707 | | 1.687093666 | 9.65E-105 | | | ** |  |
| RC-RS | miR5502 | | 8.3388 | | | 0.01 | | -16.58857788 | 6.96E-32 | | | ** |  |
| RC-RS | miR5524 | | 0.01 | | | 8.3625 | | 16.59557747 | 6.53E-31 | | | ** |  |
| RC-RS | miR5526 | | 0.01 | | | 95.0641 | | 22.59065307 | 0 | | | ** |  |
| RC-RS | miR5554a-5p | | 0.01 | | | 11.3604 | | 17.35119415 | 9.73E-42 | | | ** |  |
| RC-RS | miR5565c | | 0.01 | | | 7.4158 | | 16.29926451 | 1.71E-27 | | | ** |  |
| RC-RS | miR-5606-5p | | 16.167 | | | 0.01 | | -18.22140116 | 4.06E-61 | | | ** |  |
| RC-RS | miR-5609-5p | | 0.01 | | | 3.2345 | | 14.25287756 | 2.16E-12 | | | ** |  |
| RC-RS | miR5636 | | 1597.8922 | | | 500.8025 | | -2.861476433 | 0 | | | ** |  |
| RC-RS | miR-5709-3p | | 15.4863 | | | 0.01 | | -18.11530969 | 1.41E-58 | | | ** |  |
| RC-RS | miR5720 | | 0.01 | | | 20.6695 | | 18.8273398 | 2.33E-75 | | | ** |  |
| RC-RS | miR-5728-1-5p | | 33.8655 | | | 0.01 | | -20.04504628 | 3.26E-127 | | | ** |  |
| RC-RS | miR-5735-3p | | 2.4676 | | | 0.01 | | -13.58542594 | 5.86E-10 | | | ** |  |
| RC-RS | miR5741 | | 0.01 | | | 1.1045 | | 11.60287985 | 0.0001065 | | | ** |  |
| RC-RS | miR5750 | | 4.4246 | | | 0.01 | | -15.02558407 | 2.88E-17 | | | ** |  |
| RC-RS | miR5780a | | 8.0835 | | | 0.01 | | -16.51188966 | 6.25E-31 | | | ** |  |
| RC-RS | miR-5928b | | 0.01 | | | 2.4456 | | 13.56333892 | 1.53E-09 | | | ** |  |
| RC-RS | miR-5945-3p | | 0.01 | | | 2.919 | | 13.99975279 | 2.98E-11 | | | ** |  |
| RC-RS | miR-5985 | | 0.01 | | | 11.9126 | | 17.4682524 | 9.85E-44 | | | ** |  |
| RC-RS | miR-6016-5p | | 8.5089 | | | 0.01 | | -16.63838087 | 1.61E-32 | | | ** |  |
| RC-RS | miR-602 | | 48.2456 | | | 0.01 | | -20.91789056 | 6.48E-181 | | | ** |  |
| RC-RS | miR-61 | | 0.01 | | | 3.629 | | 14.53670661 | 8.13E-14 | | | ** |  |
| RC-RS | miR6108f | | 45.2675 | | | 0.01 | | -20.7607499 | 8.58E-170 | | | ** |  |
| RC-RS | miR6150 | | 0.01 | | | 3.629 | | 14.53670661 | 8.13E-14 | | | ** |  |
| RC-RS | miR-6-1-5p | | 0.01 | | | 17.9872 | | 18.48452661 | 1.13E-65 | | | ** |  |
| RC-RS | miR6170 | | 0.01 | | | 2.2879 | | 13.39894469 | 5.67E-09 | | | ** |  |
| RC-RS | miR6173 | | 265.1383 | | | 95.853 | | -2.509305465 | 2.40E-222 | | | ** |  |
| RC-RS | miR6196 | | 16.5073 | | | 29.8998 | | 1.465105317 | 5.47E-12 | | | ** |  |
| RC-RS | miR6214 | | 12.9336 | | | 0.01 | | -17.67106137 | 4.82E-49 | | | ** |  |
| RC-RS | miR-6244 | | 49.2667 | | | 275.4887 | | 4.24524375 | 0 | | | ** |  |
| RC-RS | miR6246 | | 0.01 | | | 41.418 | | 20.54155977 | 2.72E-150 | | | ** |  |
| RC-RS | miR6249a | | 0.01 | | | 4.1812 | | 14.88603668 | 8.23E-16 | | | ** |  |
| RC-RS | miR6251 | | 1.1062 | | | 0.01 | | -11.60667296 | 7.12E-05 | | | ** |  |
| RC-RS | miR6285 | | 0.01 | | | 2.2879 | | 13.39894469 | 5.67E-09 | | | ** |  |
| RC-RS | miR-630 | | 0.01 | | | 1.1834 | | 11.77305209 | 5.53E-05 | | | ** |  |
| RC-RS | miR-6313 | | 0.01 | | | 5.4435 | | 15.53670661 | 2.27E-20 | | | ** |  |
| RC-RS | miR-6-3-5p | | 28.9304 | | | 0.01 | | -19.65659403 | 8.79E-109 | | | ** |  |
| RC-RS | miR-6385 | | 6.3817 | | | 15.3838 | | 2.1700523 | 1.02E-11 | | | ** |  |
| RC-RS | miR6421-3p | | 48.4158 | | | 0.01 | | -20.92657652 | 1.50E-181 | | | ** |  |
| RC-RS | miR6485 | | 0.01 | | | 2.7612 | | 13.86268619 | 1.11E-10 | | | ** |  |
| RC-RS | miR-6492 | | 469.1825 | | | 114.787 | | -3.472342137 | 0 | | | ** |  |
| RC-RS | miR-6504-5p | | 0.01 | | | 15.3049 | | 18.08624978 | 5.52E-56 | | | ** |  |
| RC-RS | miR-6510-5p | | 2.9781 | | | 0.01 | | -14.04918842 | 7.27E-12 | | | ** |  |
| RC-RS | miR-6527 | | 113.0837 | | | 71.3178 | | -1.136922263 | 3.37E-27 | | | ** |  |
| RC-RS | miR-6534 | | 1186.9108 | | | 1795.4102 | | 1.020752577 | 0 | | | ** |  |
| RC-RS | miR-6547-3p | | 0.8509 | | | 1.3412 | | 1.122230723 | 0.2605668 | | | ** |  |
| RC-RS | miR-65-5p | | 4.8501 | | | 0.01 | | -15.25203862 | 7.43E-19 | | | ** |  |
| RC-RS | miR-6566-3p | | 168.3067 | | | 0.01 | | -23.99949736 | 0 | | | ** |  |
| RC-RS | miR-6583-5p | | 0.01 | | | 28.4798 | | 19.61787856 | 1.45E-103 | | | ** |  |
| RC-RS | miR-6626-5p | | 1.9571 | | | 5.6013 | | 2.593404241 | 2.93E-06 | | | ** |  |
| RC-RS | miR-663 | | 46.3737 | | | 0.01 | | -20.82029343 | 6.34E-174 | | | ** |  |
| RC-RS | miR-6632-5p | | 10.2107 | | | 0.01 | | -17.08804586 | 7.11E-39 | | | ** |  |
| RC-RS | miR-6653-3p | | 5.2755 | | | 0.01 | | -15.45939104 | 1.91E-20 | | | ** |  |
| RC-RS | miR-6657-3p | | 214.2549 | | | 127.0151 | | -1.289532541 | 5.91E-62 | | | ** |  |
| RC-RS | miR-6661-5p | | 3.6588 | | | 0.01 | | -14.55687631 | 2.09E-14 | | | ** |  |
| RC-RS | miR-666-5p | | 0.01 | | | 33.7655 | | 20.03775318 | 1.18E-122 | | | ** |  |
| RC-RS | miR-668 | | 6.3817 | | | 9.6247 | | 1.013399627 | 0.0047289 | | | ** |  |
| RC-RS | miR-6692-5p | | 0.01 | | | 20.4329 | | 18.79894565 | 1.67E-74 | | | ** |  |
| RC-RS | miR-6717-5p | | 205.5758 | | | 124.0962 | | -1.244885782 | 4.48E-56 | | | ** |  |
| RC-RS | miR6725 | | 0.01 | | | 85.2816 | | 22.32283056 | 0.00E+00 | | | ** |  |
| RC-RS | miR-6777-5p | | 798.393 | | | 499.4614 | | -1.15687055 | 8.06E-187 | | | ** |  |
| RC-RS | miR-6791-5p | | 42.1192 | | | 0.01 | | -20.58296452 | 4.90E-158 | | | ** |  |
| RC-RS | miR-6800-5p | | 2.2974 | | | 0.01 | | -13.40916425 | 2.53E-09 | | | ** |  |
| RC-RS | miR-6812-5p | | 111.8924 | | | 0.01 | | -22.99262603 | 0 | | | ** |  |
| RC-RS | miR-6849-5p | | 2.4676 | | | 0.01 | | -13.58542594 | 5.86E-10 | | | ** |  |
| RC-RS | miR-6856-5p | | 2.5527 | | | 0.01 | | -13.66904745 | 2.82E-10 | | | ** |  |
| RC-RS | miR-6863 | | 0.01 | | | 2.3667 | | 13.48245917 | 2.94E-09 | | | ** |  |
| RC-RS | miR-6883-5p | | 20.6767 | | | 0.01 | | -18.82819862 | 5.85E-78 | | | ** |  |
| RC-RS | miR-6887-5p | | 85.4297 | | | 6.548 | | -6.334784684 | 2.43E-217 | | | ** |  |
| RC-RS | miR-6906-5p | | 20.5916 | | | 0.01 | | -18.81802725 | 1.22E-77 | | | ** |  |
| RC-RS | miR-6916-5p | | 0.01 | | | 24.6141 | | 19.25810502 | 1.32E-89 | | | ** |  |
| RC-RS | miR-6922-5p | | 2.0421 | | | 0.01 | | -13.11863532 | 2.27E-08 | | | ** |  |
| RC-RS | miR-6923-5p | | 9.6151 | | | 0.01 | | -16.93981754 | 1.19E-36 | | | ** |  |
| RC-RS | miR-6929-5p | | 104.5748 | | | 4.1024 | | -7.986705565 | 7.98292820577823e-313 | | | ** |  |
| RC-RS | miR-6934-5p | | 45.5228 | | | 0.01 | | -20.77462035 | 9.55E-171 | | | ** |  |
| RC-RS | miR-6949-5p | | 0.01 | | | 2.0512 | | 13.12960121 | 4.06E-08 | | | ** |  |
| RC-RS | miR-6963-5p | | 6.9773 | | | 3.3923 | | -1.778584266 | 8.75E-05 | | | ** |  |
| RC-RS | miR-6989-5p | | 95.1299 | | | 31.0832 | | -2.758747489 | 6.86E-93 | | | ** |  |
| RC-RS | miR-6996-3p | | 162.1802 | | | 0.01 | | -23.9080458 | 0 | | | ** |  |
| RC-RS | miR-7002-5p | | 551.2937 | | | 127.6463 | | -3.608213155 | 0 | | | ** |  |
| RC-RS | miR-7011-5p | | 43.5657 | | | 0.01 | | -20.66624243 | 1.94E-163 | | | ** |  |
| RC-RS | miR-7014-5p | | 23.3996 | | | 14.1216 | | -1.245516267 | 1.03E-07 | | | ** |  |
| RC-RS | miR-7022-5p | | 0.01 | | | 62.4031 | | 21.55249604 | 4.43E-226 | | | ** |  |
| RC-RS | miR-7027-5p | | 2.5527 | | | 0.01 | | -13.66904745 | 2.82E-10 | | | ** |  |
| RC-RS | miR-7033-5p | | 8.1686 | | | 3.7868 | | -1.896035402 | 7.68E-06 | | | ** |  |
| RC-RS | miR-7056-5p | | 9.53 | | | 0.01 | | -16.91789208 | 2.48E-36 | | | ** |  |
| RC-RS | miR-7094b-2-5p | | 0.01 | | | 1.4989 | | 12.3559379 | 4.01E-06 | | | ** |  |
| RC-RS | miR-7111-5p | | 0.01 | | | 5.4435 | | 15.53670661 | 2.27E-20 | | | ** |  |
| RC-RS | miR-7170-5p | | 158.5214 | | | 70.6078 | | -1.994620405 | 3.13E-94 | | | ** |  |
| RC-RS | miR-72 | | 0.01 | | | 92.6185 | | 22.52637505 | 0 | | | ** |  |
| RC-RS | miR-7225-5p | | 0.01 | | | 66.8999 | | 21.72410766 | 2.55E-242 | | | ** |  |
| RC-RS | miR-7247-5p | | 88.2376 | | | 50.0171 | | -1.400042121 | 2.53E-30 | | | ** |  |
| RC-RS | miR-7267-5p | | 156.3091 | | | 94.1174 | | -1.251137294 | 2.01E-43 | | | ** |  |
| RC-RS | miR-7289-5p | | 48.586 | | | 105.5567 | | 1.913636496 | 7.85E-59 | | | ** |  |
| RC-RS | miR-7355-5p | | 1.872 | | | 0.01 | | -12.90413767 | 9.83E-08 | | | ** |  |
| RC-RS | miR-7380-3p | | 0.01 | | | 2.3667 | | 13.48245917 | 2.94E-09 | | | ** |  |
| RC-RS | miR-7384-3p | | 51.9896 | | | 0.01 | | -21.10221943 | 6.76E-195 | | | ** |  |
| RC-RS | miR-7385e-3p | | 545.848 | | | 0.01 | | -26.90123398 | 0 | | | ** |  |
| RC-RS | miR-7388c-5p | | 0.01 | | | 6.7847 | | 16.07990461 | 3.26E-25 | | | ** |  |
| RC-RS | miR-7398f-5p | | 208.8943 | | | 713.1781 | | 3.028380927 | 0 | | | ** |  |
| RC-RS | miR-7439-5p | | 7.5729 | | | 0.01 | | -16.35096617 | 5.04E-29 | | | ** |  |
| RC-RS | miR-7440-3p | | 0.01 | | | 137.7444 | | 23.50527775 | 0 | | | ** |  |
| RC-RS | miR-7446-3p | | 0.01 | | | 57.0385 | | 21.33080379 | 1.05E-206 | | | ** |  |
| RC-RS | miR-7448-3p | | 127.7191 | | | 194.8617 | | 1.041906565 | 4.09E-39 | | | ** |  |
| RC-RS | miR-7449-3p | | 0.01 | | | 3.2345 | | 14.25287756 | 2.16E-12 | | | ** |  |
| RC-RS | miR-7455-5p | | 7.1475 | | | 0.6311 | | -5.985850692 | 1.14E-18 | | | ** |  |
| RC-RS | miR-745-5p | | 7.1475 | | | 4.339 | | -1.230978275 | 0.00367 | | | ** |  |
| RC-RS | miR7485 | | 0.936 | | | 2.9979 | | 2.870905106 | 0.0002579 | | | ** |  |
| RC-RS | miR7497 | | 1.3614 | | | 19.644 | | 6.583201154 | 1.98E-51 | | | ** |  |
| RC-RS | miR7504n | | 2.1272 | | | 0.01 | | -13.21932922 | 1.09E-08 | | | ** |  |
| RC-RS | miR-761 | | 1.0211 | | | 0.01 | | -11.40924477 | 0.0001479 | | | ** |  |
| RC-RS | miR-7648-3p | | 1.0211 | | | 0.01 | | -11.40924477 | 0.0001479 | | | ** |  |
| RC-RS | miR-7665-5p | | 2.6378 | | | 0.01 | | -13.74992647 | 1.36E-10 | | | ** |  |
| RC-RS | miR-766-5p | | 1.1913 | | | 0.01 | | -11.78946163 | 3.42E-05 | | | ** |  |
| RC-RS | miR-7667-3p | | 0.01 | | | 2.2879 | | 13.39894469 | 5.67E-09 | | | ** |  |
| RC-RS | miR-7847-3p | | 20.0811 | | | 0.01 | | -18.75611263 | 9.80E-76 | | | ** |  |
| RC-RS | miR-790 | | 1.0211 | | | 0.01 | | -11.40924477 | 0.0001479 | | | ** |  |
| RC-RS | miR-7907b-3p | | 0.01 | | | 30.2154 | | 19.76377685 | 7.81E-110 | | | ** |  |
| RC-RS | miR-7920-3p | | 0.01 | | | 1.42 | | 12.22257343 | 7.72E-06 | | | ** |  |
| RC-RS | miR-7977 | | 150.2677 | | | 93.9596 | | -1.158061344 | 1.02E-36 | | | ** |  |
| RC-RS | miR8000 | | 0.01 | | | 19.0128 | | 18.62128818 | 2.24E-69 | | | ** |  |
| RC-RS | miR8003 | | 0.01 | | | 2.7612 | | 13.86268619 | 1.11E-10 | | | ** |  |
| RC-RS | miR8037 | | 6.7221 | | | 0.01 | | -16.05704321 | 7.59E-26 | | | ** |  |
| RC-RS | miR-8085 | | 56.6695 | | | 0.01 | | -21.31479617 | 2.25E-212 | | | ** |  |
| RC-RS | miR-8100 | | 4.5097 | | | 0.01 | | -15.07256899 | 1.39E-17 | | | ** |  |
| RC-RS | miR-8117 | | 2482.5658 | | | 4739.8734 | | 1.595002361 | 0 | | | ** |  |
| RC-RS | miR8134 | | 107.3827 | | | 0.01 | | -22.89116564 | 0 | | | ** |  |
| RC-RS | miR815a | | 96.4913 | | | 0.01 | | -22.62740422 | 0 | | | ** |  |
| RC-RS | miR-8189-3p | | 2.0421 | | | 0.01 | | -13.11863532 | 2.27E-08 | | | ** |  |
| RC-RS | miR-8209-5p | | 24.6759 | | | 6.3902 | | -3.332127628 | 1.75E-32 | | | ** |  |
| RC-RS | miR-8268-5p | | 357.3751 | | | 0.01 | | -25.85661761 | 0 | | | ** |  |
| RC-RS | miR-8269-5p | | 2.7229 | | | 0.01 | | -13.82823716 | 6.53E-11 | | | ** |  |
| RC-RS | miR827 | | 11.8274 | | | 23.1152 | | 1.652599746 | 2.04E-11 | | | ** |  |
| RC-RS | miR-8293-3p | | 0.01 | | | 1.3412 | | 12.0817669 | 1.49E-05 | | | ** |  |
| RC-RS | miR-8301-3p | | 0.01 | | | 73.6057 | | 21.95970129 | 1.54E-266 | | | ** |  |
| RC-RS | miR-8364e-3p | | 42.5447 | | | 66.5843 | | 1.104690675 | 8.04E-16 | | | ** |  |
| RC-RS | miR-8379b-5p | | 0.01 | | | 1.0256 | | 11.42008992 | 0.0002052 | | | ** |  |
| RC-RS | miR845a-3p | | 7.5729 | | | 0.01 | | -16.35096617 | 5.04E-29 | | | ** |  |
| RC-RS | miR-8472-5p | | 0.01 | | | 1.9723 | | 13.03286141 | 7.82E-08 | | | ** |  |
| RC-RS | miR847-5p | | 0.01 | | | 53.4095 | | 21.16867431 | 1.34E-193 | | | ** |  |
| RC-RS | miR-84a | | 2076.1793 | | | 3890.6076 | | 1.548927391 | 0 | | | ** |  |
| RC-RS | miR-8501 | | 253.5662 | | | 439.5039 | | 1.356520322 | 1.74E-135 | | | ** |  |
| RC-RS | miR-8528a | | 159.0319 | | | 94.9063 | | -1.273142157 | 1.84E-45 | | | ** |  |
| RC-RS | miR854 | | 12.0827 | | | 0.01 | | -17.50321965 | 7.26E-46 | | | ** |  |
| RC-RS | miR8564 | | 0.01 | | | 2.0512 | | 13.12960121 | 4.06E-08 | | | ** |  |
| RC-RS | miR8577 | | 22.4636 | | | 0.01 | | -19.03262773 | 1.24E-84 | | | ** |  |
| RC-RS | miR8598 | | 0.01 | | | 81.9682 | | 22.22509748 | 9.70E-297 | | | ** |  |
| RC-RS | miR8660 | | 0.01 | | | 1.8145 | | 12.82719532 | 2.90E-07 | | | ** |  |
| RC-RS | miR8708 | | 4.3396 | | | 2.0512 | | -1.848142229 | 0.0014469 | | | ** |  |
| RC-RS | miR8714 | | 0.01 | | | 71.6334 | | 21.89271395 | 2.05E-259 | | | ** |  |
| RC-RS | miR8752 | | 0.01 | | | 15.8572 | | 18.17368176 | 5.59E-58 | | | ** |  |
| RC-RS | miR-875-3p | | 13.4441 | | | 0.01 | | -17.76653626 | 5.98E-51 | | | ** |  |
| RC-RS | miR8772 | | 1.7018 | | | 4.1024 | | 2.170065036 | 0.0004884 | | | ** |  |
| RC-RS | miR8775 | | 413.7042 | | | 38.4201 | | -5.861343976 | 0 | | | ** |  |
| RC-RS | miR8786a | | 5.6159 | | | 0.01 | | -15.61360501 | 1.03E-21 | | | ** |  |
| RC-RS | miR-8908n | | 0.01 | | | 3.7868 | | 14.64168286 | 2.19E-14 | | | ** |  |
| RC-RS | miR-8964 | | 14.8055 | | | 5.7591 | | -2.328726933 | 1.30E-12 | | | ** |  |
| RC-RS | miR-8979 | | 29.0155 | | | 0.01 | | -19.66383824 | 4.23E-109 | | | ** |  |
| RC-RS | miR904a | | 4.8501 | | | 0.01 | | -15.25203862 | 7.43E-19 | | | ** |  |
| RC-RS | miR-9168 | | 29.7813 | | | 62.2453 | | 1.818164718 | 1.02E-32 | | | ** |  |
| RC-RS | miR-9191 | | 44.5017 | | | 72.5012 | | 1.203742837 | 1.24E-19 | | | ** |  |
| RC-RS | miR-9256b | | 1348.07 | | | 0.01 | | -29.13099263 | 0 | | | ** |  |
| RC-RS | miR-927a-5p | | 0.01 | | | 8.9936 | | 16.77501532 | 3.43E-33 | | | ** |  |
| RC-RS | miR-92a-3p | | 1.3614 | | | 0.01 | | -12.11863528 | 7.93E-06 | | | ** |  |
| RC-RS | miR-9308 | | 8.7642 | | | 0.01 | | -16.71129094 | 1.79E-33 | | | ** |  |
| RC-RS | miR-932-3p | | 10.8914 | | | 29.3476 | | 2.444691939 | 6.47E-25 | | | ** |  |
| RC-RS | miR-9383-5p | | 3253.475 | | | 0.01 | | -31.30395389 | 0 | | | ** |  |
| RC-RS | miR-9384-3p | | 15.0608 | | | 0.01 | | -18.04659714 | 5.49E-57 | | | ** |  |
| RC-RS | miR-9427 | | 0.01 | | | 2.2879 | | 13.39894469 | 5.67E-09 | | | ** |  |
| RC-RS | miR-9445 | | 0.01 | | | 7.8891 | | 16.45185272 | 3.34E-29 | | | ** |  |
| RC-RS | miR-9458 | | 1.1062 | | | 0.01 | | -11.60667296 | 7.12E-05 | | | ** |  |
| RC-RS | miR946a-5p | | 24.3355 | | | 0.01 | | -19.23003053 | 1.27E-91 | | | ** |  |
| RC-RS | miR-967-5p | | 468.8421 | | | 842.2444 | | 1.444770919 | 6.48E-287 | | | ** |  |
| RC-RS | miR9760 | | 865.7838 | | | 0.01 | | -28.03893005 | 0 | | | ** |  |
| RC-RS | miR9774 | | 0.01 | | | 1.5778 | | 12.48245917 | 2.08E-06 | | | ** |  |
| RC-RS | miR9778 | | 16.8477 | | | 0.01 | | -18.32311643 | 1.16E-63 | | | ** |  |
| RC-RS | miR-9875 | | 54.6273 | | | 21.5373 | | -2.295505641 | 1.65E-41 | | | ** |  |
| RC-RS | miR-9877 | | 3.9141 | | | 0.01 | | -14.72322896 | 2.32E-15 | | | ** |  |
| RC-RS | miR-989 | | 3.9992 | | | 0.01 | | -14.77627644 | 1.12E-15 | | | ** |  |
| RC-RS | miR-B10 | | 0.01 | | | 458.9901 | | 26.47379174 | 0 | | | ** |  |
| RC-RS | miR-H16 | | 0.01 | | | 7.6525 | | 16.37675461 | 2.39E-28 | | | ** |  |
| RC-RS | miR-H25 | | 14.8906 | | | 6.548 | | -2.026242124 | 1.76E-10 | | | ** |  |
| RC-RS | miR-H3 | | 0.01 | | | 12.4648 | | 17.58000557 | 9.98E-46 | | | ** |  |
| RC-RS | miR-LLT9 | | 0.01 | | | 2.4456 | | 13.56333892 | 1.53E-09 | | | ** |  |
| RC-RS | miR-rL1-11-3p | | 0.01 | | | 13.0171 | | 17.68693266 | 1.01E-47 | | | ** |  |
| RC-RS | miR-rL1-15-3p | | 0.01 | | | 1.9723 | | 13.03286141 | 7.82E-08 | | | ** |  |
| RC-RS | miR-rL1-32-5p | | 1.0211 | | | 0.01 | | -11.40924477 | 0.0001479 | | | ** |  |
| RC-RS | miR-US25-2-3p | | 0.01 | | | 1.1045 | | 11.60287985 | 0.0001065 | | | ** |  |
| RC-RS | miR-1a-3p | | 3.5738 | | | 2.209 | | -1.186512775 | 0.0472755 | | | * |  |
| RC-RS | miR-221 | | 4.5097 | | | 2.919 | | -1.072816205 | 0.041251 | | | * |  |
| RC-RS | miR-2716 | | 4.5097 | | | 2.6034 | | -1.355017576 | 0.012276 | | | * |  |
| RC-RS | miR-3077-5p | | 4.2545 | | | 2.5245 | | -1.28724819 | 0.02002 | | | * |  |
| RC-RS | miR-3825-3p | | 2.7229 | | | 1.4989 | | -1.472299265 | 0.0373321 | | | * |  |
| RC-RS | miR408-3p | | 1.2763 | | | 2.7612 | | 1.903246633 | 0.0102796 | | | * |  |
| RC-RS | miR-458b-3p | | 3.1483 | | | 5.2068 | | 1.240804272 | 0.0132619 | | | * |  |
| RC-RS | miR5217 | | 4.765 | | | 7.1791 | | 1.010879807 | 0.0150669 | | | * |  |
| RC-RS | miR-54-5p | | 1.9571 | | | 3.7079 | | 1.575972577 | 0.0105638 | | | * |  |
| RC-RS | miR-6607-5p | | 1.7018 | | | 0.7889 | | -1.896099594 | 0.0436304 | | | * |  |
| RC-RS | miR-9857-5p | | 1.4465 | | | 0.4733 | | -2.755286874 | 0.0137687 | | | * |  |
| RC-RS | let-7b | | 30.462 | | | 42.4436 | | 0.818063311 | 9.50E-07 | | |  |  |
| RC-RS | let-7b-5p | | 31.0576 | | | 42.6802 | | 0.784017077 | 2.28E-06 | | |  |  |
| RC-RS | let-7c | | 68.7522 | | | 92.3029 | | 0.726491184 | 8.96E-11 | | |  |  |
| RC-RS | let-7d | | 5.0203 | | | 6.7058 | | 0.713953253 | 0.0874181 | | |  |  |
| RC-RS | let-7d-5p | | 6.5519 | | | 7.8891 | | 0.458059089 | 0.2219845 | | |  |  |
| RC-RS | let-7e-5p | | 1.7869 | | | 2.9979 | | 1.276138828 | 0.0555203 | | |  |  |
| RC-RS | let-7i-5p | | 2.5527 | | | 3.4712 | | 0.758015239 | 0.1963237 | | |  |  |
| RC-RS | miR-101 | | 5.3606 | | | 5.2068 | | -0.0717952 | 0.8658567 | | |  |  |
| RC-RS | miR-101-3p | | 2.7229 | | | 3.3923 | | 0.54211981 | 0.3510228 | | |  |  |
| RC-RS | miR-103a-3p | | 17.6135 | | | 19.0128 | | 0.188540958 | 0.4214128 | | |  |  |
| RC-RS | miR1048-5p | | 3.7439 | | | 4.6546 | | 0.536983635 | 0.2772285 | | |  |  |
| RC-RS | miR-107 | | 18.9749 | | | 21.6162 | | 0.321423424 | 0.1487942 | | |  |  |
| RC-RS | miR-10b-3p | | 4.8501 | | | 6.4691 | | 0.710388306 | 0.0948091 | | |  |  |
| RC-RS | miR1111 | | 7.9133 | | | 9.7825 | | 0.522980105 | 0.1224357 | | |  |  |
| RC-RS | miR1120 | | 1.6167 | | | 2.0512 | | 0.58707382 | 0.4383747 | | |  |  |
| RC-RS | miR1122c-3p | | 12.0827 | | | 16.2516 | | 0.731053511 | 0.0063281 | | |  |  |
| RC-RS | miR1130b-3p | | 10.2107 | | | 12.1493 | | 0.428730764 | 0.1539433 | | |  |  |
| RC-RS | miR1140 | | 270.4989 | | | 371.894 | | 0.785125114 | 1.93E-44 | | |  |  |
| RC-RS | miR1171 | | 24.8461 | | | 18.6184 | | -0.711653003 | 0.0009457 | | |  |  |
| RC-RS | miR-1181 | | 3.5738 | | | 4.1812 | | 0.387132764 | 0.4520302 | | |  |  |
| RC-RS | miR-1193-5p | | 34.8015 | | | 48.0449 | | 0.795322451 | 3.72E-07 | | |  |  |
| RC-RS | miR-124a | | 4.4246 | | | 3.4712 | | -0.59852134 | 0.2359133 | | |  |  |
| RC-RS | miR-1261 | | 344.1012 | | | 267.4418 | | -0.621591896 | 2.02E-27 | | |  |  |
| RC-RS | miR-1273e | | 1009.84 | | | 853.3681 | | -0.415217454 | 8.25E-37 | | |  |  |
| RC-RS | miR-1290 | | 1037.1537 | | | 930.3661 | | -0.267981742 | 4.00E-17 | | |  |  |
| RC-RS | miR-1329-5p | | 103.809 | | | 120.6249 | | 0.370273684 | 8.92E-05 | | |  |  |
| RC-RS | miR-1357 | | 31.1427 | | | 43.4692 | | 0.822445062 | 6.19E-07 | | |  |  |
| RC-RS | miR-1397-5p | | 28.9304 | | | 33.4499 | | 0.357998384 | 0.0460369 | | |  |  |
| RC-RS | miR-140 | | 29.2707 | | | 32.1088 | | 0.228239417 | 0.2068959 | | |  |  |
| RC-RS | miR-140-3p | | 20.7618 | | | 22.4052 | | 0.187878847 | 0.3841573 | | |  |  |
| RC-RS | miR-142-5p | | 6.8071 | | | 8.2836 | | 0.484163668 | 0.186943 | | |  |  |
| RC-RS | miR-14-5p | | 11.7423 | | | 11.597 | | -0.030708584 | 0.9141524 | | |  |  |
| RC-RS | miR1510a-5p | | 15.4863 | | | 21.6951 | | 0.831466529 | 0.0003799 | | |  |  |
| RC-RS | miR1516c | | 1.872 | | | 2.4456 | | 0.659201264 | 0.343482 | | |  |  |
| RC-RS | miR156a-5p | | 20477.0839 | | | 21000.0193 | | 0.06219267 | 3.06E-19 | | |  |  |
| RC-RS | miR156b-3p | | 15.8266 | | | 12.7015 | | -0.542517631 | 0.0407467 | | |  |  |
| RC-RS | miR159a.1 | | 56.0739 | | | 62.2453 | | 0.257513071 | 0.0478049 | | |  |  |
| RC-RS | miR160a-3p | | 35.9928 | | | 46.467 | | 0.629951782 | 5.66E-05 | | |  |  |
| RC-RS | miR-1627-5p | | 27.5689 | | | 32.5821 | | 0.412056995 | 0.0242031 | | |  |  |
| RC-RS | miR164a-3p | | 1.9571 | | | 2.7612 | | 0.848905561 | 0.202315 | | |  |  |
| RC-RS | miR164a-5p | | 569.4177 | | | 691.0885 | | 0.477608615 | 5.13E-33 | | |  |  |
| RC-RS | miR-1654 | | 182.2613 | | | 183.738 | | 0.019901737 | 0.7880999 | | |  |  |
| RC-RS | miR165a-3p | | 21.953 | | | 23.5885 | | 0.177217462 | 0.3990909 | | |  |  |
| RC-RS | miR-16-5p | | 1.872 | | | 2.2879 | | 0.494807034 | 0.4857064 | | |  |  |
| RC-RS | miR166a-3p | | 8207.2898 | | | 10132.0208 | | 0.519595939 | 0 | | |  |  |
| RC-RS | miR-1670 | | 565.7589 | | | 576.3805 | | 0.045873282 | 0.2726041 | | |  |  |
| RC-RS | miR167a-5p | | 2016.5317 | | | 2968.5251 | | 0.953685457 | 0 | | |  |  |
| RC-RS | miR167e-3p | | 158.7767 | | | 221.6848 | | 0.823147876 | 1.74E-29 | | |  |  |
| RC-RS | miR168a-5p | | 47414.9143 | | | 57006.916 | | 0.454381314 | 0 | | |  |  |
| RC-RS | miR-1692 | | 162.946 | | | 130.4864 | | -0.547888949 | 3.38E-11 | | |  |  |
| RC-RS | miR169b-5p | | 38.6305 | | | 40.4713 | | 0.114808795 | 0.4711155 | | |  |  |
| RC-RS | miR169e-3p | | 19.3153 | | | 14.9894 | | -0.625341709 | 0.0097731 | | |  |  |
| RC-RS | miR172a | | 45.9482 | | | 66.111 | | 0.897291689 | 2.77E-11 | | |  |  |
| RC-RS | miR-1839 | | 2.3825 | | | 1.9723 | | -0.466007991 | 0.4921961 | | |  |  |
| RC-RS | miR-185-5p | | 12.423 | | | 10.7292 | | -0.361512233 | 0.2183389 | | |  |  |
| RC-RS | miR1858a | | 63.7319 | | | 64.4543 | | 0.027798261 | 0.8247372 | | |  |  |
| RC-RS | miR-188 | | 445.2724 | | | 377.732 | | -0.405710075 | 1.86E-16 | | |  |  |
| RC-RS | miR1881 | | 50.5431 | | | 42.7591 | | -0.412475449 | 0.0048403 | | |  |  |
| RC-RS | miR-190-5p | | 1.3614 | | | 1.4989 | | 0.237302596 | 0.7865201 | | |  |  |
| RC-RS | miR-191-5p | | 10.5511 | | | 9.0725 | | -0.372368074 | 0.243139 | | |  |  |
| RC-RS | miR-192-5p | | 0.936 | | | 1.1045 | | 0.408253486 | 0.6945906 | | |  |  |
| RC-RS | miR-1b | | 6.5519 | | | 7.3369 | | 0.279090164 | 0.465883 | | |  |  |
| RC-RS | miR-2022-5p | | 5.8712 | | | 8.5203 | | 0.918432941 | 0.015061 | | |  |  |
| RC-RS | miR-208a-3p | | 319.17 | | | 321.6402 | | 0.019014347 | 0.73372 | | |  |  |
| RC-RS | miR-2127 | | 66.9653 | | | 65.2432 | | -0.064253982 | 0.6001567 | | |  |  |
| RC-RS | miR-215 | | 0.6807 | | | 1.1045 | | 1.193755838 | 0.284025 | | |  |  |
| RC-RS | miR-219-3p | | 1798.1074 | | | 2333.5283 | | 0.642833976 | 2.91E-186 | | |  |  |
| RC-RS | miR-2211-3p | | 33.0997 | | | 39.919 | | 0.462008145 | 0.0053709 | | |  |  |
| RC-RS | miR-221-3p | | 3.0632 | | | 2.4456 | | -0.555336641 | 0.3582397 | | |  |  |
| RC-RS | miR-223 | | 2.9781 | | | 1.9723 | | -1.016327011 | 0.1146869 | | |  |  |
| RC-RS | miR-2230-5p | | 39.5665 | | | 41.1024 | | 0.093925987 | 0.5517154 | | |  |  |
| RC-RS | miR-2392 | | 7.9133 | | | 11.4393 | | 0.908857268 | 0.0052141 | | |  |  |
| RC-RS | miR-23a | | 2.0421 | | | 1.9723 | | -0.085773874 | 0.898553 | | |  |  |
| RC-RS | miR-23a-3p | | 2.0421 | | | 2.5245 | | 0.523014996 | 0.4391317 | | |  |  |
| RC-RS | miR-24 | | 1.6167 | | | 2.1301 | | 0.680161839 | 0.3639904 | | |  |  |
| RC-RS | miR-2410 | | 28.2497 | | | 20.354 | | -0.80846755 | 7.30E-05 | | |  |  |
| RC-RS | miR-24-3p | | 1.7869 | | | 2.3667 | | 0.693066546 | 0.3290562 | | |  |  |
| RC-RS | miR-2478 | | 11.6572 | | | 8.3625 | | -0.819223607 | 0.0099595 | | |  |  |
| RC-RS | miR-25-3p | | 3.829 | | | 3.3134 | | -0.356698557 | 0.4995308 | | |  |  |
| RC-RS | miR-254-3p | | 490.9653 | | | 505.7727 | | 0.073283501 | 0.1015662 | | |  |  |
| RC-RS | miR-2544-3p | | 1.872 | | | 1.3412 | | -0.822370749 | 0.3028653 | | |  |  |
| RC-RS | miR-2561-3p | | 116.5724 | | | 98.9298 | | -0.404725414 | 2.65E-05 | | |  |  |
| RC-RS | miR-2-5p | | 1676.1744 | | | 1763.6169 | | 0.125418193 | 1.93E-07 | | |  |  |
| RC-RS | miR-26b | | 9.6151 | | | 9.6247 | | 0.002461201 | 0.9965005 | | |  |  |
| RC-RS | miR-278-5p | | 2.0421 | | | 1.6567 | | -0.515829886 | 0.4842665 | | |  |  |
| RC-RS | miR-2828 | | 5.701 | | | 5.838 | | 0.058566506 | 0.8918182 | | |  |  |
| RC-RS | miR-2840 | | 3.6588 | | | 4.8913 | | 0.71602436 | 0.1442821 | | |  |  |
| RC-RS | miR2916 | | 346.3986 | | | 424.0412 | | 0.498786417 | 1.52E-22 | | |  |  |
| RC-RS | miR-296 | | 20.8469 | | | 24.8508 | | 0.433291757 | 0.0389812 | | |  |  |
| RC-RS | miR-2962-5p | | 2.2123 | | | 1.6567 | | -0.713267362 | 0.3250054 | | |  |  |
| RC-RS | miR-2988 | | 26.633 | | | 23.5096 | | -0.307652114 | 0.1230573 | | |  |  |
| RC-RS | miR-29a | | 5.1054 | | | 4.5757 | | -0.270156685 | 0.550805 | | |  |  |
| RC-RS | miR-29a-3p | | 5.701 | | | 5.3646 | | -0.150000043 | 0.7218095 | | |  |  |
| RC-RS | miR-30d | | 1.6167 | | | 1.0256 | | -1.122437471 | 0.2065946 | | |  |  |
| RC-RS | miR-3173 | | 263.9471 | | | 311.4632 | | 0.408253349 | 4.61E-12 | | |  |  |
| RC-RS | miR-317-3p | | 11.4871 | | | 13.4904 | | 0.396467294 | 0.1631936 | | |  |  |
| RC-RS | miR319b | | 45.5228 | | | 57.6696 | | 0.583322383 | 2.99E-05 | | |  |  |
| RC-RS | miR319b-3p | | 16.6775 | | | 17.5139 | | 0.12068695 | 0.6196688 | | |  |  |
| RC-RS | miR-320a | | 11.6572 | | | 12.7804 | | 0.226872133 | 0.4301571 | | |  |  |
| RC-RS | miR-330-3p | | 4.765 | | | 3.7868 | | -0.566697847 | 0.2425091 | | |  |  |
| RC-RS | miR-342 | | 1.6167 | | | 1.6567 | | 0.060278035 | 0.9455845 | | |  |  |
| RC-RS | miR3440b-3p | | 26.8031 | | | 32.8188 | | 0.499386917 | 0.0065806 | | |  |  |
| RC-RS | miR-344d-1-5p | | 2.3825 | | | 3.3923 | | 0.871487573 | 0.1466167 | | |  |  |
| RC-RS | miR-3471 | | 1.1913 | | | 0.7889 | | -1.016513741 | 0.3224031 | | |  |  |
| RC-RS | miR-3473b | | 14.7205 | | | 11.7548 | | -0.554867499 | 0.043845 | | |  |  |
| RC-RS | miR-3488 | | 36.5033 | | | 30.9254 | | -0.408974318 | 0.0175514 | | |  |  |
| RC-RS | miR3522a | | 101.2563 | | | 124.964 | | 0.518838164 | 3.71E-08 | | |  |  |
| RC-RS | miR-3569 | | 38.7156 | | | 38.1045 | | -0.039239473 | 0.8064534 | | |  |  |
| RC-RS | miR-35h | | 134.2709 | | | 154.2327 | | 0.341837297 | 4.08E-05 | | |  |  |
| RC-RS | miR-3742 | | 8.6791 | | | 9.7036 | | 0.275187948 | 0.4071482 | | |  |  |
| RC-RS | miR-3772 | | 57.18 | | | 45.1259 | | -0.583891975 | 3.05E-05 | | |  |  |
| RC-RS | miR390a-5p | | 65.689 | | | 50.3327 | | -0.656718695 | 6.05E-07 | | |  |  |
| RC-RS | miR-3934 | | 410.7261 | | | 340.8108 | | -0.460209962 | 4.73E-19 | | |  |  |
| RC-RS | miR393a | | 5.1054 | | | 7.1791 | | 0.840701753 | 0.0396975 | | |  |  |
| RC-RS | miR394a | | 1.7869 | | | 1.2623 | | -0.857155936 | 0.2961112 | | |  |  |
| RC-RS | miR394b-3p | | 72.6663 | | | 54.9873 | | -0.687544927 | 4.32E-08 | | |  |  |
| RC-RS | miR-3961 | | 33.2699 | | | 47.0982 | | 0.857241942 | 7.06E-08 | | |  |  |
| RC-RS | miR396e-5p | | 58.2862 | | | 53.2517 | | -0.222795068 | 0.0956708 | | |  |  |
| RC-RS | miR399b | | 0.8509 | | | 1.42 | | 1.263037253 | 0.1981713 | | |  |  |
| RC-RS | miR-4006b-5p | | 2349.7414 | | | 1799.7492 | | -0.657659439 | 2.92E-196 | | |  |  |
| RC-RS | miR-4089-5p | | 10.2107 | | | 7.968 | | -0.61164984 | 0.0658716 | | |  |  |
| RC-RS | miR408b-5p | | 50.7983 | | | 73.369 | | 0.906708378 | 1.43E-12 | | |  |  |
| RC-RS | miR-4128-3p | | 198.173 | | | 139.2433 | | -0.870401965 | 2.47E-29 | | |  |  |
| RC-RS | miR-4175-3p | | 8121.9452 | | | 8134.3331 | | 0.003758839 | 0.734459 | | |  |  |
| RC-RS | miR-4186-5p | | 34.8866 | | | 29.2687 | | -0.433043707 | 0.0141908 | | |  |  |
| RC-RS | miR-423-5p | | 8.8493 | | | 8.3625 | | -0.139545748 | 0.6801755 | | |  |  |
| RC-RS | miR-4271 | | 1689.0229 | | | 1708.3141 | | 0.028009266 | 0.2478585 | | |  |  |
| RC-RS | miR4342 | | 2.0421 | | | 3.3134 | | 1.193681405 | 0.0572638 | | |  |  |
| RC-RS | miR4386 | | 13.1038 | | | 14.1216 | | 0.184487569 | 0.4982807 | | |  |  |
| RC-RS | miR4394 | | 11.0616 | | | 8.9936 | | -0.510442036 | 0.1064071 | | |  |  |
| RC-RS | miR4402 | | 5.3606 | | | 6.2324 | | 0.371636369 | 0.3754583 | | |  |  |
| RC-RS | miR-4430 | | 74.283 | | | 53.9617 | | -0.788249297 | 3.27E-10 | | |  |  |
| RC-RS | miR-4436a | | 121.5075 | | | 93.2496 | | -0.652821197 | 1.49E-11 | | |  |  |
| RC-RS | miR-4444 | | 91.3859 | | | 73.7634 | | -0.528352552 | 1.61E-06 | | |  |  |
| RC-RS | miR-4448 | | 26.1224 | | | 20.5118 | | -0.596334551 | 0.0040511 | | |  |  |
| RC-RS | miR444f | | 144.1413 | | | 202.8298 | | 0.842423009 | 3.21E-28 | | |  |  |
| RC-RS | miR-4451 | | 118.3592 | | | 129.4608 | | 0.221114122 | 0.0138493 | | |  |  |
| RC-RS | miR-4492 | | 329.2956 | | | 309.6487 | | -0.15172058 | 0.0066164 | | |  |  |
| RC-RS | miR-451a | | 8.2537 | | | 7.968 | | -0.086883091 | 0.8021582 | | |  |  |
| RC-RS | miR-4547-5p | | 9.1896 | | | 12.5437 | | 0.767380797 | 0.0121911 | | |  |  |
| RC-RS | miR-4630 | | 17.6135 | | | 18.3817 | | 0.105286373 | 0.6569469 | | |  |  |
| RC-RS | miR-4637 | | 4.5097 | | | 3.8657 | | -0.380027403 | 0.436262 | | |  |  |
| RC-RS | miR-4646-5p | | 1.6167 | | | 1.6567 | | 0.060278035 | 0.9455845 | | |  |  |
| RC-RS | miR-466i-3p | | 3.1483 | | | 2.919 | | -0.186505648 | 0.7425039 | | |  |  |
| RC-RS | miR-4690-5p | | 9.53 | | | 8.2836 | | -0.345694501 | 0.3016656 | | |  |  |
| RC-RS | miR-4699-5p | | 5.8712 | | | 5.2068 | | -0.296187208 | 0.4846383 | | |  |  |
| RC-RS | miR-4717-3p | | 7.8282 | | | 6.8636 | | -0.324320174 | 0.3785766 | | |  |  |
| RC-RS | miR-4764-5p | | 2.2123 | | | 2.8401 | | 0.616098792 | 0.3368972 | | |  |  |
| RC-RS | miR-4825-5p | | 18.7196 | | | 20.1173 | | 0.177596136 | 0.4354656 | | |  |  |
| RC-RS | miR482e-5p | | 206.1714 | | | 162.4374 | | -0.588004153 | 1.58E-15 | | |  |  |
| RC-RS | miR-4835-5p | | 14.5503 | | | 17.6717 | | 0.47933317 | 0.0554487 | | |  |  |
| RC-RS | miR-486-5p | | 2.1272 | | | 1.2623 | | -1.287092521 | 0.1016355 | | |  |  |
| RC-RS | miR-4887 | | 10.5511 | | | 12.4648 | | 0.411079889 | 0.1653461 | | |  |  |
| RC-RS | miR-4898 | | 5.3606 | | | 5.838 | | 0.210406171 | 0.6229466 | | |  |  |
| RC-RS | miR5059 | | 89.4289 | | | 74.8679 | | -0.438308165 | 7.12E-05 | | |  |  |
| RC-RS | miR5066 | | 12.338 | | | 8.2836 | | -0.98259078 | 0.0017602 | | |  |  |
| RC-RS | miR5072 | | 72.4961 | | | 73.2112 | | 0.024208372 | 0.8370825 | | |  |  |
| RC-RS | miR5083 | | 2.5527 | | | 1.8145 | | -0.841852152 | 0.2182228 | | |  |  |
| RC-RS | miR5084 | | 4.3396 | | | 6.2324 | | 0.892750833 | 0.0431121 | | |  |  |
| RC-RS | miR-5108 | | 386.1353 | | | 450.9432 | | 0.382655777 | 5.20E-15 | | |  |  |
| RC-RS | miR-5110 | | 508.749 | | | 358.4825 | | -0.863391003 | 1.56E-71 | | |  |  |
| RC-RS | miR-5119 | | 161.7548 | | | 168.512 | | 0.10093435 | 0.1944826 | | |  |  |
| RC-RS | miR-5124a | | 65.689 | | | 87.0172 | | 0.693461187 | 1.65E-09 | | |  |  |
| RC-RS | miR-5129-5p | | 623.9599 | | | 540.5638 | | -0.353849024 | 1.32E-17 | | |  |  |
| RC-RS | miR5139 | | 18.2942 | | | 20.8273 | | 0.319831613 | 0.1584603 | | |  |  |
| RC-RS | miR5141 | | 5.0203 | | | 3.5501 | | -0.854608491 | 0.0792347 | | |  |  |
| RC-RS | miR-5187-5p | | 15.9968 | | | 16.4883 | | 0.074636101 | 0.765571 | | |  |  |
| RC-RS | miR5246 | | 26.0373 | | | 26.9809 | | 0.087798141 | 0.652627 | | |  |  |
| RC-RS | miR529-5p | | 3.829 | | | 5.1279 | | 0.720388862 | 0.1325519 | | |  |  |
| RC-RS | miR5304-5p | | 83.3024 | | | 82.836 | | -0.013847332 | 0.8985627 | | |  |  |
| RC-RS | miR530b | | 380.6045 | | | 266.4162 | | -0.87973299 | 8.60E-56 | | |  |  |
| RC-RS | miR533e | | 360.9489 | | | 296.2372 | | -0.487282159 | 1.06E-18 | | |  |  |
| RC-RS | miR-5359-5p | | 2.6378 | | | 3.8657 | | 0.942615124 | 0.0956003 | | |  |  |
| RC-RS | miR-5398-3p | | 410.641 | | | 327.4781 | | -0.558119964 | 9.24E-27 | | |  |  |
| RC-RS | miR-5466 | | 1.4465 | | | 1.0256 | | -0.848085406 | 0.3526746 | | |  |  |
| RC-RS | miR-5468 | | 11.2318 | | | 9.5459 | | -0.401112926 | 0.1959217 | | |  |  |
| RC-RS | miR5485 | | 103.3835 | | | 122.9917 | | 0.428326619 | 5.37E-06 | | |  |  |
| RC-RS | miR5568f-5p | | 2.1272 | | | 2.0512 | | -0.089728008 | 0.8922233 | | |  |  |
| RC-RS | miR-5616-3p | | 1.1062 | | | 1.2623 | | 0.325563741 | 0.7353699 | | |  |  |
| RC-RS | miR-5622-3p | | 3.1483 | | | 2.7612 | | -0.323572246 | 0.5768506 | | |  |  |
| RC-RS | miR5662 | | 1321.8625 | | | 1668.395 | | 0.574205662 | 1.32E-108 | | |  |  |
| RC-RS | miR5667-3p | | 2.7229 | | | 3.8657 | | 0.864304429 | 0.1235038 | | |  |  |
| RC-RS | miR5673 | | 308.2786 | | | 232.8085 | | -0.692507656 | 6.70E-30 | | |  |  |
| RC-RS | miR-57 | | 39.7367 | | | 39.6035 | | -0.008281095 | 0.9570735 | | |  |  |
| RC-RS | miR-5703 | | 585.8399 | | | 485.182 | | -0.464953616 | 5.56E-27 | | |  |  |
| RC-RS | miR5718 | | 60.6687 | | | 73.2112 | | 0.463468974 | 0.0001546 | | |  |  |
| RC-RS | miR5721 | | 613.579 | | | 529.2823 | | -0.364487809 | 2.87E-18 | | |  |  |
| RC-RS | miR5773 | | 24.9312 | | | 22.2474 | | -0.280899339 | 0.171893 | | |  |  |
| RC-RS | miR5813 | | 978.6973 | | | 705.5257 | | -0.807169826 | 2.69E-120 | | |  |  |
| RC-RS | miR5815 | | 21.7829 | | | 22.4052 | | 0.069470385 | 0.7456448 | | |  |  |
| RC-RS | miR-5866 | | 10.466 | | | 11.9915 | | 0.335580434 | 0.2632496 | | |  |  |
| RC-RS | miR-6041 | | 6.637 | | | 5.049 | | -0.674459644 | 0.1044398 | | |  |  |
| RC-RS | miR-6076 | | 71.9005 | | | 64.7698 | | -0.257590033 | 0.0330213 | | |  |  |
| RC-RS | miR-6087 | | 8.8493 | | | 8.2047 | | -0.186529495 | 0.5841932 | | |  |  |
| RC-RS | miR-6129 | | 38.8858 | | | 35.2644 | | -0.241093967 | 0.1415133 | | |  |  |
| RC-RS | miR6221-5p | | 2.8079 | | | 3.1557 | | 0.287998803 | 0.6259832 | | |  |  |
| RC-RS | miR6250 | | 6.0413 | | | 8.9147 | | 0.959595221 | 0.0096638 | | |  |  |
| RC-RS | miR6300 | | 287.9422 | | | 346.2543 | | 0.45482018 | 6.14E-16 | | |  |  |
| RC-RS | miR6478 | | 21.1872 | | | 17.2772 | | -0.503149364 | 0.0274785 | | |  |  |
| RC-RS | miR-650 | | 30.0365 | | | 20.5907 | | -0.931211555 | 3.27E-06 | | |  |  |
| RC-RS | miR-6547-5p | | 473.6922 | | | 369.7639 | | -0.610886218 | 5.60E-36 | | |  |  |
| RC-RS | miR-6633-5p | | 8.1686 | | | 6.7847 | | -0.457813637 | 0.2108568 | | |  |  |
| RC-RS | miR-6640-5p | | 0.936 | | | 1.1834 | | 0.578425745 | 0.5680965 | | |  |  |
| RC-RS | miR-664-1-5p | | 11.9125 | | | 12.7804 | | 0.173441511 | 0.5446397 | | |  |  |
| RC-RS | miR-670-5p | | 142.6948 | | | 135.0621 | | -0.135581118 | 0.1095097 | | |  |  |
| RC-RS | miR-6763-5p | | 2291.2 | | | 3322.6685 | | 0.916706574 | 0 | | |  |  |
| RC-RS | miR-6802-5p | | 426.2124 | | | 328.5037 | | -0.642200055 | 1.43E-35 | | |  |  |
| RC-RS | miR-6833-5p | | 35.3121 | | | 30.6887 | | -0.346099262 | 0.0466478 | | |  |  |
| RC-RS | miR-6867-5p | | 10.3809 | | | 8.5203 | | -0.487134457 | 0.1345751 | | |  |  |
| RC-RS | miR-6868-5p | | 129.4208 | | | 148.3158 | | 0.336094707 | 7.56E-05 | | |  |  |
| RC-RS | miR-6899-5p | | 1207.4174 | | | 1175.4817 | | -0.066110939 | 0.0222984 | | |  |  |
| RC-RS | miR-6954-5p | | 253.9065 | | | 340.4163 | | 0.723128046 | 2.55E-35 | | |  |  |
| RC-RS | miR-6960-5p | | 570.9493 | | | 765.9565 | | 0.724661529 | 1.49E-77 | | |  |  |
| RC-RS | miR-6971-5p | | 17.5284 | | | 17.6717 | | 0.020080809 | 0.9348145 | | |  |  |
| RC-RS | miR-6999-5p | | 16.5073 | | | 18.4606 | | 0.275821578 | 0.2503706 | | |  |  |
| RC-RS | miR-7 | | 7.0624 | | | 7.5736 | | 0.172354108 | 0.6445938 | | |  |  |
| RC-RS | miR-7016-5p | | 27.1435 | | | 31.241 | | 0.346746791 | 0.0615798 | | |  |  |
| RC-RS | miR-7040-5p | | 26.8031 | | | 35.8956 | | 0.720400264 | 6.10E-05 | | |  |  |
| RC-RS | miR-7044-5p | | 242.3344 | | | 239.1987 | | -0.032121136 | 0.6173053 | | |  |  |
| RC-RS | miR-7078-5p | | 366.3946 | | | 258.6849 | | -0.858520673 | 1.64E-51 | | |  |  |
| RC-RS | miR-7084-5p | | 4.5097 | | | 6.7058 | | 0.978486637 | 0.022578 | | |  |  |
| RC-RS | miR-7211-3p | | 309.4698 | | | 341.8364 | | 0.245327521 | 9.52E-06 | | |  |  |
| RC-RS | miR-7233-5p | | 96.4062 | | | 96.6419 | | 0.00602242 | 0.9535857 | | |  |  |
| RC-RS | miR-744-5p | | 128.3998 | | | 96.8786 | | -0.694733474 | 1.95E-13 | | |  |  |
| RC-RS | miR-7447-3p | | 167.1154 | | | 237.3842 | | 0.865661011 | 2.52E-34 | | |  |  |
| RC-RS | miR-7472-5p | | 34.9717 | | | 29.8998 | | -0.3864386 | 0.027697 | | |  |  |
| RC-RS | miR7494b | | 11.2318 | | | 11.597 | | 0.078915298 | 0.7922617 | | |  |  |
| RC-RS | miR-7515 | | 312.2778 | | | 352.5656 | | 0.29926997 | 4.89E-08 | | |  |  |
| RC-RS | miR7545 | | 19.06 | | | 25.2452 | | 0.693140141 | 0.0011869 | | |  |  |
| RC-RS | miR-7660-3p | | 1.0211 | | | 1.1045 | | 0.193635079 | 0.8527082 | | |  |  |
| RC-RS | miR-767 | | 566.9501 | | | 821.6538 | | 0.915116592 | 2.44E-126 | | |  |  |
| RC-RS | miR7696a-3p | | 915.9014 | | | 1006.1019 | | 0.231659687 | 6.77E-13 | | |  |  |
| RC-RS | miR7732-3p | | 207.3627 | | | 158.1772 | | -0.667760326 | 2.15E-19 | | |  |  |
| RC-RS | miR774b-5p | | 6.637 | | | 9.2303 | | 0.813464554 | 0.0234568 | | |  |  |
| RC-RS | miR7760-5p | | 10.5511 | | | 7.968 | | -0.692529623 | 0.0357794 | | |  |  |
| RC-RS | miR7782-3p | | 1.4465 | | | 2.5245 | | 1.373474965 | 0.0618121 | | |  |  |
| RC-RS | miR7783-3p | | 5.3606 | | | 5.9169 | | 0.243514806 | 0.5675506 | | |  |  |
| RC-RS | miR-7865 | | 2754.5964 | | | 2211.2467 | | -0.54188253 | 3.03E-160 | | |  |  |
| RC-RS | miR8144 | | 37.184 | | | 39.6035 | | 0.155473146 | 0.3361039 | | |  |  |
| RC-RS | miR8148 | | 2.893 | | | 2.0512 | | -0.848085406 | 0.1866504 | | |  |  |
| RC-RS | miR8154 | | 2.9781 | | | 3.8657 | | 0.643353171 | 0.2412743 | | |  |  |
| RC-RS | miR8155 | | 8.3388 | | | 7.1791 | | -0.369317332 | 0.3032298 | | |  |  |
| RC-RS | miR8175 | | 160.1381 | | | 150.8403 | | -0.147521713 | 0.065424 | | |  |  |
| RC-RS | miR8176 | | 2.1272 | | | 2.6823 | | 0.571857033 | 0.3848375 | | |  |  |
| RC-RS | miR828-3p | | 14.4652 | | | 11.8337 | | -0.495219847 | 0.0727767 | | |  |  |
| RC-RS | miR-8309-3p | | 7.0624 | | | 8.5992 | | 0.485576271 | 0.1774605 | | |  |  |
| RC-RS | miR831-5p | | 5.5308 | | | 4.9702 | | -0.263579751 | 0.5443385 | | |  |  |
| RC-RS | miR-8413-5p | | 90.7903 | | | 102.6377 | | 0.302499168 | 0.0029493 | | |  |  |
| RC-RS | miR-8442-3p | | 35.6524 | | | 44.8892 | | 0.568188883 | 0.0003281 | | |  |  |
| RC-RS | miR845a | | 10.1256 | | | 9.7825 | | -0.085017911 | 0.7862622 | | |  |  |
| RC-RS | miR-8516 | | 134.6113 | | | 95.3797 | | -0.849704672 | 1.24E-19 | | |  |  |
| RC-RS | miR8581 | | 0.8509 | | | 1.2623 | | 0.972700522 | 0.3378679 | | |  |  |
| RC-RS | miR8638 | | 420.5114 | | | 407.474 | | -0.077674911 | 0.113437 | | |  |  |
| RC-RS | miR894 | | 1137.9845 | | | 792.1484 | | -0.893456024 | 1.22E-167 | | |  |  |
| RC-RS | miR-9277 | | 27.4838 | | | 26.8231 | | -0.060013266 | 0.7528719 | | |  |  |
| RC-RS | miR-9422 | | 170.8593 | | | 196.4396 | | 0.344085321 | 3.16E-06 | | |  |  |
| RC-RS | miR-944 | | 1.5316 | | | 1.9723 | | 0.623697159 | 0.4214669 | | |  |  |
| RC-RS | miR9484 | | 574.0976 | | | 725.4852 | | 0.577216761 | 9.92E-49 | | |  |  |
| RC-RS | miR9722 | | 22.889 | | | 28.0064 | | 0.497642344 | 0.0123746 | | |  |  |
| RC-RS | miR9738 | | 11.9976 | | | 16.725 | | 0.819301014 | 0.0020944 | | |  |  |
| RC-RS | miR9748 | | 2993.0166 | | | 2346.4665 | | -0.600227228 | 1.73E-210 | | |  |  |
| RC-RS | miR-9835-3p | | 17.3582 | | | 20.354 | | 0.392666179 | 0.0892809 | | |  |  |
| RC-RS | miR-9894 | | 21.953 | | | 22.8785 | | 0.101843041 | 0.6312312 | | |  |  |
| RC-RS | miR-9b-3p | | 4.6799 | | | 5.049 | | 0.187225625 | 0.6842803 | | |  |  |
| RC-RS | miR-D13-5p | | 17.103 | | | 16.9616 | | -0.020475056 | 0.9307468 | | |  |  |
| RC-RS | miR-H1 | | 2.3825 | | | 2.5245 | | 0.142780879 | 0.8295641 | | |  |  |
| RC-RS | miR-H11 | | 24.3355 | | | 17.2772 | | -0.844828497 | 0.0001269 | | |  |  |
| RC-RS | miR-H8 | | 2.893 | | | 3.3134 | | 0.3346301 | 0.5625593 | | |  |  |
| RC-RS | miR-I2 | | 13.1888 | | | 11.9126 | | -0.250998961 | 0.3727796 | | |  |  |
| RC-RS | miR-I5-3p | | 28.8453 | | | 33.2133 | | 0.34775701 | 0.053231 | | |  |  |
| RC-RS | miR-M23-1-5p | | 441.8688 | | | 488.4955 | | 0.2474128 | 9.44E-08 | | |  |  |
