# Supplementary material for: Identification of Salt Tolerance-related microRNAs and Their Targets in Maize (Zea mays L.) Using High-throughput Sequencing and Degradome Analysis
Source: Front Plant Sci. 2017 May 26;8:864. doi: 10.3389/fpls.2017.00864 (PMC5445174; doi:10.3389/fpls.2017.00864)
Supplement: Supplementary file 9 [file Table_5.DOC]

**Table S5** Differentially expressed novel miRNAs in response to salt stress in maize.

| **NO.** | **miRNA ID** | **sequence** | **Length**  **(nt)** | **leaves** | | | | **sig-**  **lable** | **roots** | | | | **sig-**  **lable** |
| --- | --- | --- | --- | --- | --- | --- | --- | --- | --- | --- | --- | --- | --- |
|  |  |  |  | **CK** | **Salt** | **fold-change(log_2_ LS/LC)** | **p-value** |  | **CK** | **Salt** | **fold-change(log_2_ RS/RC)** | **p-value** |  |
| 1 | novel_mir_115 | TGGTGATCATTGGATTGGCGTC | 22 | 8.65 | 2.75 | -1.65 | <0.001 | ** | 6.89 | 16.02 | 1.22 | <0.001 | ** |
| 2 | novel_mir_122 | AGAGGATCTATGGGGGAGGAA | 21 | 1.68 | 0.49 | -1.79 | 0.004 | ** | - | - | - | - |  |
| 3 | novel_mir_148 | GGAGGTGCTGCGACGAGGACGA | 22 | 4.96 | 0.73 | -2.77 | <0.001 | ** | 1.02 | 0.01 | -6.67 | <0.001 | ** |
| 4 | novel_mir_150 | TTAAGTTCGTCGAAAATATGC | 21 | 9.07 | 0.01 | -9.83 | <0.001 | ** | 1.02 | 0.01 | -6.67 | <0.001 | ** |
| 5 | novel_mir_151 | AGAGGGGGATTAGAGGGGTTT | 21 | 0.84 | 2.83 | 1.75 | <0.001 | ** | - | - | - | - |  |
| 6 | novel_mir_157 | TAATGGATCAGGAGGTTTGCT | 21 | - | - | - | - |  | 0.68 | 2.37 | 1.80 | <0.001 | ** |
| 7 | novel_mir_17 | AGGAGGGCTGTGGATGAGGCTG | 22 | 1.76 | 0.01 | -7.46 | <0.001 | ** | 1.36 | 0.01 | -7.09 | <0.001 | ** |
| 8 | novel_mir_175 | TGAAGAGAATTGAGGGGGCTA | 21 | 9.15 | 0.01 | -9.84 | <0.001 | ** | 3.74 | 1.74 | -1.11 | 0.003 | ** |
| 9 | novel_mir_179 | TAAAGTGGCTTATAATTTGGA | 21 | 4.87 | 0.01 | -8.93 | <0.001 | ** | 0.01 | 1.66 | 7.37 | <0.001 | ** |
| 10 | novel_mir_180 | GCCGAGCGGACGGCGGGCGGA | 21 | 0.42 | 1.86 | 2.15 | <0.001 | ** | - | - | - | - |  |
| 11 | novel_mir_189 | TGGAGGGGATTGAGGGGCATA | 21 | 2.44 | 0.01 | -7.93 | <0.001 | ** | 0.01 | 1.34 | 7.07 | <0.001 | ** |
| 12 | novel_mir_200 | TCACCAGTTAGGAGGAGGATGA | 22 | 1.01 | 0.01 | -6.66 | <0.001 | ** | 0.01 | 1.03 | 6.68 | <0.001 | ** |
| 13 | novel_mir_205 | TGGAGAGAATTGAGGGGGCTA | 21 | 1.43 | 0.01 | -7.16 | <0.001 | ** | 0.01 | 1.26 | 6.98 | <0.001 | ** |
| 14 | novel_mir_212 | ACCGGAGGGGATTGGAGGCGC | 21 | - | - | - | - |  | 2.55 | 0.79 | -1.69 | <0.001 | ** |
| 15 | novel_mir_220 | AAAGGGGAGATTGTAAGTGCAA | 22 | - | - | - | - |  | 0.51 | 2.05 | 2.01 | <0.001 | ** |
| 16 | novel_mir_230 | GACGTACTGCAGAGGATCCGGTT | 23 | 1.18 | 7.20 | 2.61 | <0.001 | ** | - | - | - | - |  |
| 17 | novel_mir_236 | GGGGATGGCGTCGTAGCTCGG | 21 | 21.16 | 9.94 | -1.09 | <0.001 | ** | - | - | - | - |  |
| 18 | novel_mir_237 | CAGTGAGACCCTGCAGAAGCTG | 22 | - | - | - | - |  | 2.64 | 1.03 | -1.36 | 0.003 | ** |
| 19 | novel_mir_250 | TCAACGGCAGATGTAGAGGGTT | 22 | 13.19 | 0.01 | -10.36 | <0.001 | ** | 3.40 | 0.01 | -8.41 | <0.001 | ** |
| 20 | novel_mir_273 | TCGAAGGGGATTGGAGAGGTT | 21 | - | - | - | - |  | 8.42 | 18.62 | 1.14 | <0.001 | ** |
| 21 | novel_mir_29 | AGATCATGTGGCAGTTTCATT | 21 | 52.49 | 49.88 | -0.07 | 0.369 |  | 7.83 | 25.17 | 1.68 | <0.001 | ** |
| 22 | novel_mir_316 | AGCAAGAGGATTGGAGGGGCT | 21 | - | - | - | - |  | 1.62 | 3.47 | 1.10 | 0.004 | ** |
| 23 | novel_mir_325 | CCGTGGCGTGTGGGATGGCGTG | 22 | 0.01 | 2.83 | 8.14 | <0.001 | ** | 4.51 | 1.10 | -2.03 | <0.001 | ** |
| 24 | novel_mir_330 | TGGAGAGGATTGTAGGGGCTA | 21 | 0.01 | 4.1234 | 8.69 | <0.001 | ** | 4.08 | 0.01 | -8.67 | <0.001 | ** |
| 25 | novel_mir_335 | ACTTAGGAACGGAGGGAGTAC | 21 | - | - | - | - |  | 1.19 | 0.01 | -6.90 | <0.001 | ** |
| 26 | novel_mir_36 | TGGAGAAGCAGGACACGTGAG | 21 | 168.40 | 433.12 | 1.36 | <0.001 | ** | - | - | - | - |  |
| 27 | novel_mir_37 | TTAAGGTAATTGAGGACACCA | 21 | 1.68 | 4.04 | 1.27 | <0.001 | ** | - | - | - | - |  |
| 28 | novel_mir_388 | ACTCGCTATATGTTCGGACGCT | 22 | 0.01 | 3.15 | 8.30 | <0.001 | ** | - | - | - | - |  |
| 29 | novel_mir_390 | AAAGTGGCTTATAATTTGGAA | 21 | 0.01 | 3.07 | 8.26 | <0.001 | ** | - | - | - | - |  |
| 30 | novel_mir_41 | TTCTAGAGTGAAGTAGAACGGAG | 23 | 0.84 | 2.75 | 1.71 | <0.001 | ** | 1.02 | 0.01 | -6.67 | <0.001 | ** |
| 31 | novel_mir_448 | ATCACGGGAAGATTGGAGGGG | 21 | 0.01 | 3.80 | 8.57 | <0.001 | ** | 0.01 | 1.50 | 7.23 | <0.001 | ** |
| 32 | novel_mir_538 | AAGAACTCTGTTGGTGGCTGA | 21 | 0.01 | 0.01 | 0.00 | - |  | 0.77 | 2.52 | 1.72 | <0.001 | ** |
| 33 | novel_mir_57 | TAGAGGCGGTTCTTTATGAAA | 21 | 0.92 | 2.67 | 1.53 | 0.001 | ** | - | - | - | - |  |
| 34 | novel_mir_61 | ACACCACCAGAACTAAGGGCA | 21 | 4.79 | 1.70 | -1.50 | <0.001 | ** | - | - | - | - |  |
| 35 | novel_mir_76 | TTGGATTTTGATTGGATGCAC | 21 | 2.30 | 5.68 | 1.31 | <0.001 | ** | - | - | - | - |  |
| 36 | novel_mir_91 | ATTGGAGGGGATTGAGGAGGCT | 22 | 5.11 | 1.97 | -1.37 | <0.001 | ** | - | - | - | - |  |
| 37 | novel_mir_95 | CAAGTCGAGGGCAGACCAGGCG | 22 | 2.27 | 0.01 | -7.83 | <0.001 | ** | 0.01 | 1.10 | 6.79 | <0.001 | ** |
