# Supplementary material for: Identification of Salt Tolerance-related microRNAs and Their Targets in Maize (Zea mays L.) Using High-throughput Sequencing and Degradome Analysis
Source: Front Plant Sci. 2017 May 26;8:864. doi: 10.3389/fpls.2017.00864 (PMC5445174; doi:10.3389/fpls.2017.00864)
Supplement: Supplementary file 10 [file Table_6.DOC]

**Table S6** Primers of target genes; IR (internal reference)

| **miRNA** | **Target genes** | **Tag primers** | **Primer sequence (5'- 3')** | **Size of amplified products (bp)** |
| --- | --- | --- | --- | --- |
| Zea mays alpha-tubulin 5(IR) | Zea mays alpha-tubulin 5(IR) | forward | GCCGTTGCCGAGGTGTTC | 148 |
|  |  | reverse | GTCCTTCTCAAGAGCAGCCAAGT |  |
| mir_29 | GRMZM2G110688 | forward | GGGATCTGAGAGCGACAAAA | 244 |
|  |  | reverse | GACAAGAGCATTGCATGGTG |  |
|  | GRMZM2G362718 | forward | CTCCCTGCCTTCTCTCCTTC | 181 |
|  |  | reverse | GGGTCCACTCCATCATCATC |  |
|  | GRMZM2G084296 | forward | CTCAATCCCCATTCCGTCA | 149 |
|  |  | reverse | AGGAGGAGGAGGAGCTTGGA |  |
| mir_36 | GRMZM2G149952 | forward | TGGTGACTGGTTGATTGGTG | 242 |
|  |  | reverse | GGATGGAGCAGAAGAACAGG |  |
|  | GRMZM2G055489 | forward | AGGGCTGAGGTTCCAAAGTC | 216 |
|  |  | reverse | TCCCCACCCAACTATCTGAA |  |
| mir_250 | GRMZM2G012479 | forward | GGGCATCCACTTTTGTGC | 241 |
|  |  | reverse | TGCTGTTGTCAACCCACATT |  |
|  | GRMZM2G458728 | forward | ATTGTGATTCATTGGCAGCA | 149 |
|  |  | reverse | ATTTGTCATTGCTCCGAACAG |  |
| mir_316 | GRMZM2G320298 | forward | CAGCACGGCTTCTTCACC | 166 |
|  |  | reverse | GAACAACTCGACGGCTCAAC |  |
|  | GRMZM2G306345 | forward | GACCTGACGCAGATGACCTT | 196 |
|  |  | reverse | AGCAGTGTCCCAAAACCAAC |  |
|  | GRMZM2G154628 | forward | CCGCCTACCACCAGTACG | 188 |
|  |  | reverse | GGTCCGAAGTAGAAAACAGCAG |  |
| mir_17 | GRMZM2G154667_T01 | forward | TGCCTGAAAGTTGGATAGGC | 247 |
|  |  | reverse | TGAAGCCCTTTTTGTTCAGTC |  |
| mir_189 | GRMZM2G141185_T02 | forward | CTGGGAAAATCCACGTTCAA | 150 |
|  |  | reverse | TAACCGGCCTTGTTCTTTCA |  |
|  | GRMZM2G130062_T01 | forward | GGGGAAGTCATAAAAGCTGGA | 163 |
|  |  | reverse | GCAAGACCAAGGTCATTCTGA |  |
|  | GRMZM2G123652_T02 | forward | AGGTACTGGTGGTGCTGGTC | 222 |
|  |  | reverse | AGCACTAACAGTTGGCACACC |  |
| mir_205 | GRMZM2G375504_T02 | forward | GTTTCATCAGGCAGGAGCAT | 213 |
|  |  | reverse | TGTTCGACCGCAAAACAAAT |  |
| mir_330 | GRMZM2G046092_T01 | forward | ATGCCAACACTCCAGTCTCC | 219 |
|  |  | reverse | CACCCTTCAAAGAGGAAGGTC |  |
|  | GRMZM5G823004_T01 | forward | GCCCTTGAGAAACCATCAGA | 161 |
|  |  | reverse | TTCCAGTGAGGGCTTCTTTG |  |
|  | GRMZM2G305167_T01 | forward | ATGCCAACACTCCAGTCTCC | 182 |
|  |  | reverse | CTCCACCCCAATCATCTGAA |  |
|  | GRMZM2G012324_T01 | forward | GGTGTAGCCTTTCCTCGTCA | 228 |
|  |  | reverse | GCCCTGGAGTTCCAAATCTC |  |
|  | GRMZM2G017290_T01 | forward | GGCGTCTATTAGGGCTTGTTC | 158 |
|  |  | reverse | GCTCCATCTAAGGTCTTGTTCG |  |
|  | GRMZM5G874478_T02 | forward | GGTGGTGACCGATACTCTGG | 225 |
|  |  | reverse | AGCAGCTGATTTCTCACGGTA |  |
|  | GRMZM2G012160_T01 | forward | GCCTGTTGAAGAGGGTGCTA | 167 |
|  |  | reverse | GACACAAACGAACAAGCCCTA |  |
|  | GRMZM2G466743_T01 | forward | TCCATTCTCCAGGTTGAAGC | 172 |
|  |  | reverse | TCCACCCCAATCATCTGAAG |  |
